# Thermal stability and kinetic constants for 129 variants of a family 1 glycoside hydrolase reveal that enzyme activity and stability can be separately designed

**DOI:** 10.1371/journal.pone.0176255

**Published:** 2017-05-22

**Authors:** Dylan Alexander Carlin, Siena Hapig-Ward, Bill Wayne Chan, Natalie Damrau, Mary Riley, Ryan W. Caster, Bowen Bethards, Justin B. Siegel

**Affiliations:** 1 Biophysics Graduate Group, University of California, Davis, California, United States of America; 2 Genome Center, University of California, Davis, California, United States of America; 3 Department of Biochemistry & Molecular Medicine, University of California, Davis, California, United States of America; 4 Genetics Graduate Group, University of California, Davis, California, United States of America; 5 Department of Chemistry, University of California, Davis, California, United States of America; Universidade Nova de Lisboa Instituto de Tecnologia Quimica e Biologica, PORTUGAL

## Abstract

Accurate modeling of enzyme activity and stability is an important goal of the protein engineering community. However, studies seeking to evaluate current progress are limited by small data sets of quantitative kinetic constants and thermal stability measurements. Here, we report quantitative measurements of soluble protein expression in *E*. *coli*, thermal stability, and Michaelis-Menten constants (*k*_cat_, K_M_, and *k*_cat_/K_M_) for 129 designed mutants of a glycoside hydrolase. Statistical analyses reveal that functional T_m_ is independent of *k*_cat_, K_M_, and *k*_cat_/K_M_ in this system, illustrating that an individual mutation can modulate these functional parameters independently. In addition, this data set is used to evaluate computational predictions of protein stability using the established Rosetta and FoldX algorithms. Predictions for both are found to correlate only weakly with experimental measurements, suggesting improvements are needed in the underlying algorithms.

## Introduction

Enzymes are proteins that have evolved to be the most proficient catalysts known [1]. It is widely hypothesized that functional proteins such as enzymes must trade thermodynamic stability for properties such as pre-ordered active sites to achieve their extraordinary catalytic proficiency [2,3]. Tradeoffs between stability and catalytic proficiency introduce additional complexity to the computational design of enzymes because designed mutations must be compatible with both a targeted thermal activity (T_m_) and a catalytic activity.

One major challenge to evaluating and improving the predictions made by current enzyme design algorithms is the lack of large data sets for which enzyme functional parameters and protein stability have been measured quantitatively. Studies that have explored mutagenesis of active sites in exquisite detail have sample sizes that are too small (n ~ 30) to allow for generalizable predictions outside the few sequence positions mutated [4]. In contrast, studies that cover >90% of possible point mutations to a particular enzyme measure activity only qualitatively or convolve independent parameters such as stability and activity into a single measurement [5,6], reducing their utility in training enzyme design algorithms seeking to make quantitative predictions of enzyme functional parameters. Large data sets such as the ProTherm database [7], with roughly 10,000 characterized point mutations from nearly 1000 individual proteins, do not contain enzyme kinetic data. In addition, this data was collected under a wide variety of experimental conditions without regard to standardization. Furthermore, the ProTherm database has been extensively used to parameterize force fields used in molecular modeling [8,9], leading to a likely bias in assessment of current enzyme design algorithms using this data set.

**Fig 1:**
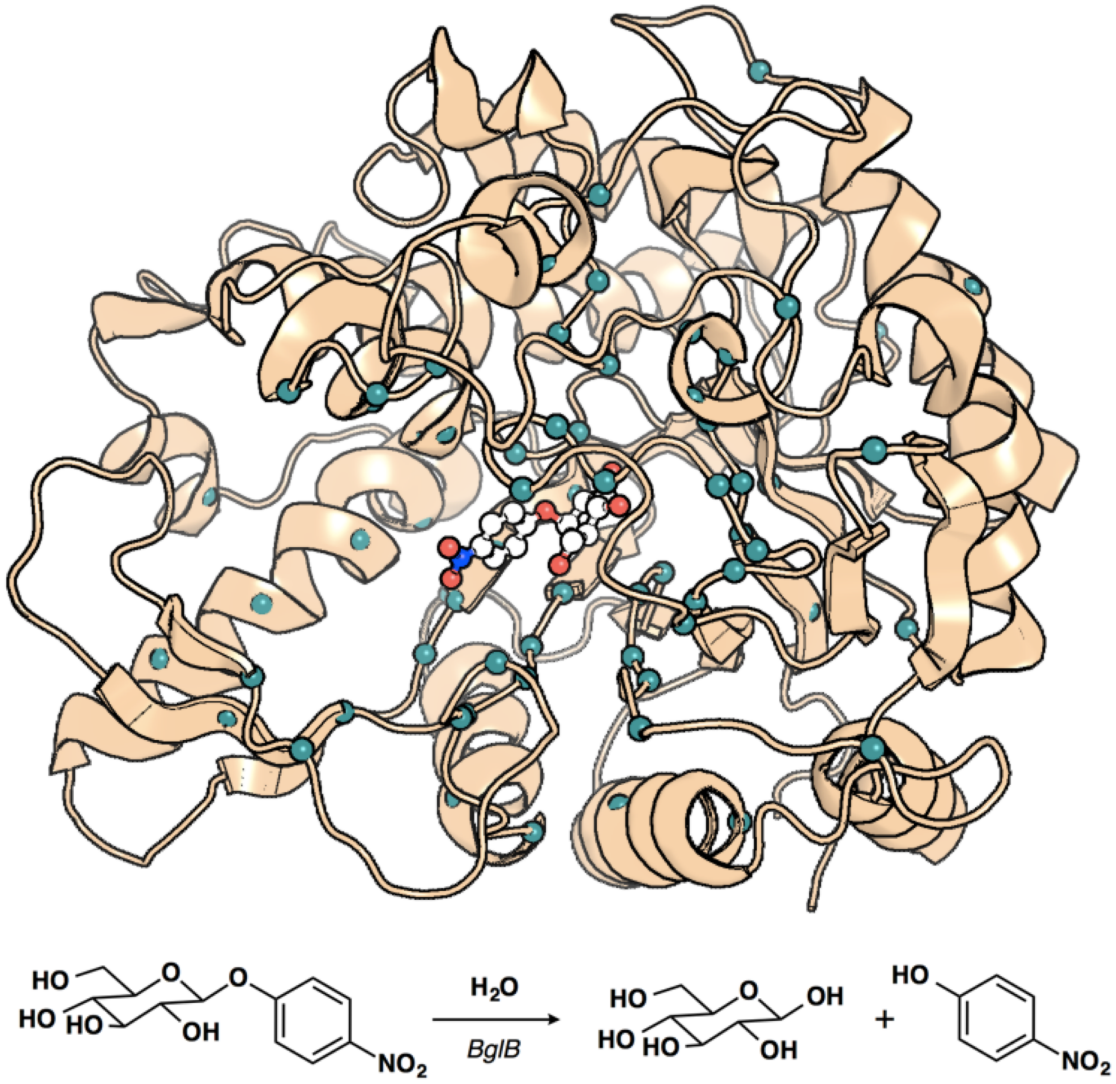
Overview of the modeled BglB-pNPG complex showing positions mutated in this study and reaction used to determine functional properties of individual mutants. PyMOL rendering [32] of modeled BglB in complex with pNPG showing the 68 sequence positions selected for mutation in this study (teal spheres) and the modeled transition-state structure (white ball and stick model). Below, reaction scheme of the hydrolysis of pNPG by BglB used to determine functional T_m_ and kinetic parameters *k*_cat_, K_M_, and *k*_cat_/K_M_.

We previously reported soluble protein expression in *E*. *coli* and Michaelis-Menten constants (*k*_cat_, K_M_, and *k*_cat_/K_M_) for 100 designed mutants of a β-glucosidase (BglB) from the bacterium *Paenibacillus polymyxa* on the reporter substrate 4-nitrophenyl-β-D-glucoside (pNPG) [10]. The location of the sites mutated and the reaction (pNPG hydrolysis) used to determine functional parameters is illustrated in Fig 1. The study of the 100 BglB variants revealed that current algorithms do not enable robust and accurate prediction of kinetic parameters. However, machine learning analysis did indicate that algorithms that predict function based on calculated structural features could be developed with more training data. Here we perform an expanded study exploring the thermal stability of the original mutants in the BglB data set, plus 29 additional mutations.

To evaluate the ability of molecular modeling software to predict thermal stability of mutants in our data set, we modeled each of the 129 point mutations to the BglB sequence using three approaches: 1) an enzyme-specific algorithm termed RosettaDesign [11], 2) an algorithm for predicting ΔΔG of point mutations to proteins termed Rosetta ΔΔG [12], and 3) an algorithm for predicting ΔΔG of mutations to proteins using FoldX [9]. The data set of protein expression, thermal stability, *k*_cat_, K_M_, and *k*_cat_/K_M_ enabled us to evaluate the performance of these three current force-field–based approaches to modeling stability changes caused by mutations, building on previous work where we evaluated the ability of Rosetta to predict changes in kinetic constants for this model system [10]. Similar to the original study, we found only a weak correlation (PCC <0.3) between predicted and observed stability for each of these established protocols. This highlights the need for further development of algorithms for protein function prediction and the importance of large data sets that are orthogonal to the data sets used for training current algorithms.

## Materials and methods

### Mutant selection

A crystal structure of recombinant BglB in complex with the substrate analog 2-deoxy-2-fluoro-α-D-glucopyranose (PDB ID: 2JIE) was used to build models of BglB using Rosetta and FoldX. Family 1 glycoside hydrolase enzyme active sites position two like-charged residues in close proximity, creating an unfavorable electrostatic interaction, in order to present pre-ordered geometry for catalysis [1]. In BglB, the carboxyl oxygens of two catalytic glutamate residues, one functioning as a nucleophile and the other as an acid/base in a Koshland double-displacement mechanism [13], are positioned at 3.1 Å in a crystal structure of a BglB-inhibitor complex (2JIE) and 4.5 Å in the apo structure (2O9P) [14]. BglB relies on the proximity of this pair of glutamate residues to cyclically perturb the p*K*_a_ of E164 during catalysis, allowing it to act as a general acid in the glycosylation step and a general base in the product release step [15,16]. In RosettaDesign simulations, functional constraints were used to enforce catalytic distances, angles, and dihedral angles among a parameterized representation of reporter substrate pNPG, and protein side chains E164, E353, and Y295, as reported previously [10]. In the other protocols, the apo structure of BglB (2O9P) was used to build the initial models.

To select a subset of the 8,455 possible single point mutations to the native BglB sequence that could be experimentally characterized, three approaches were taken. First, all residues within 12 Å of the modeled pNPG were individually mutated to alanine. Second, a subset of mutants was chosen at random by selecting a random mutation to residues within 12 Å of the active site. Third, a model of the BglB-pNPG complex was loaded into Foldit, a graphical user interface to Rosetta, point mutations to the protein were modeled, and a subset were chosen by students learning about molecular modeling. In this approach, designed sequences had energies no more than 5 Rosetta Energy Units (REU) higher than the modeled BglB-pNPG complex, but no other rules were used to select mutations.

### Molecular cloning and mutagenesis

The BglB construct from our previous study [10] was used to generate the 29 additional mutants characterized in this study using an automated Kunkel mutagenesis procedure (Transcriptic). Individual plasmid constructs were verified by Sanger sequencing (Operon, Genscript) and sequence-perfect clones were used for subsequent characterization.

### Protein production and purification

Individual purified plasmid constructs were transformed into chemically competent *Escherichia coli* BLR (DE3) cells and plated on selection plates containing 50 μg/mL kanamycin. Single colonies were used to inoculate 5 mL Terrific Broth (Fisher BP24682) in 50 mL Falcon tubes (Fisher 14-959-49A) with breathable seals (Fisher 12-567-05). After incubation at 37°C with shaking at 300 RPM for 24 hours, cells were pelleted and media replaced with 5 mL Terrific Broth containing 1 mM isopropyl-β-D-1-thiogalactopyranoside (IPTG) and 50 μg/mL kanamycin to induce expression of BglB. After incubation at 18°C with shaking at 300 RPM for 24 hours, cells were pelleted, resuspended in enzyme storage buffer (50 mM HEPES, 150 mM sodium chloride, 25 mM EDTA, pH 7.50) and lysed with BugBuster protein extraction reagent (EMD Millipore 70584–3).

After clarification of lysis mixture by centrifugation at 14,700 RPM for 30 minutes, His-tagged BglB proteins were purified via immobilized metal ion affinity chromatography using 50 μL bed volume of Ni-NTA resin (Thermo 88221) and eluted in 300 μL enzyme storage buffer (wash buffer was the same as enzyme storage buffer except substituting 25 mM imidazole for 25 mM EDTA). Protein purity was assessed using 4–12% gradient SDS-PAGE (Life Technologies) and total protein yield determined by A280 using a BioTek Epoch spectrophotometer. All other reagents were purchased from Fisher Scientific.

### Determination of Michaelis-Menten kinetics and thermal stability for individual mutants of BglB

Kinetic constants for 10 new mutations beyond those previously characterized are included in this data set. Michaelis-Menten parameters for each mutant are determined as described previously [10]. For previously characterized mutants, kinetic constants are drawn from the publication.

For thermal stability assays, purified proteins diluted 1:10 in enzyme storage buffer (diluted protein concentration: 0.01–0.1 mg/mL) were aliquotted in triplicate into 96-well PCR plates (Fisher 14-230-232), using a volume of 50 μL per well. Proteins were thermally challenged for 30 minutes at 8 constant temperatures (lowest: 30°C, highest: 50°C, step size: 2.5°C) in a thermal cycler (BioRad) and 25 μL was immediately transferred to 96-well non-binding assay plates (Corning 3884) containing 75 μL of 100 mM pNPG (Sigma N7006) in enzyme storage buffer. Production rate of 4-nitrophenol was determined by monitoring A_420_ for 60 minute, and fitting the linear portion of the observed reaction to a straight line (Gen5).

The functional parameter T_m_ was defined as temperature at which half of the protein molecules were denatured after heat challenge. To determine the T_m_ for each BglB variant, the product formation rates from samples that had been challenged at each of 8 temperatures were fit to the logistic equation 1/(1+*e*^-*k*(T–Tm)^), where *T* is the incubation temperature measured in degrees Celsius and *k* is the kurtosis of the melting curve.

For all mutants, kinetic constants and thermal stability measurements and statistical analysis are provided in S1 Table. Additional information about experimental procedures is provided in S1 Text.

### Molecular modeling of BglB mutants

Three molecular modeling approaches were taken in this study. First, a model of BglB-pNPG complex was generated using RosettaDesign as described previously [10]. Individual mutations were generated by replacement of the target amino acid with the lowest-energy rotamer of the designed amino acid, followed by 100 random combined translation and rotation moves of the modeled pNPG and Monte Carlo optimization of the total system energy by 10 iterations of rotamer repacking and gradient-based minimization. For each protein, 100 structures were generated and the lowest 10 models in total system energy were selected for further analysis. For the 10 low-energy models, 60 features (*e*.*g*., total system energy, protein-ligand interface energy, hydrogen-bonding energy, and packing around the modeled pNPG) were calculated and averaged (see S2 Table for a list of the features used and their Pearson correlations to individual experimental values). This algorithm approximates protocols used in successful enzyme design efforts using Rosetta [17–19].

Second, mutations were generated and scored using the Rosetta ddg_monomer application, with recommended settings previously validated on experimental data [20], and the results were averaged across all 50 iterations. The feature set for Rosetta ΔΔG contains 15 terms from the Rosetta score function, which are reported in S2 Table. Details of the underlying algorithm are given in [12].

Third, the FoldX position-specific scoring matrix (PSSM) algorithm was used. After adjusting the crystal structure 2JIE to the FoldX force field using the RepairPDB application, point mutations were modeled using the PSSM application [9] and scored on 17 score terms used by the FoldX force field (S2 Table).

For each of the three modeling approaches, Pearson correlation between each calculated feature (60 for RosettaDesign, 15 for Rosetta ΔΔG, and 17 for FoldX) and experimentally-determined T_m_ was calculated after removing features with variance of < 0.05. All resulting values are reported in S2 Table.

## Results

### Production of BglB mutants in *E*. *coli* and protein purification

**Fig 2:**
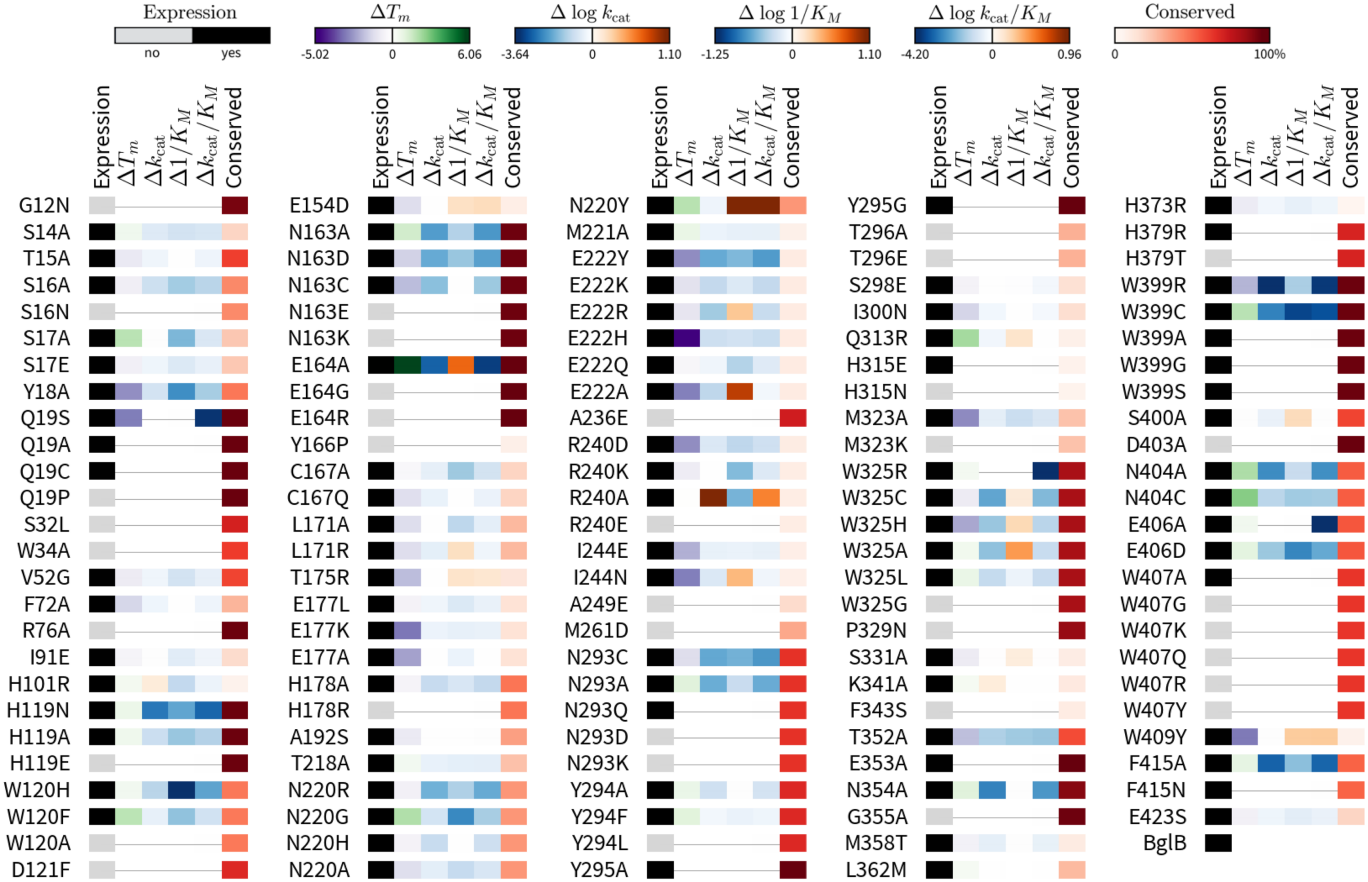
Relative effects on enzyme functional parameters for 129 mutants of BglB. Each mutant gets a bar with six colored boxes, depicting 1) soluble protein expression, 2) T_m_, 3) *k*_cat_, 4) K_M_, 5) *k*_cat_/K_M_, and 6) conservation within Pfam GH01. For expression (box 1), a black box indicates soluble expression > 0.10 mg/mL, and a white box indicates expression < 0.10 mg/mL in *E*. *coli* BLR (DE3). For T_m_ (box 2), a linear scale is used to depict change in T_m_ compared to wild type, with mutants with greater T_m_ in green, and those with lower T_m_ in purple. For *k*_cat_, 1/K_M_, and *k*_cat_/K_M_ (boxes 3–5), blue indicates lower values, and orange indicates higher values relative to the wild type value, as indicated by the color legend (top). For conservation (box 6), the frequency of native BglB residue in an alignment of the BglB sequence to 1,554 sequences from Pfam GH01 is shown, on a linear scale from 0% to 100%. The quantitative measurements used to produce this illustration are provided in S1 Table.

Of 129 mutant proteins produced, 92 expressed and purified as soluble protein (Fig 2, “Expression” column). The remaining 37 mutants did not visibly appear after SDS-PAGE analysis after at least 2 independent production attempts. Gel images of each protein used in this study can be found as S1 Fig.

Mutants that did not express were broadly consistent with well-established rules of protein folding [1], such as the large destabilizing effect of the introduction of proline into an alpha helix (Y166P, Q19P), the mutation of topology-defining/helix-ending proline residues (P329N), mutations from glycine (G12N, G355A), the introduction of charged residues into the hydrophobic core (A236E, F72H, N293D, N293K), and extreme amino acid volume changes in the core of the protein (*e*.*g*., small-to-large mutations like A227W).

### Kinetic constants for 10 new variants of BglB build on previous data set

Building on our previous BglB data set, we report kinetic constants for 10 mutants for which kinetic constants had not previously been determined. We found strong agreement (4% difference for *k*_cat_, 3% for K_M_, and 1% for *k*_cat_/K_M_) between values for batched replicates of the native BglB sequence and the previous values produced by a different group of researchers a year previously. The limit of detection of our assay for *k*_cat_/K_M_ is 10 M^–1^min^–1^, and the maximum *k*_cat_/K_M_ in the data set is 1,570,000 M^–1^min^–1^ (N220Y).

The most notable change in kinetic constants within the new mutants characterized in this publication is the 10-fold decrease (indicating more sensitive enzyme response to substrate concentration) for computationally-designed mutant N220Y. Interestingly, the structural hypothesis (favorable molecular interaction between the aromatic ring of the designed tyrosine and the aromatic ring of pNPG leading to a lower K_M_) for this mutation matched the intended functional effect.

Together, data for soluble expression, kinetic constants (*k*_cat_, K_M_, *k*_cat_/K_M_), and melting temperature are reported for 129 mutants of BglB. Fig 2 depicts the data set as a heat map, with values relative to native BglB. (A table of *k*_cat_, K_M_, and *k*_cat_/K_M_ values with statistical analysis can be found in S1 Table, and Michaelis-Menten plots can be found as S2 Fig).

### Functional protein melting temperature for 79 mutants of BglB

Of the 79 solubly-expressed mutants which have kinetic activity above our limit of detection for *k*_cat_/K_M_ of 10 M^–1^min^–1^, a functional melting temperature (T_m_) was determined by fitting observed rates collected from proteins incubated at 8 temperatures (30–50°C) to the logistic equation, as described in “Materials and methods”.

Ten wild type BglB replicates had an average melting temperature of 39.9 ± 0.1°C. In the mutant data set, the average melting temperature was 39.4 ± 1.8°C, and the total range observed was from 34.9 to 46.0°C (a range of ~11°C). Of 79 mutants for which T_m_ was determined, 43 mutants have a T_m_ that falls within 1°C of the wild type T_m_. Of the remaining 36 T_m_ values, 26 exhibited a lower melting temperature and 10 displayed a higher melting temperature. The highest T_m_ observed in this data set is for the mutation E164A, which increased the T_m_ to 46.0°C (+6.1°C), while the lowest T_m_ observed was for mutant E222H, which had a T_m_ of 34.9°C (–5.6°C).

### Overview of data for 129 mutants of BglB

All experimental data collected in this study is illustrated in Fig 2. This includes measured values for expression in *E*. *coli*, functional T_m_, kinetic constants *k*_cat_, K_M_, and *k*_cat_/K_M_, and sequence conservation within Pfam GH01 relative to the BglB wild type values as a heat map. A table containing experimentally-determined T_m_ values and statistical analysis is available as S1 Table, and diagnostic plots with statistical analysis as S3 Fig.

**Fig 3:**
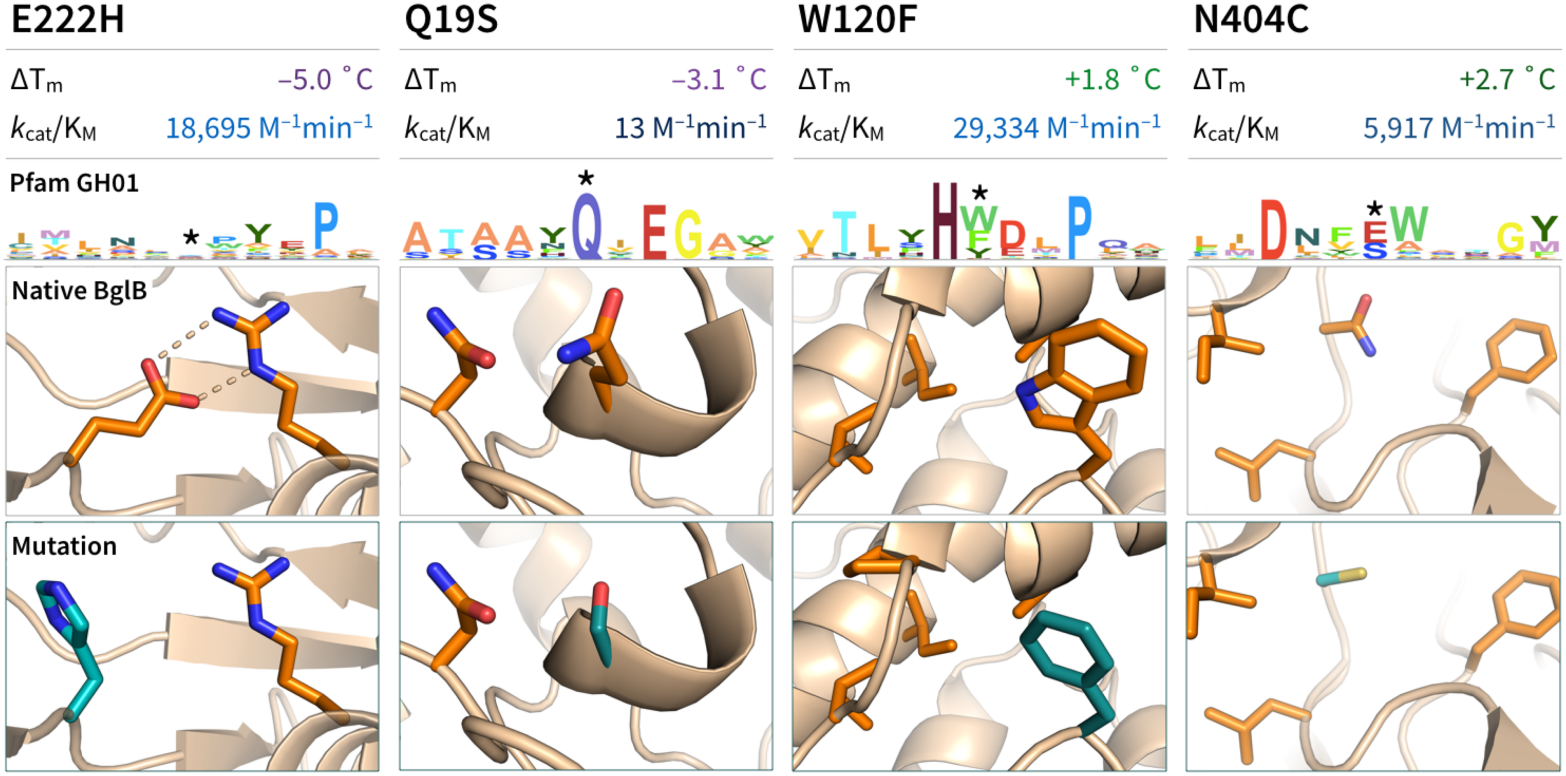
Structural analysis of Rosetta models of designed point mutants of BglB with effects on thermal stability. Four mutant panels are shown, sorted from left to right by increasing T_m_. In the top panel, experimentally-determined change in T_m_ and k_cat_/K_M_ are given. For reference, the T_m_ for the wild type sequence is 39.9°C, and the *k*_cat_/K_M_ is 174,000 M^–1^min^–1^. In the next panel down, sequence logos depict the local area of sequence conservation based on an alignment of 1,544 sequences from Pfam GH01. At bottom, depictions of the local area of the mutation in the BglB WT protein (top) and RosettaDesign model of mutation (bottom).

Some individual mutations not involving catalytic residues explicitly capture the concept of function-stability trade-offs. For mutation N404C, the functional T_m_ increased by 2.75°C while *k*_cat_ decreased by 10-fold. Similarly, the mutation W120F increased functional T_m_ by 2.6°C, while decreasing *k*_cat_ by 9-fold. Illustrations of the local area of these two mutants can be found in Fig 3. Also pictured in Fig 3 are the two mutants that most significantly decreased thermal stability: E222H (ΔT_m_ –5.0°C) and Q19S (ΔT_m_ –3.1°C). For E222H, molecular modeling indicates an unfavorable Coulombic interaction between the charged R240 and the mutant E222H being responsible for the loss of stability in this mutant. For Q19S, it is unclear what the major driving force is behind the destabilization.

Some mutations alter one functional parameter while leaving another unchanged. The mutation R240A does not change T_m_, but increases *k*_cat_ by 10-fold (see Fig 2), while Q313R increases T_m_ by 2.2°C while leaving *k*_cat_ within 10% of the *k*_cat_ of native BglB. Some mutations show greater T_m_ accompanied by a “better” functional parameter. For the Michaelis constant (K_M_) a decrease of 10-fold in mutant N220Y is accompanied by an increase in T_m_ by 1.9°C.

### Relationship between T_m_ and *k*_cat_, K_M_, and *k*_cat_/K_M_ for mutants of BglB

**Fig 4:**
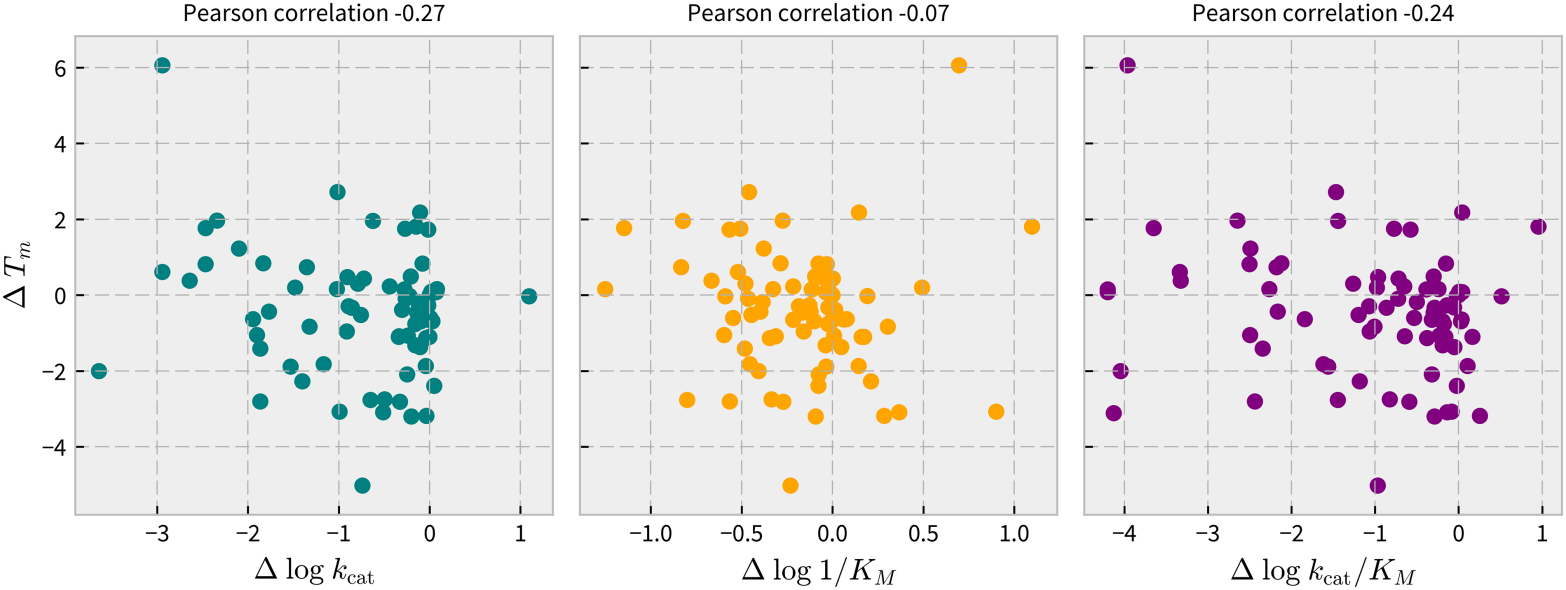
Relationship between protein melting temperature (T_m_) and kinetic constants *k*_cat_, K_M_, and *k*_cat_/K_M_ in the BglB system. T_m_ values are on a linear scale in units of degrees Celsius and values for kinetic constants are on a log scale, with units of min^–1^, mM, and M^–1^min^–1^, respectively. These parameters are not correlated in BglB (Pearson correlation < 0.25 for T_m_ versus each of the kinetic constants *k*_cat_, K_M_, and *k*_cat_/K_M_). The independence of these parameters suggests that they can be separately engineered in a rational manner.

The Pearson correlation between linear T_m_ values and log-scale kinetic constants using SciPy was –0.27, –0.07, and –0.24 for each of *k*_cat_, K_M_, and *k*_cat_/K_M_, respectively. Fig 4 illustrates the correlations reported here as scatter plots. Individual mutants exhibiting function-stability trade-offs were identified and tallied. Mutants having a T_m_ greater than that of wild type and a kinetic constant less than that of wild type, and *vice versa*, of any magnitude, were counted as exhibiting a function-stability trade-off. By this analysis, the percentage of mutants in our data set that exhibit function-stability tradeoffs is 18.8% for *k*_cat_, 25.8% for K_M_, and 21.0% for *k*_cat_/K_M_. The statistical independence of stability and functional parameters in the BglB data set suggests that protein stability and enzyme kinetic constants can be separately designed in BglB.

### Relationship between sequence conservation and functional parameters of BglB mutants

**Fig 5:**
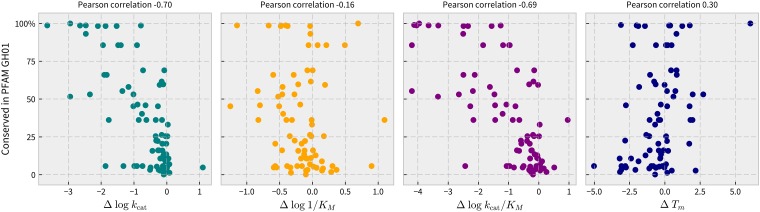
Correlations between conservation within functional protein family and enzyme functional parameters protein melting temperature (T_m_) and kinetic constants (*k*_cat_, K_M_, and *k*_cat_/K_M_) in the BglB system. Scatter plots showing conservation analysis from an alignment of 1,554 proteins in Pfam family 1 (glycoside hydrolases) versus measured values for T_m_ (linear scale, units of °C) and each of the kinetic constants *k*_cat_, K_M_, and *k*_cat_/K_M_ (log scale) with units of min^–1^, mM, and M^–1^min^–1^, respectively.

Correlation to conservation within the Pfam GH01 was assessed between each of T_m_, *k*_cat_, K_M_, and *k*_cat_/K_M_. The percent conservation was defined as the percentage of sequences in an alignment of 1,544 proteins from Pfam GH01 matching the BglB native residue in the alignment [10]. For the 129 variants reported here, the PCC between percent conservation and each of *k*_cat_, K_M_, *k*_cat_/K_M_, and T_m_ is found to be –0.70, –0.16, –0.69, and 0.30, respectively. Fig 5 illustrates the correlations as scatter plots with their associated PCC values.

### Assessment of current computational predictions of stability

**Fig 6:**
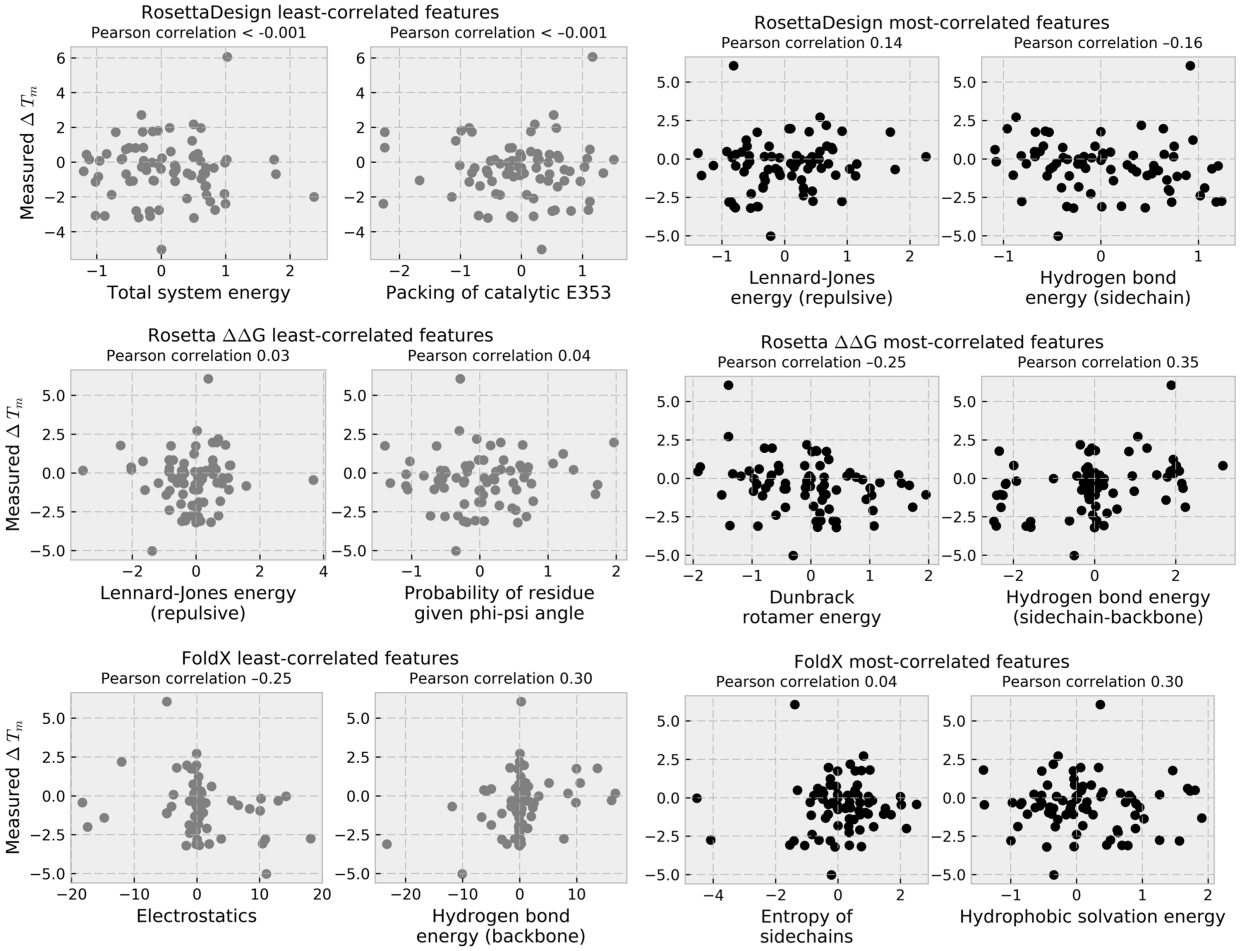
Correlations between experimentally-determined T_m_ and structural features from molecular modeling algorithms. For each of the three computational protocols used for prediction of stability in this study, the two most-correlated (black) and two least-correlated (gray) features are plotted against experimentally-determined T_m_. Pearson correlation between the two sets of values is provided above each plot. For descriptions of individual features for each of the three algorithms, see references for RosettaDesign [11], Rosetta ΔΔG [12], and FoldX [9].

Current algorithms for prediction of the stability effect of point mutations use published data sets to assess performance [12]. Thus, large, novel data sets such as the one presented here present an unbiased evaluation of algorithm performance. The correlation between functional T_m_ and calculated features from current algorithms designed to predict protein stability was assessed by Pearson correlation (for a list of all features for which Pearson correlation was assessed, see S2 Table). In Fig 6, the two most-correlated and the two least-correlated features for RosettaDesign, Rosetta ΔΔG, and FoldX are illustrated. This assessment is “blind” in the sense that it is based on a novel data set of mutations not previously used to train current protein modeling algorithms. One caveat is that many of the mutations were selected using the Foldit interface to Rosetta, and are predicted to be no worse than five Rosetta Energy Units than the native. Therefore, this represents a set of mutations mostly predicted to be compatible with the model of the BglB-pNPG complex in the Rosetta forcefield. Another caveat is that current algorithms are largely trained on T_m_ values derived from direct physical measurements of protein unfolding. The functional T_m_ used in this study is an indirect measurement of protein unfolding, and is derived from the amount of functionally folded protein remaining in solution after heat challenge. The degree of correlation between a functional and thermodynamic T_m_ measurement, which is currently unknown, may affect the predicative ability of algorithms.

For each of RosettaDesign, Rosetta ΔΔG, and FoldX, the correlation between functional T_m_ and the metric associated with total system energy was –0.0006, –0.16, and –0.18, respectively. For RosettaDesign, the feature that was most strongly correlated to functional T_m_ was the hydrogen bond energy of protein sidechains [11] (Pearson correlation –0.16). For Rosetta ΔΔG, the feature from the Rosetta score function that was found to be most strongly correlated with functional T_m_ was the energy of H-bonds between backbone and side chain atoms (Pearson correlation 0.35). For FoldX, H-bond energy between backbone atoms [9] and functional T_m_ had a Pearson correlation of 0.30. The weak correlation between experimental data and predictions reveal that the underlying algorithms require improvement in order to robustly predict stability of enzyme mutants.

## Discussion

It is widely hypothesized that enzymes must balance thermodynamic stability with functional properties, and that there are explicit trade-offs between these properties [2,3]. This is supported by previous studies for a variety of enzymes, including ribonuclease (“Barnase”) [21], T4 lysozyme [22], and β-lactamase [23] that show tradeoffs between protein stability and functional parameters such as *k*_cat_/K_M_. The data set reported here reveals that, for BglB, it is not generally true that individual residue identities are trade-offs between function and stability. Pearson correlations between functional thermal stability and parameters *k*_cat_, K_M_, and *k*_cat_/K_M_ in the BglB data set are < 0.3.

For systems such as BglB, in which kinetics and thermal stability are independent biophysical properties, engineering efforts can avoid the multi-objective optimization problems associated with maximizing two parameters (such as *k*_cat_ and thermal stability) simultaneously [24,25]. However, there is an established relationship between the stability of functional proteins and their ability to gain new functions through evolution [26]. In the context of living organisms, it is possible that the independence of thermal stability and functional parameters in enzymes such as glycosyl hydrolases leads to greater evolvability of new functions. This is exemplified by mutations that enhance thermal stability while remaining neutral in regard to the protein's natural function, as these mutations, from an evolutionary perspective, could increase tolerance to subsequent mutations that trade stability for features such as pre-ordered active sites that give rise to new functions [27].

We found an inverse correlation relating protein function (*k*_cat_ and *k*_cat_/K_M_) to conservation within Pfam GH01. This is consistent with the discovery that negative selection purges natural functional proteins of destabilizing mutations [28]. Our finding is also in agreement with a previous study of over 1 million systematically mapped variants of Bgl3, a homolog of BglB, that found strong inverse correlation between mutational tolerance and conservation when assaying for enzyme activity [6]. Neither K_M_ nor functional T_m_ appeared to have a strong relationship with sequence conservation. This has significant implications for the field of enzyme engineering, as conservation is commonly used to guide mutagenesis efforts [29].

Comparison of computational predictions of protein stability with experimental measurements reveal only a very weak correlation (PCC < 0.4) between the single most correlated feature and observed functional T_m_ for the BglB system. Furthermore, the most strongly-correlated feature to T_m_ was not found to be the total system energy for any of the three modeling protocols tested. While current algorithms perform well on some data sets, they are not robust predictors for every protein system of interest. This highlights the complexity of protein sequence-structure-function space and the need to continue expansion of data sets for training protein modeling algorithms. In addition to “brute force” methods, such as the one presented here, exciting advances in mapping protein sequence-functional space using experimental techniques such as high-throughput assays, microfluidics, and deep sequencing [6,30,31] have the potential to generate the transformative data sets need to develop a new generation of data-driven protein design algorithms.

## Supporting information

S1 Table Table of protein expression (0 = no, 1 = yes), functional melting temperature (°C), kinetic constants *k*_cat_ (min^-1^), K_M_ (mM) and *k*_cat_/K_M_ (M^-1^min^-1^), and statistical analysis (1 standard deviation error, in the same units as for the value) for BglB and each of 129 mutants. (CSV)

S2 Table Table of single feature correlations between the computational algorithms and experimentally-determined T_m_ values. (CSV)

S1 Text Additional information about experimental procedures. (DOCX)

S1 Figs Images of SDS-PAGE analysis for 129 mutants and batched replicates of the BglB WT protein (ZIP file containing JPEG images). (ZIP)

S2 Figs Michaelis-Menten plots for each mutant for which kinetic constants are reported for the first time (ZIP file containing PNG images). (ZIP)

S3 Figs Plot of protein melting curve for each mutant for which T_m_ is reported (ZIP file containing PNG images). (ZIP)

S1 Code Details of computational protocols (ZIP archives containing text files). (ZIP)
