# Supplementary material for: Thermal stability and kinetic constants for 129 variants of a family 1 glycoside hydrolase reveal that enzyme activity and stability can be separately designed
Source: PLoS One. 2017 May 22;12(5):e0176255. doi: 10.1371/journal.pone.0176255 (PMC5439667; doi:10.1371/journal.pone.0176255)
Supplement: S1 Text — (DOCX) [file pone.0176255.s003.docx]

**S1 Text: Supplemental materials and methods**

Mutants were designed using Foldit, a graphical user interface to the Rosetta Molecular Modeling Suite. Mutants were chosen based on proximity to the active site as well as Foldit’s predicted energy. Mutations within 12 Å of the active site, and those that did not increase the total system energy by more than 5 Rosetta energy units, were selected for experimental characterization. No other limitations were placed on designed mutations.

        A sequence coding for BglB (Uniprot P22505) was codon-optimized for *Escherichia coli* and manufactured as a DNA String by Life Technologies. Using Gibson assembly, gene was inserted between the NdeI and XhoI sites of pET29b, adding a C-terminal His tag onto the protein sequence. Kunkel mutagenesis was used to create site-specific mutations, and all plasmids were sequence-verified.

For protein production, 20 μL of chemically-competent *Escherichia coli* BL21(DE3) (Novagen) were transformed on ice with 1 μL of plasmid in buffer at a concentration of 90 to 130 ng/μL. The competent cell-plasmid mixture was temperature shocked to induce plasmid uptake by heating at 42°C for one minute and then chilling on ice for one minute. Cells were recovered in 200 μL Terrific Broth (TB) media at 37 °C for one hour. They were then plated onto an LB agarose plate containing 50 mg/mL kanamycin, and incubated for 24 hours at 37 °C.

        For each mutant, a 50 mL Falcon tube containing 5 mL TB with 50 mg/mL kanamycin was inoculated with one colony from a kanamycin selection plate. Tubes were covered with breathable seals and incubated with shaking for 24 hours at 37 °C.

        Growth cultures were pelleted by centrifugation at 4700 RPM for 10 minutes and the supernatant was discarded. The cell pellet was resuspended in 5 mL of induction medium (TB with 1 mM isopropyl-β-D-thiogalactopyranoside and 50 mg/mL kanamycin). The tubes were covered again with breathable seals and incubated with shaking at 18 °C for 24 hours.

        The 5 mL expression culture was pelleted by centrifugation at 4700 RPM for 10 minutes and the supernatant was discarded. The resulting pellet was suspended in 500 μL wash buffer (50 mM HEPES, 150 mM sodium chloride, 15 mM imidazole, pH 7.50) and lysed with BugBuster protein extraction reagent (Millipore) and 1 mg lysozyme, 0.1 mg DNase, and 0.1 mg phenylmethylsulfonyl fluoride per sample.

After 20 min, lysate was centrifuged at 14,700 RPM for ten minutes. The supernatant was loaded onto protein purification columns (BioSpin 732-6008) prepared with 100 μL of 50% Ni-NTA resin slurry. After equilibration with 500 μL wash buffer, two 500 μL aliquots of supernatant were added to the columns. Six rounds of 500 μL wash buffer were then allowed to drip through the columns. Resulting protein micro-columns were then transferred to 2 mL tubes for elution. Protein was eluted in 2x100 μL elution buffer (50 mM HEPES, 150 mM sodium chloride, and 25 mM EDTA, pH 7.50). A brief centrifugation at 4000 RPM ensured all protein was collected.

Protein yield was then determined via ratio of absorbance at 260 and 280 nm and SDS-PAGE.

        Diluted protein solution of each enzyme variant was dispensed in 50 μl aliquots into a 96-well PCR (Fisherbrand nonskirted), which was then placed in a thermocycler (BioRad T100 Thermocycler) and incubated for 30 minutes with a gradient from 30C-50C.  Separately, in another plate, 75 μl of diluted substrate solution was aliquoted into each well of a 96-well nonbinding assay plate (Corning Costar #3884).  The assay was initiated by multi-channel pipetting 25 μl of each enzyme variant to its corresponding row of the 96- well nonbinding assay plate.  Absorbance was then measured at 420 nm every minute for 45 minutes to determine the rate of the reaction.

Unless otherwise noted, all supplies were purchased from Sigma-Aldrich.
