# Supplementary figures and images for: Thermal stability and kinetic constants for 129 variants of a family 1 glycoside hydrolase reveal that enzyme activity and stability can be separately designed (part 1 of 2)
Source: PLoS One. 2017 May 22;12(5):e0176255. doi: 10.1371/journal.pone.0176255 (PMC5439667; doi:10.1371/journal.pone.0176255)

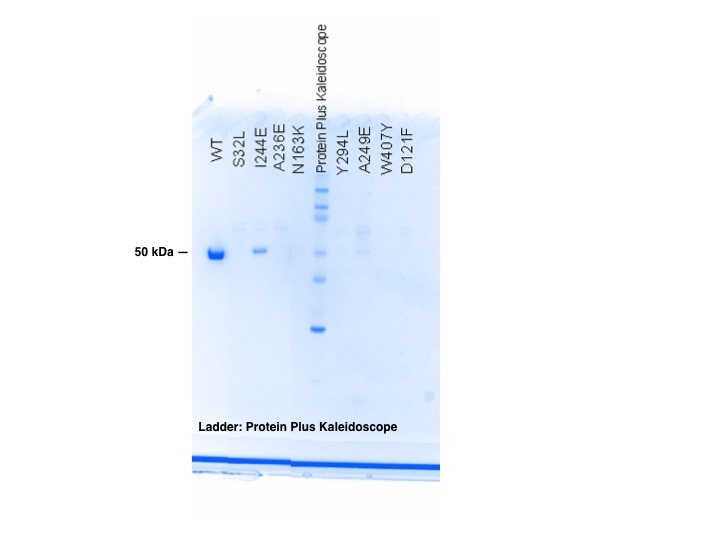

Supplement: S1 Figs — (ZIP) [file pone.0176255.s004.zip › S1 Figures/Gel 1.jpg]

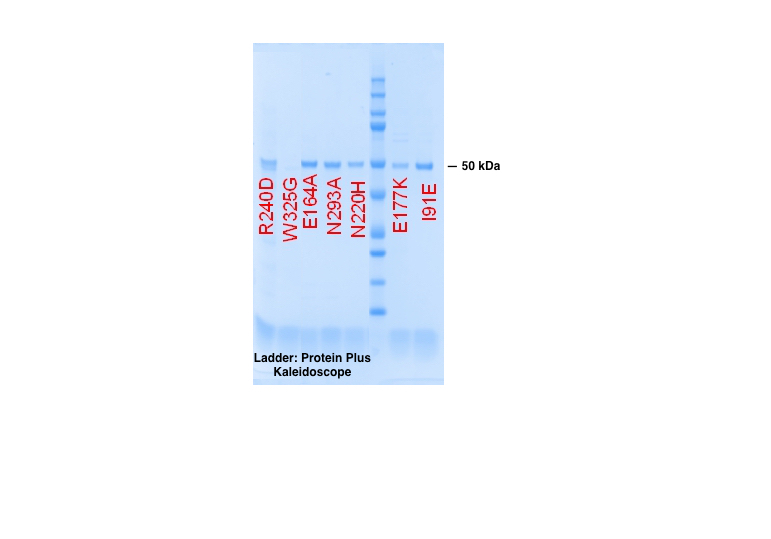

Supplement: S1 Figs — (ZIP) [file pone.0176255.s004.zip › S1 Figures/Gel 10.jpg]

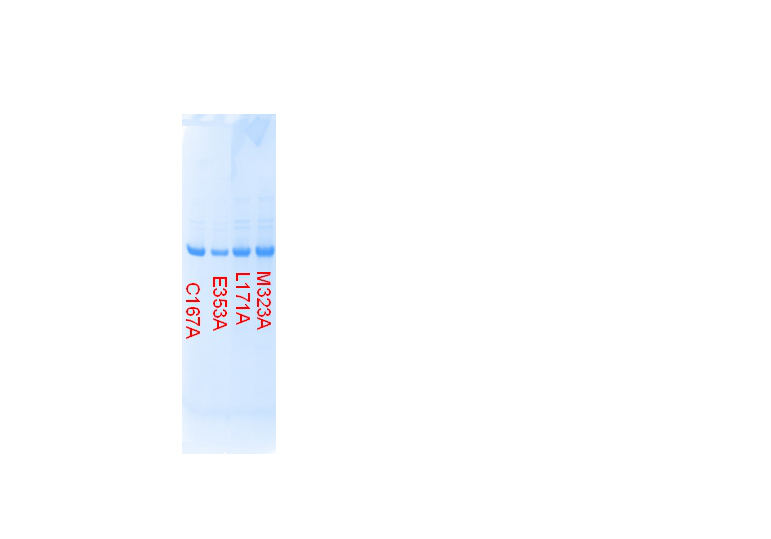

Supplement: S1 Figs — (ZIP) [file pone.0176255.s004.zip › S1 Figures/Gel 11.jpg]

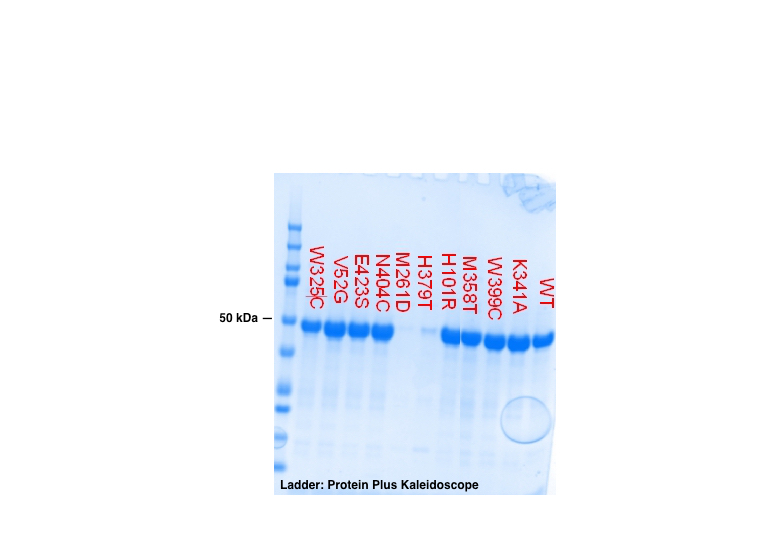

Supplement: S1 Figs — (ZIP) [file pone.0176255.s004.zip › S1 Figures/Gel 12.jpg]

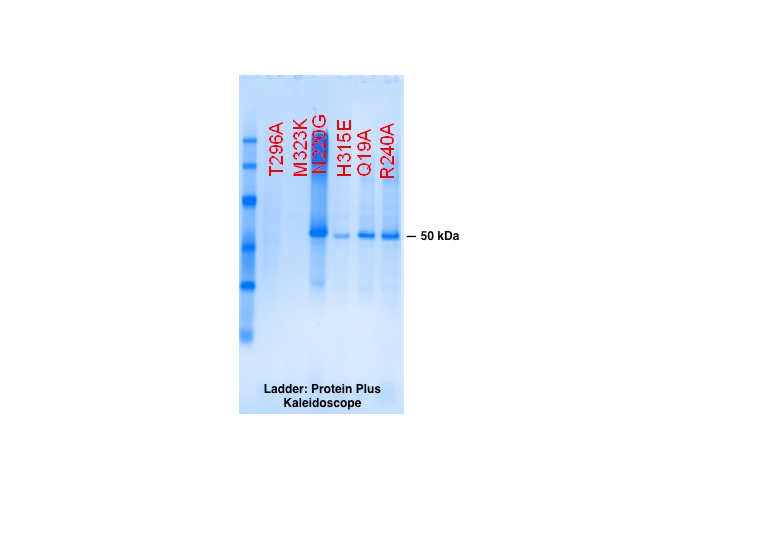

Supplement: S1 Figs — (ZIP) [file pone.0176255.s004.zip › S1 Figures/Gel 13.jpg]

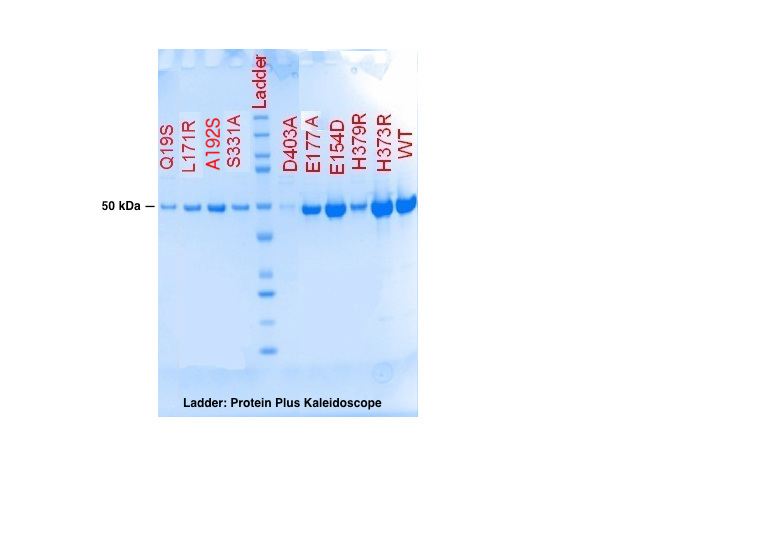

Supplement: S1 Figs — (ZIP) [file pone.0176255.s004.zip › S1 Figures/Gel 14.jpg]

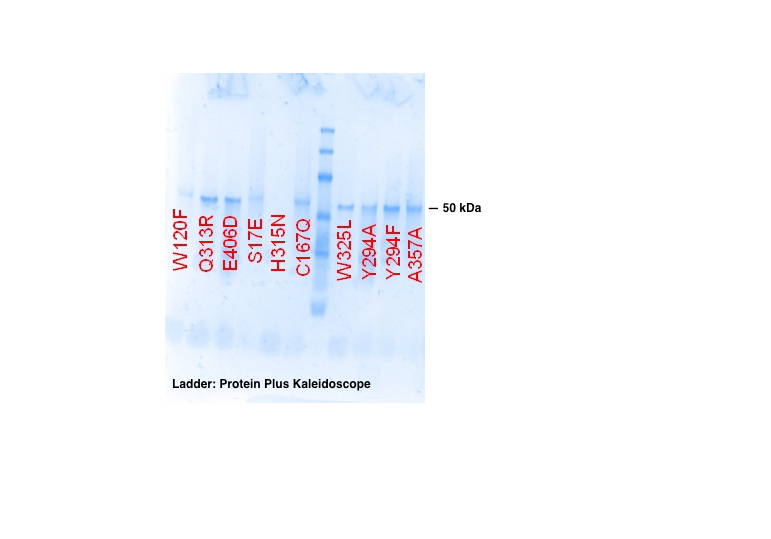

Supplement: S1 Figs — (ZIP) [file pone.0176255.s004.zip › S1 Figures/Gel 15.jpg]

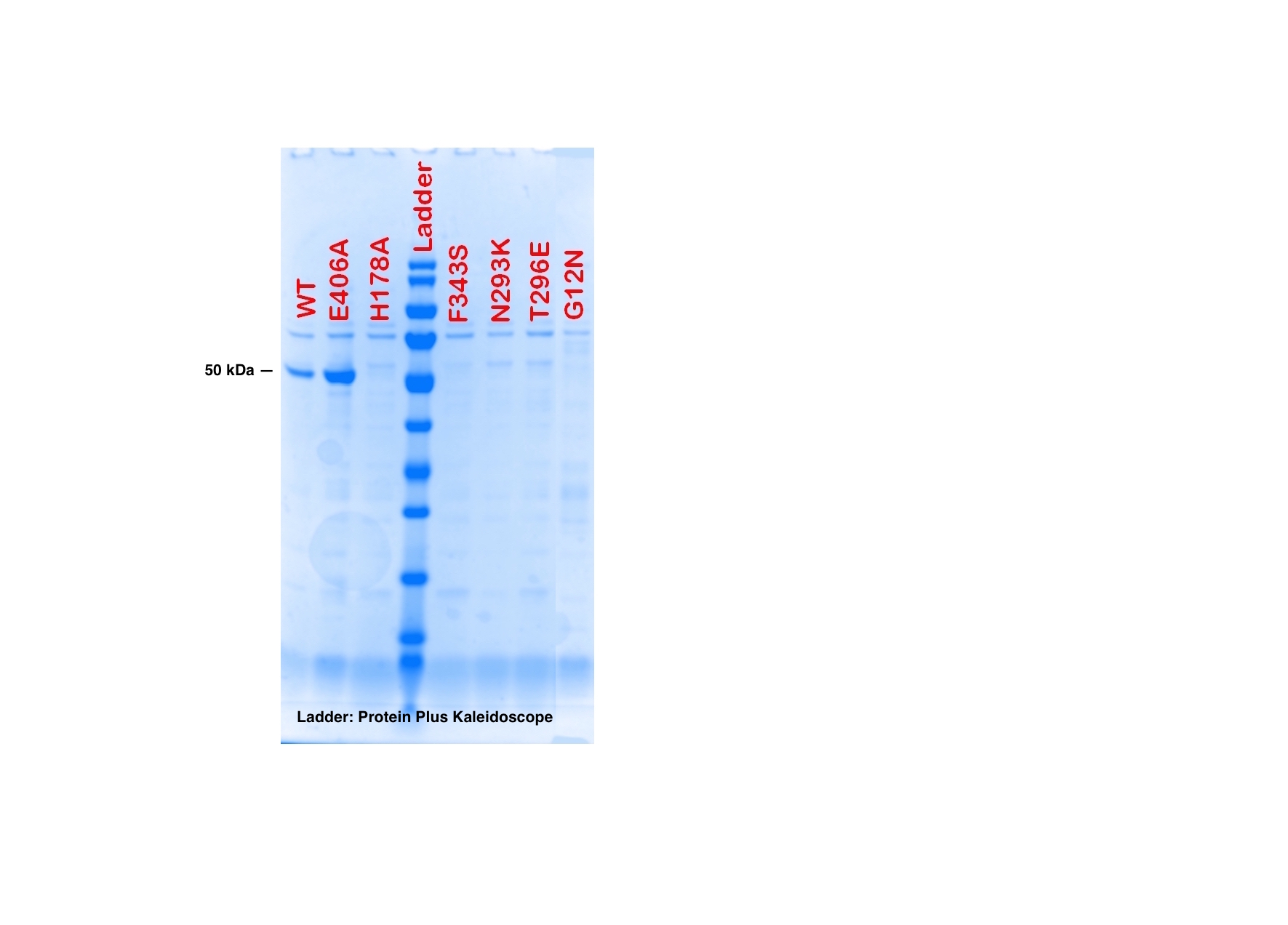

Supplement: S1 Figs — (ZIP) [file pone.0176255.s004.zip › S1 Figures/Gel 16.jpg]

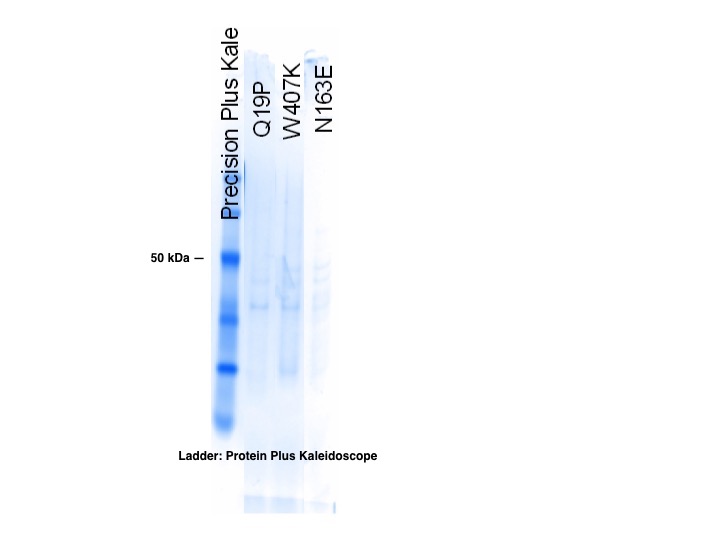

Supplement: S1 Figs — (ZIP) [file pone.0176255.s004.zip › S1 Figures/Gel 17.jpg]

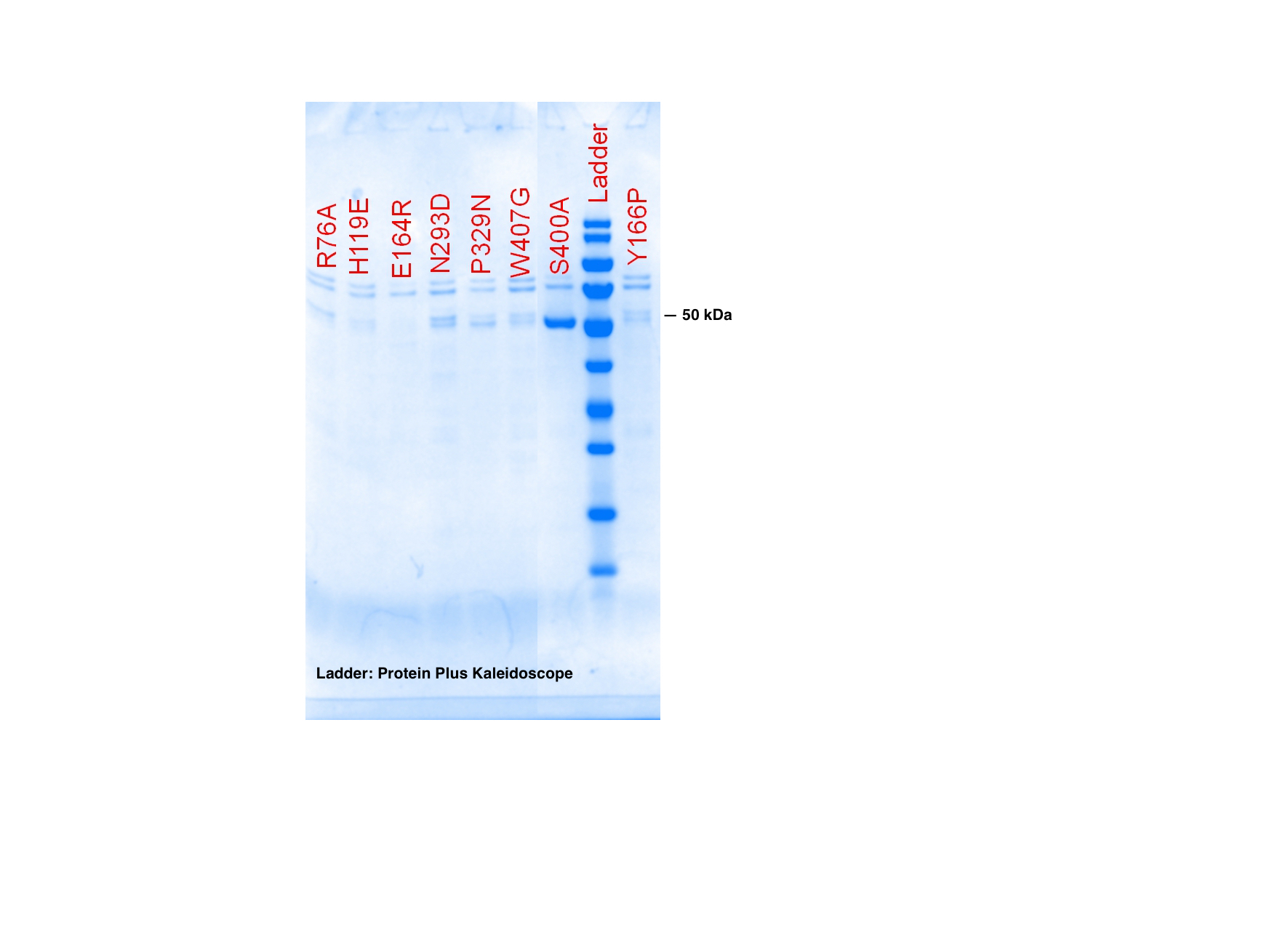

Supplement: S1 Figs — (ZIP) [file pone.0176255.s004.zip › S1 Figures/Gel 18.jpg]

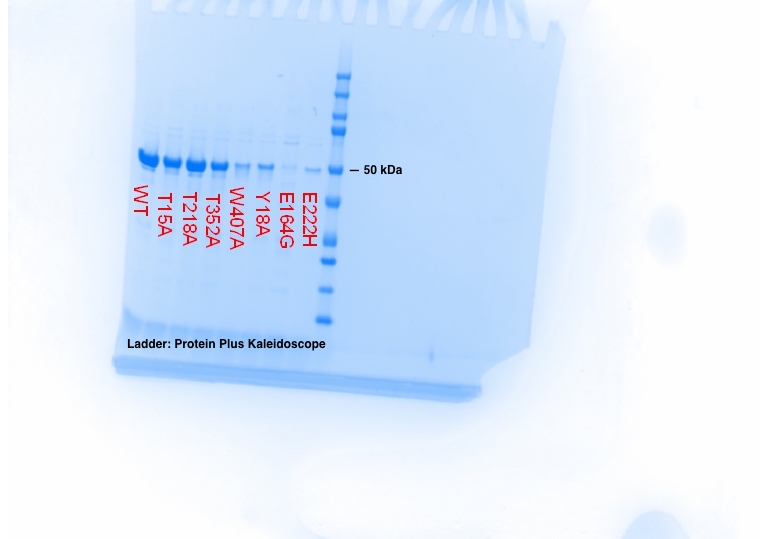

Supplement: S1 Figs — (ZIP) [file pone.0176255.s004.zip › S1 Figures/Gel 2.jpg]

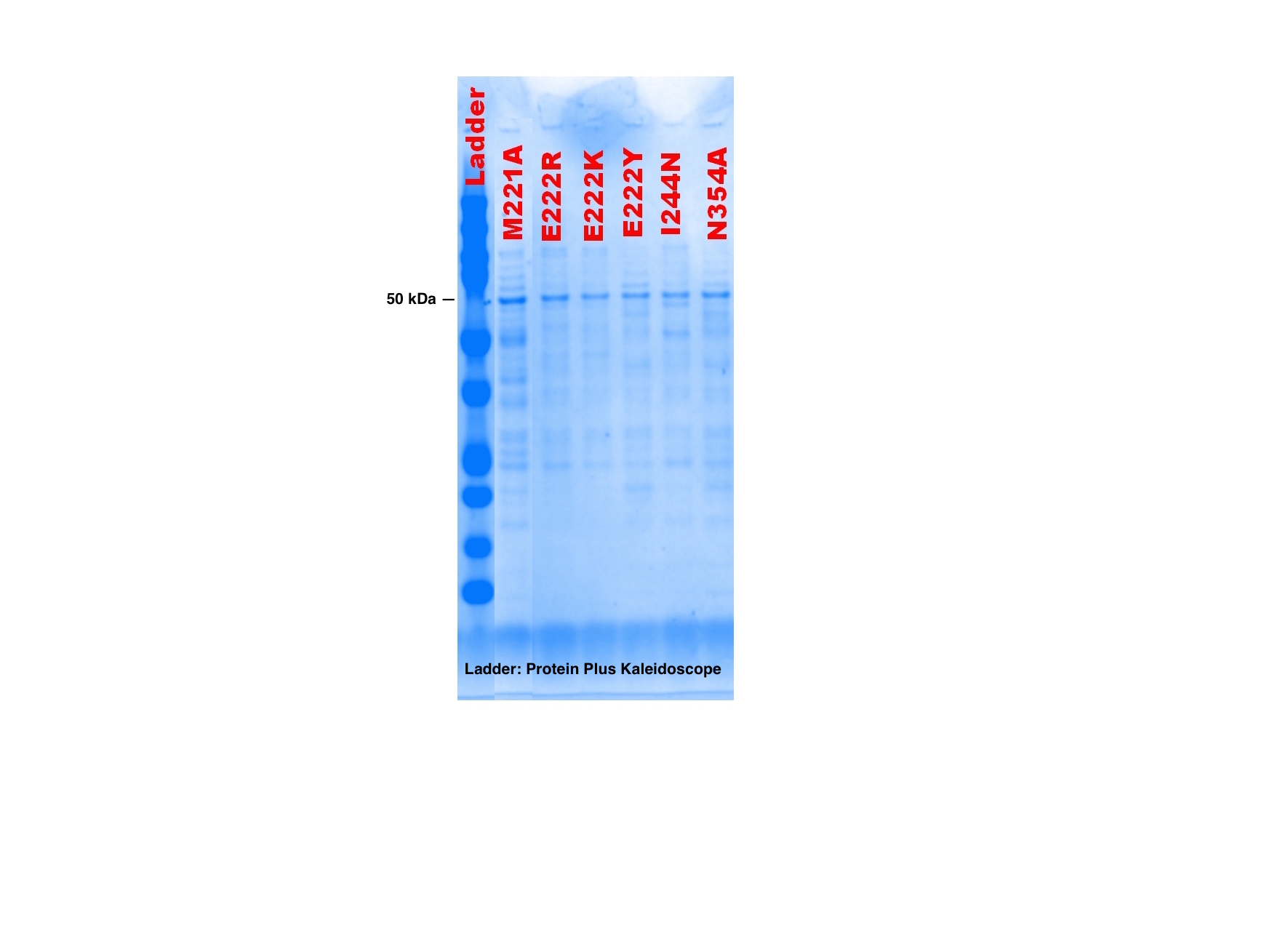

Supplement: S1 Figs — (ZIP) [file pone.0176255.s004.zip › S1 Figures/Gel 20.jpg]

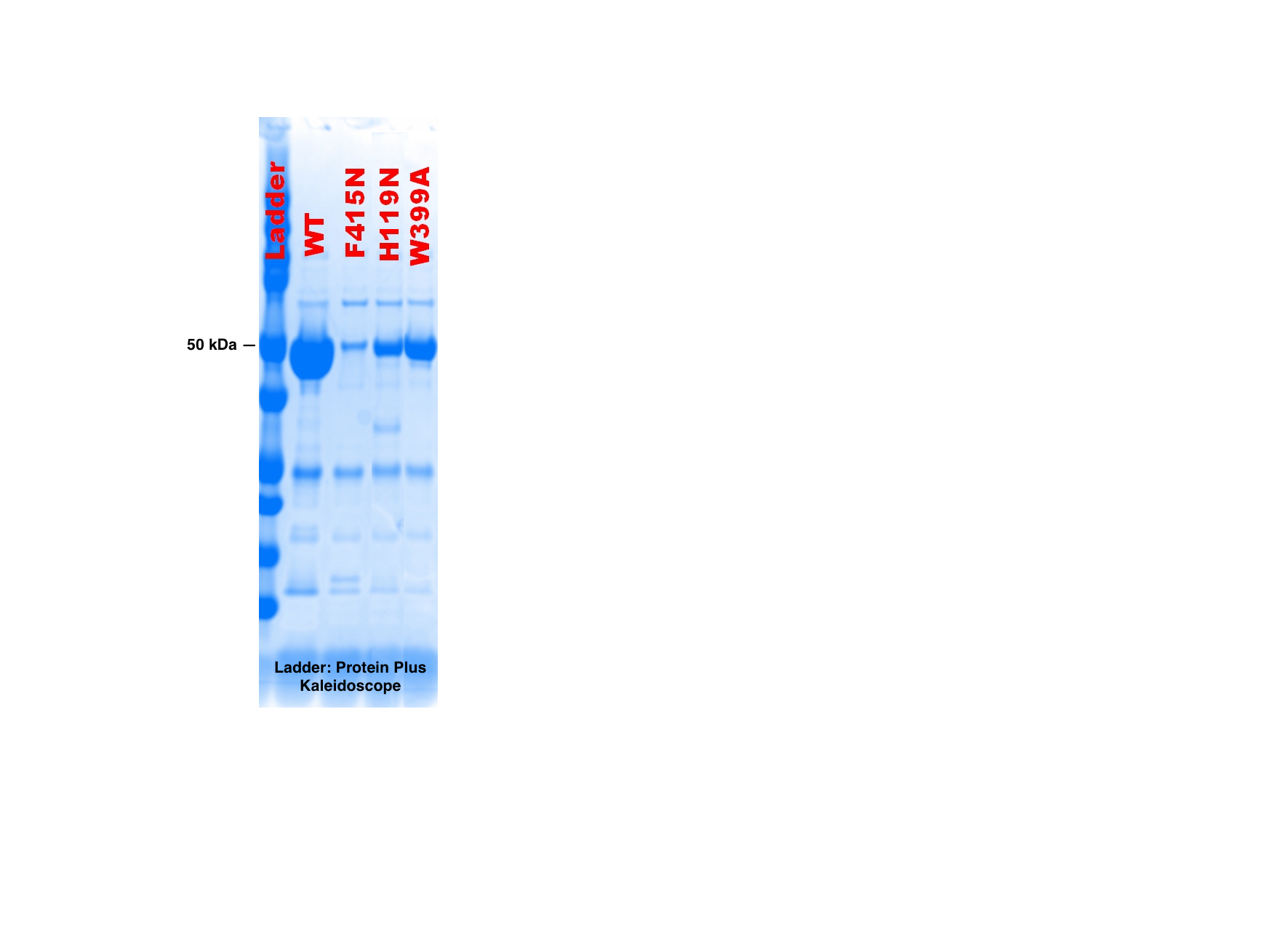

Supplement: S1 Figs — (ZIP) [file pone.0176255.s004.zip › S1 Figures/Gel 21.jpg]

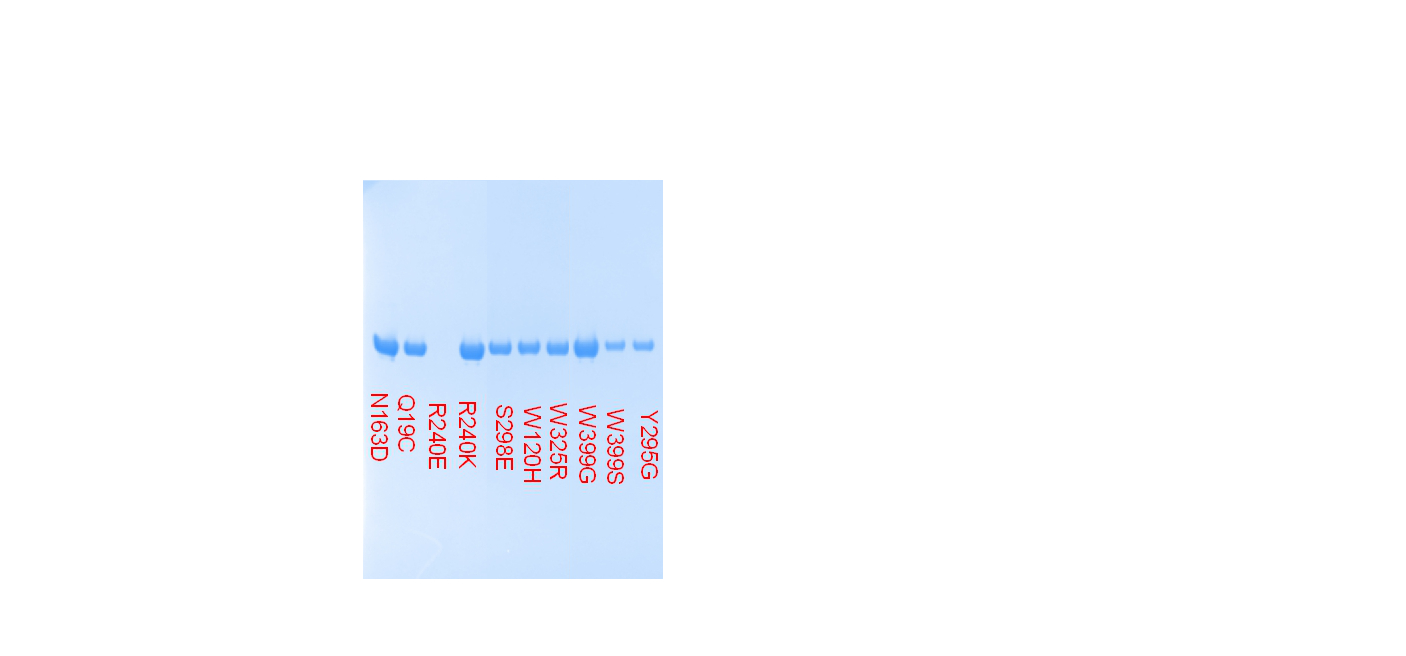

Supplement: S1 Figs — (ZIP) [file pone.0176255.s004.zip › S1 Figures/Gel 3.jpg]

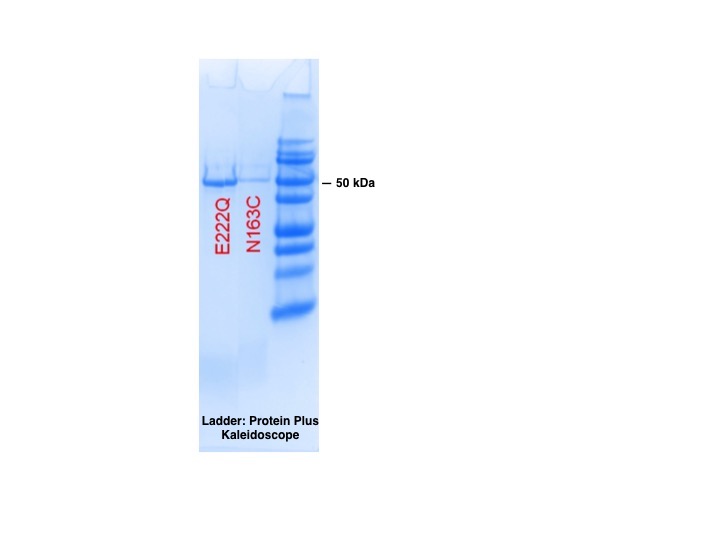

Supplement: S1 Figs — (ZIP) [file pone.0176255.s004.zip › S1 Figures/Gel 4.jpg]

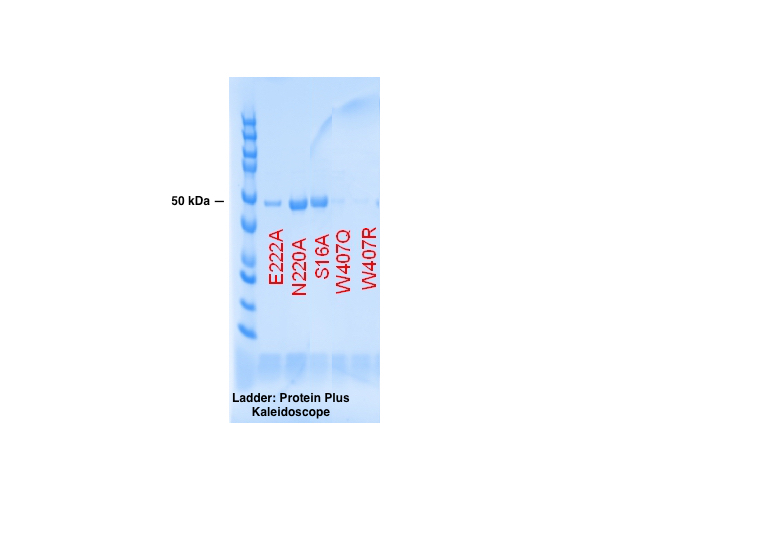

Supplement: S1 Figs — (ZIP) [file pone.0176255.s004.zip › S1 Figures/Gel 5.jpg]

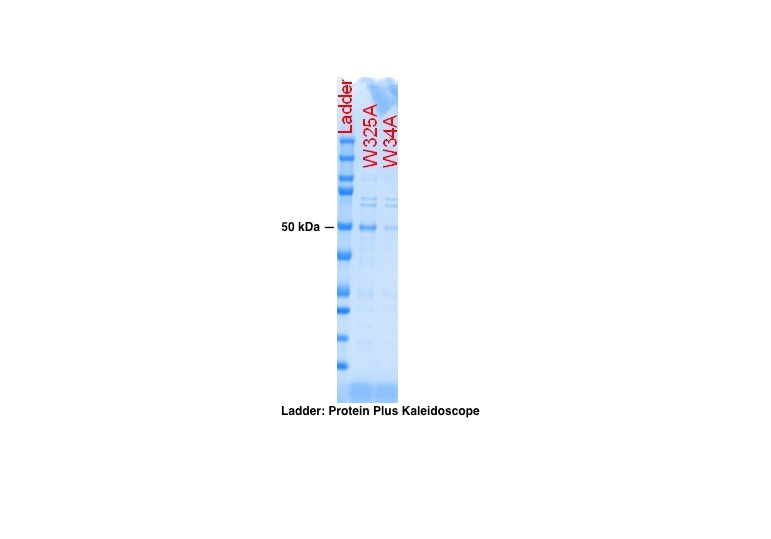

Supplement: S1 Figs — (ZIP) [file pone.0176255.s004.zip › S1 Figures/Gel 6.jpg]

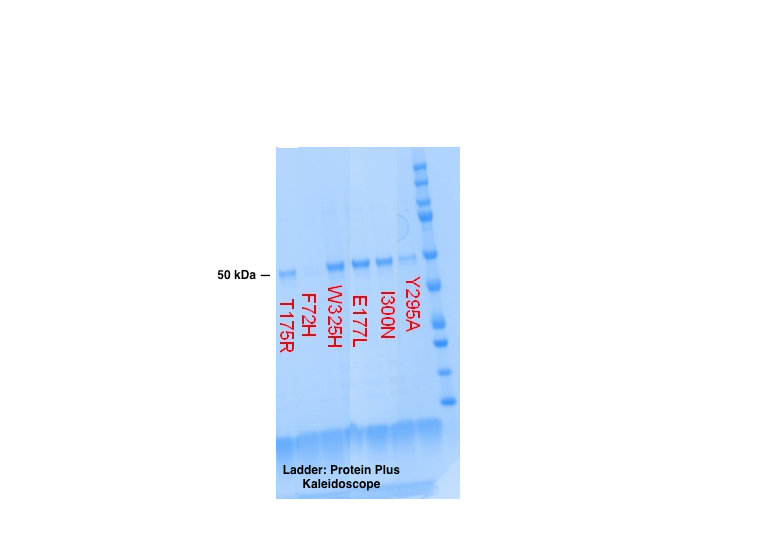

Supplement: S1 Figs — (ZIP) [file pone.0176255.s004.zip › S1 Figures/Gel 7.jpg]

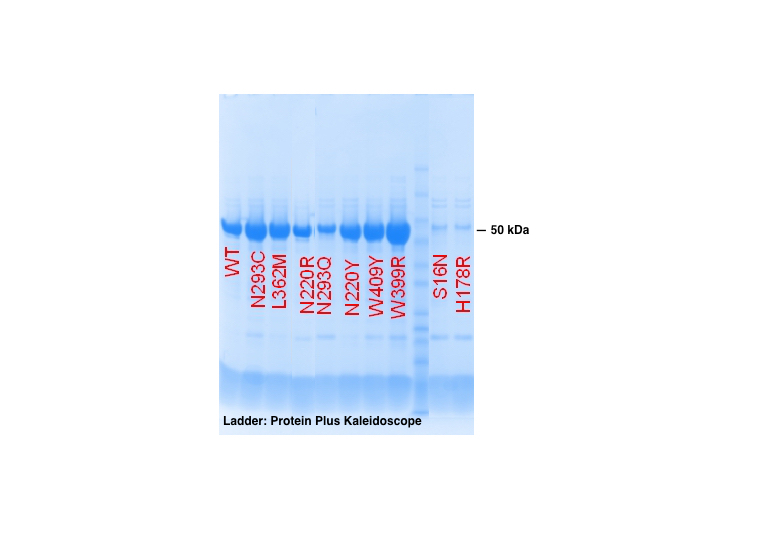

Supplement: S1 Figs — (ZIP) [file pone.0176255.s004.zip › S1 Figures/Gel 8.jpg]

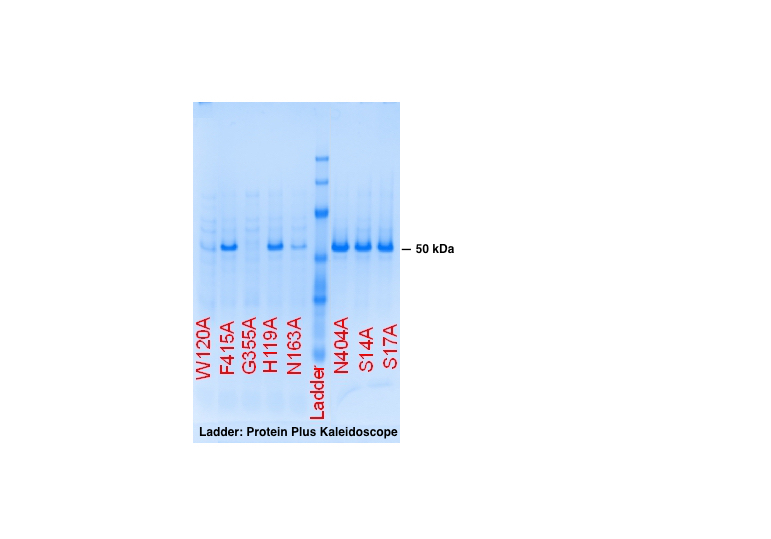

Supplement: S1 Figs — (ZIP) [file pone.0176255.s004.zip › S1 Figures/Gel 9.jpg]

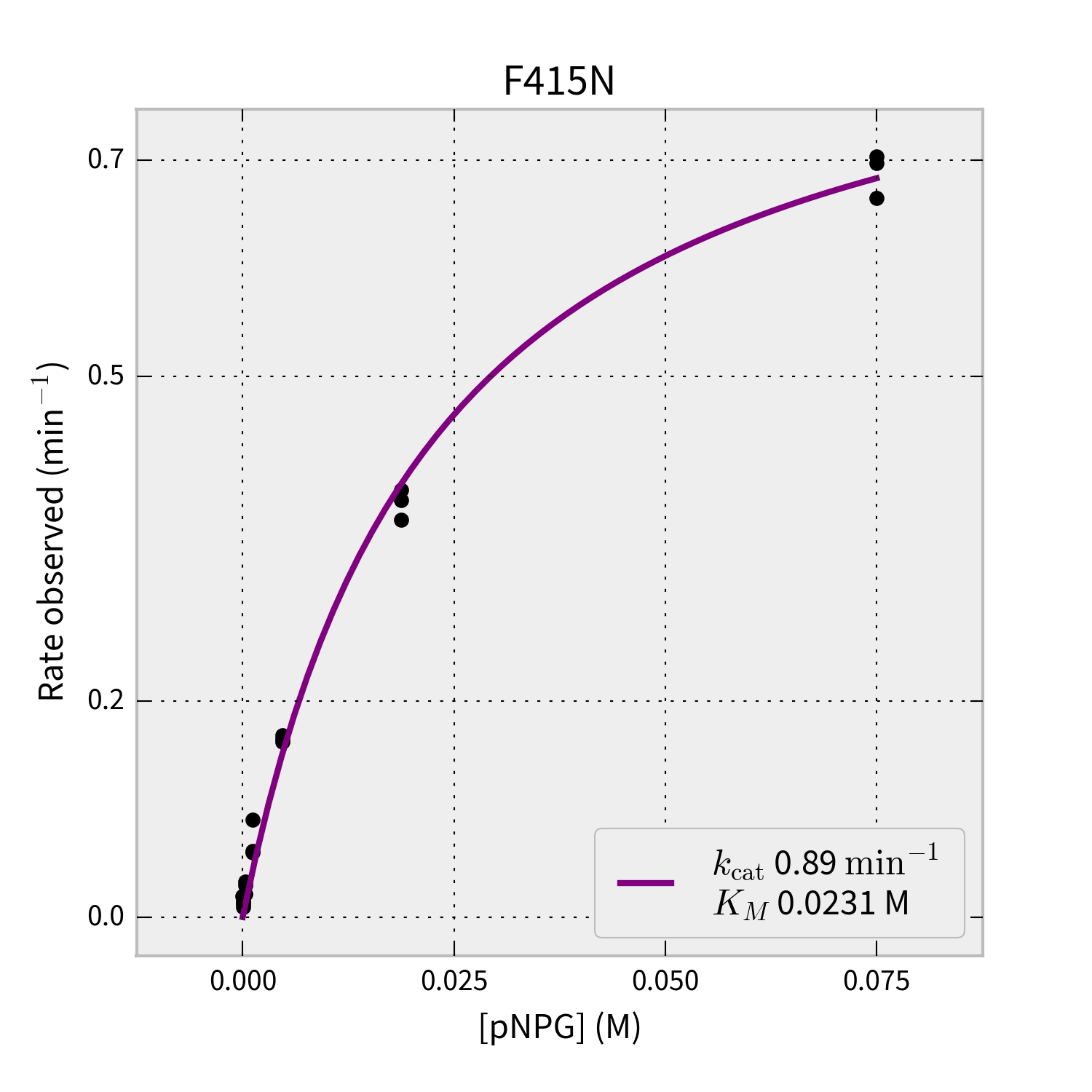

Supplement: S2 Figs — (ZIP) [file pone.0176255.s005.zip › S2 Figures/F415N.png]

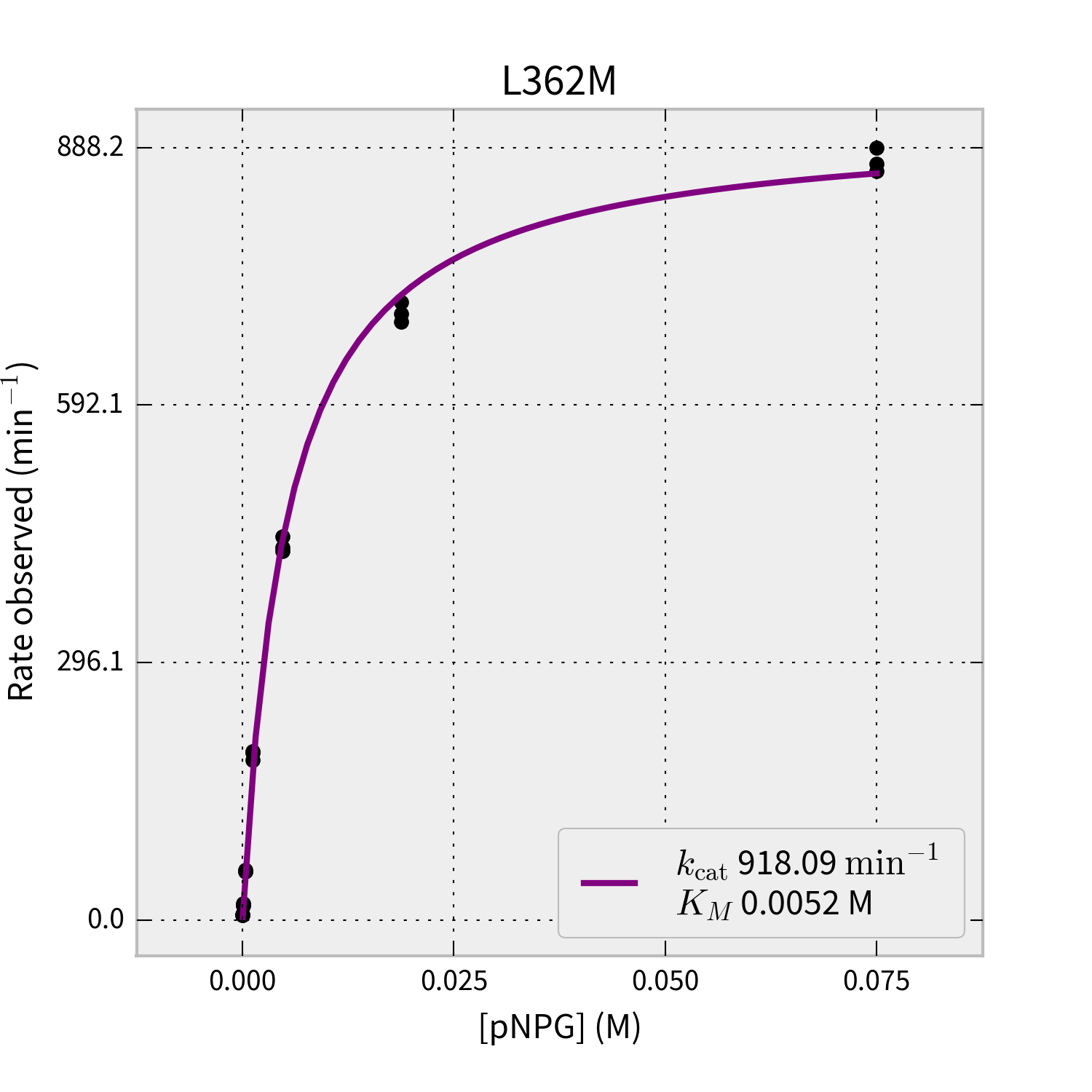

Supplement: S2 Figs — (ZIP) [file pone.0176255.s005.zip › S2 Figures/L362M.png]

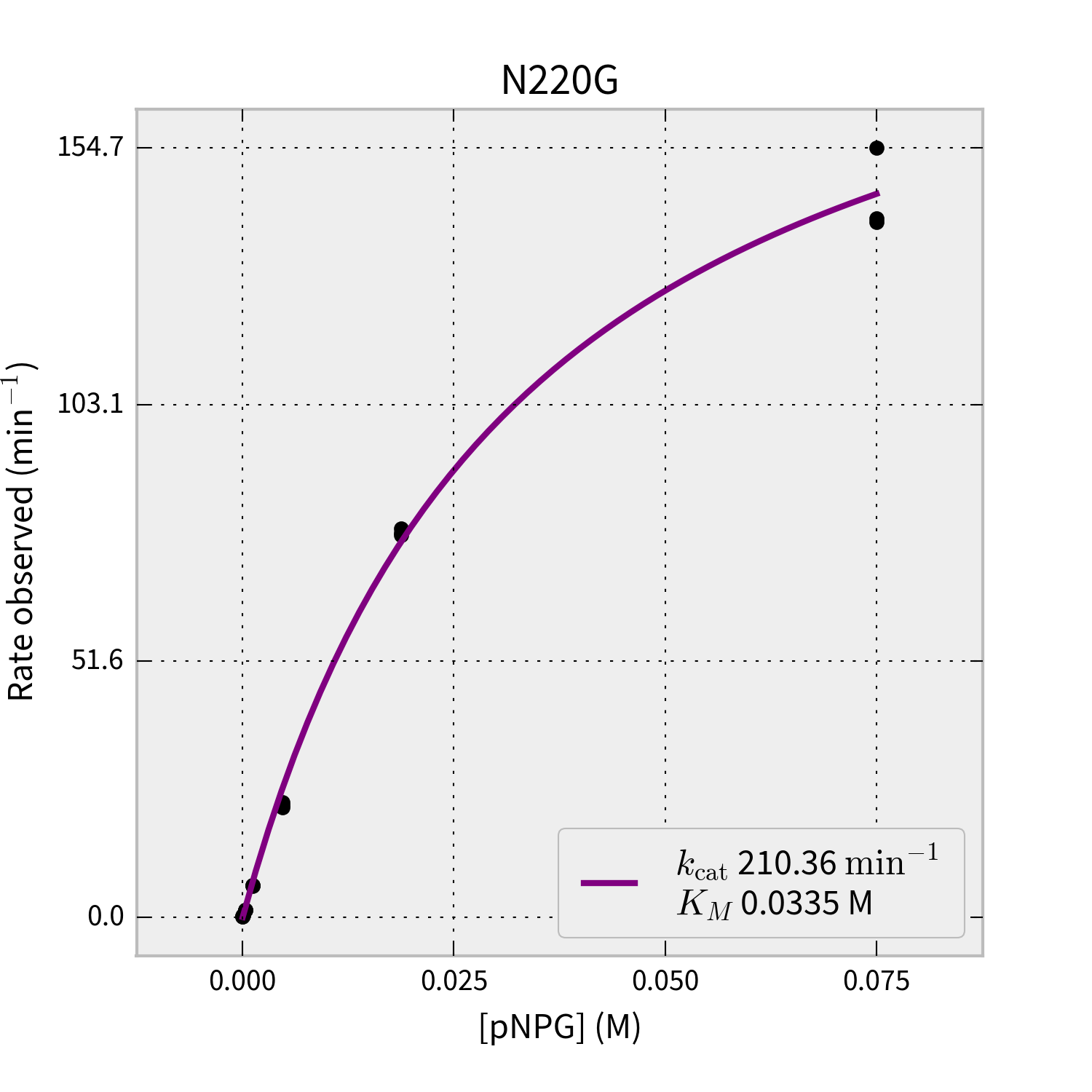

Supplement: S2 Figs — (ZIP) [file pone.0176255.s005.zip › S2 Figures/N220G.png]

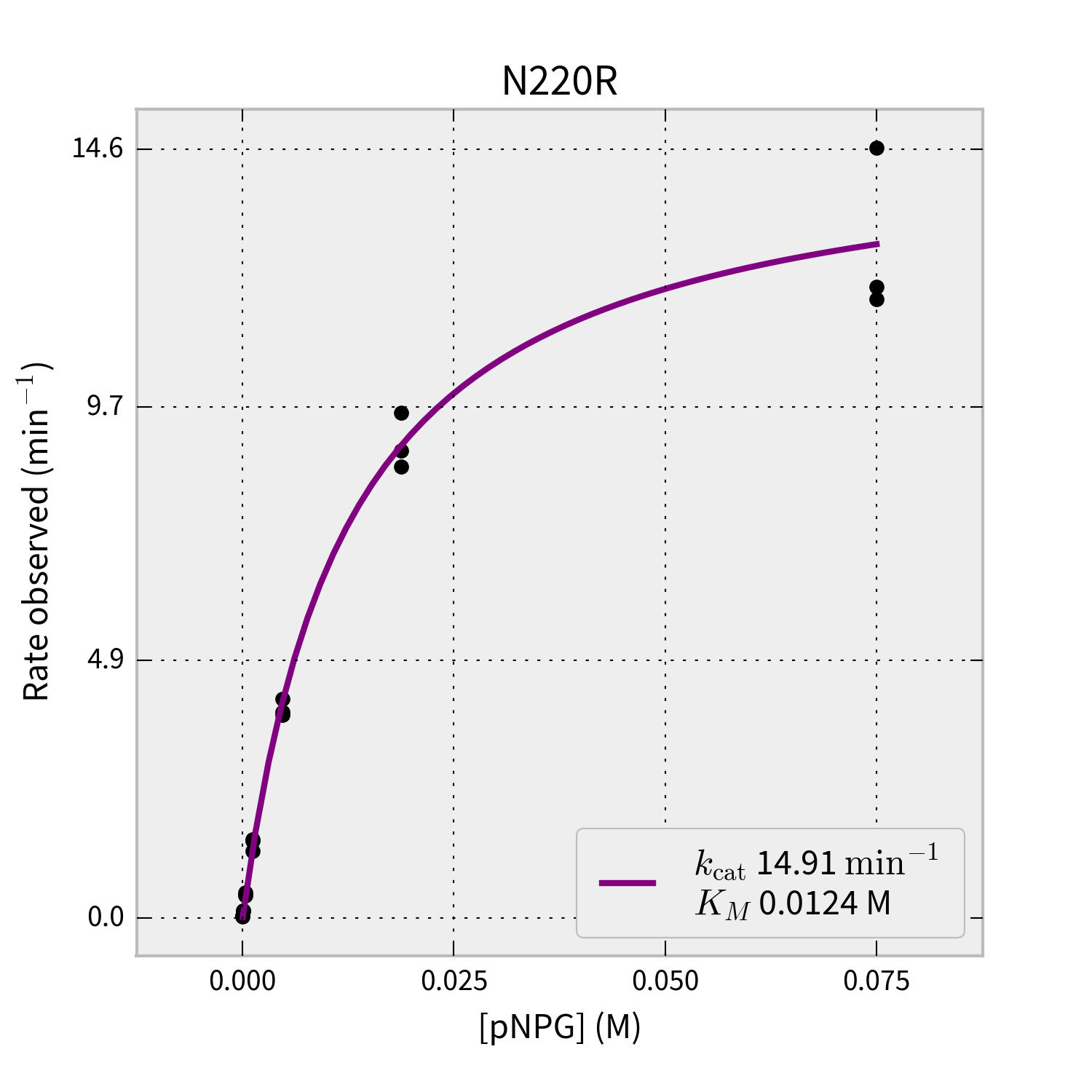

Supplement: S2 Figs — (ZIP) [file pone.0176255.s005.zip › S2 Figures/N220R.png]

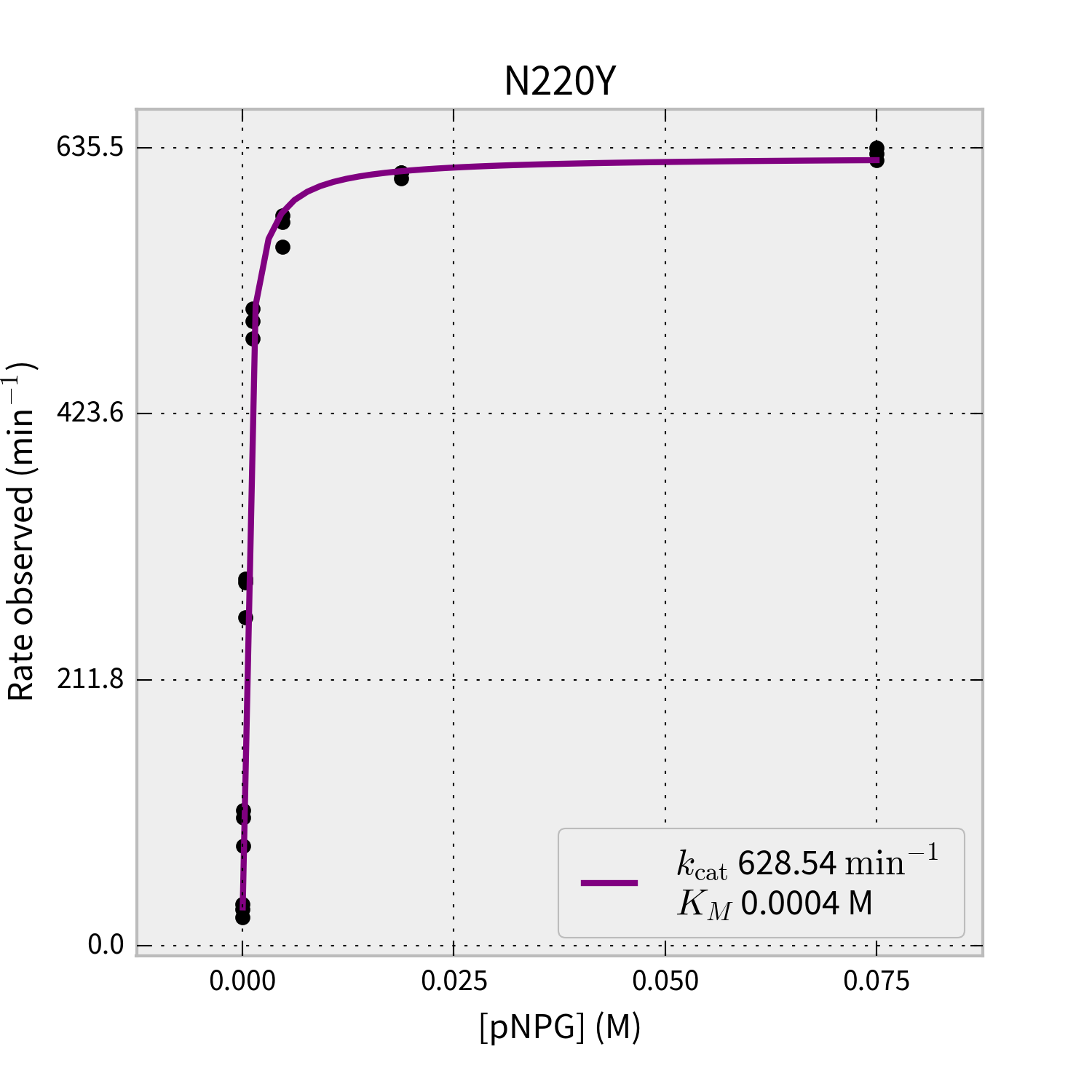

Supplement: S2 Figs — (ZIP) [file pone.0176255.s005.zip › S2 Figures/N220Y.png]

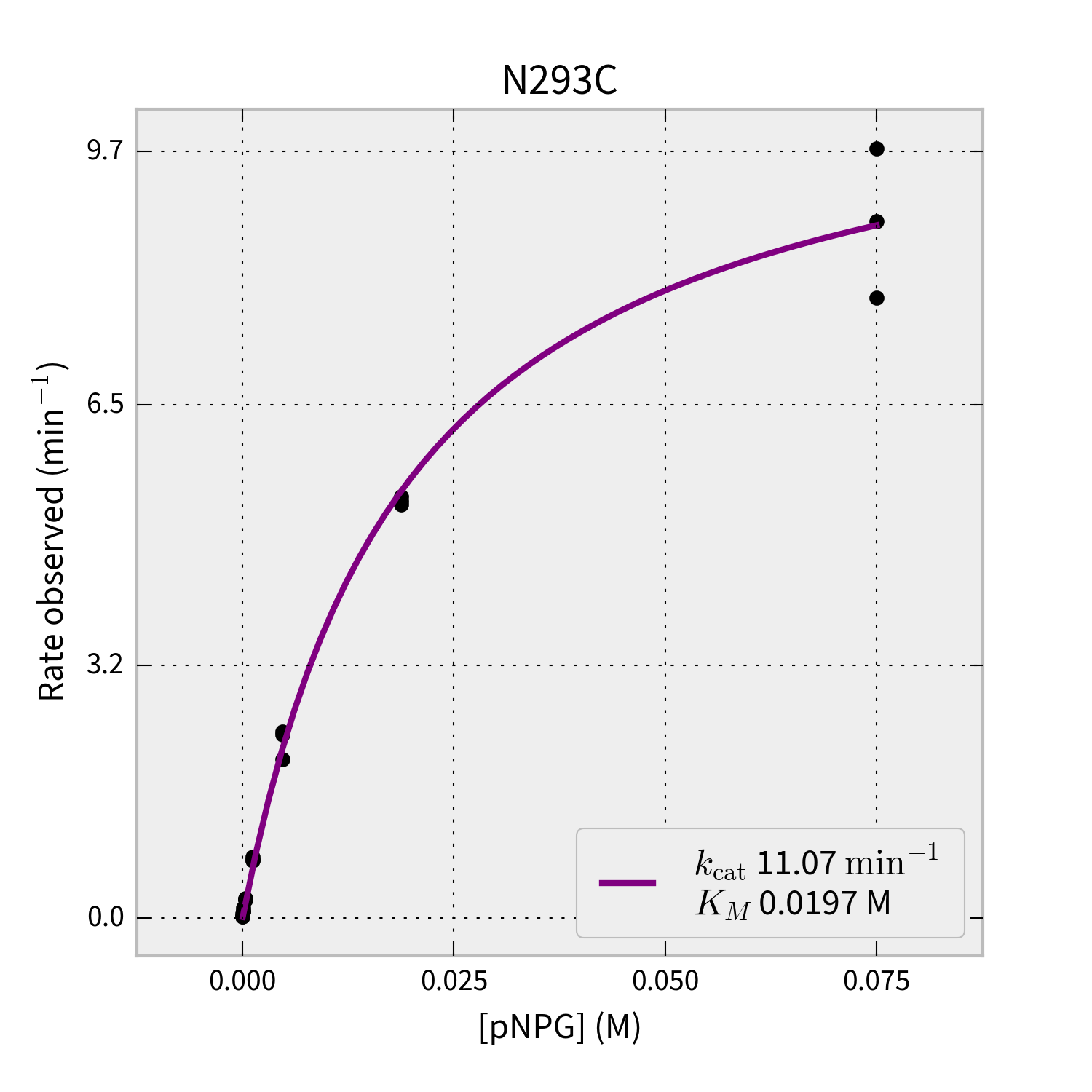

Supplement: S2 Figs — (ZIP) [file pone.0176255.s005.zip › S2 Figures/N293C.png]

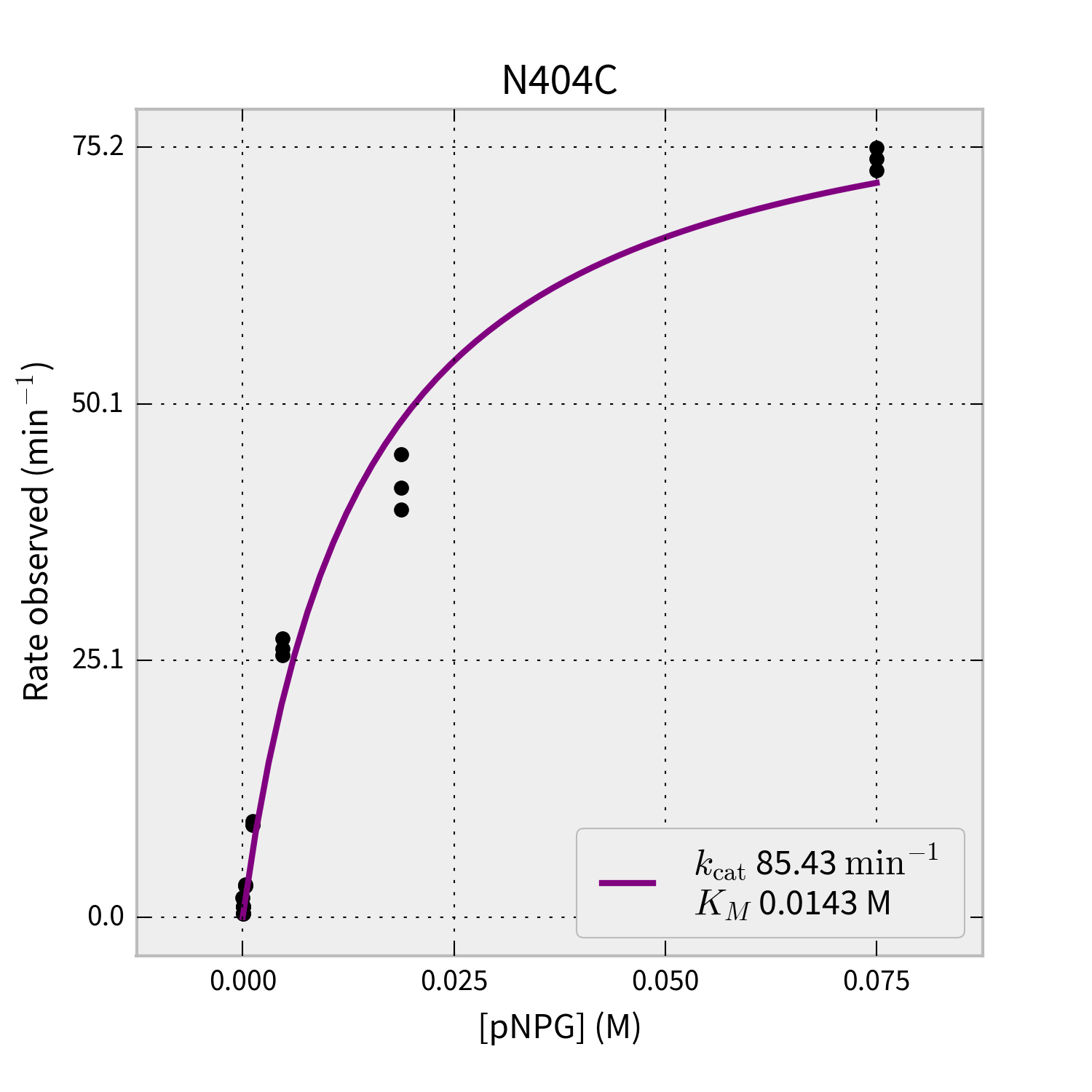

Supplement: S2 Figs — (ZIP) [file pone.0176255.s005.zip › S2 Figures/N404C.png]

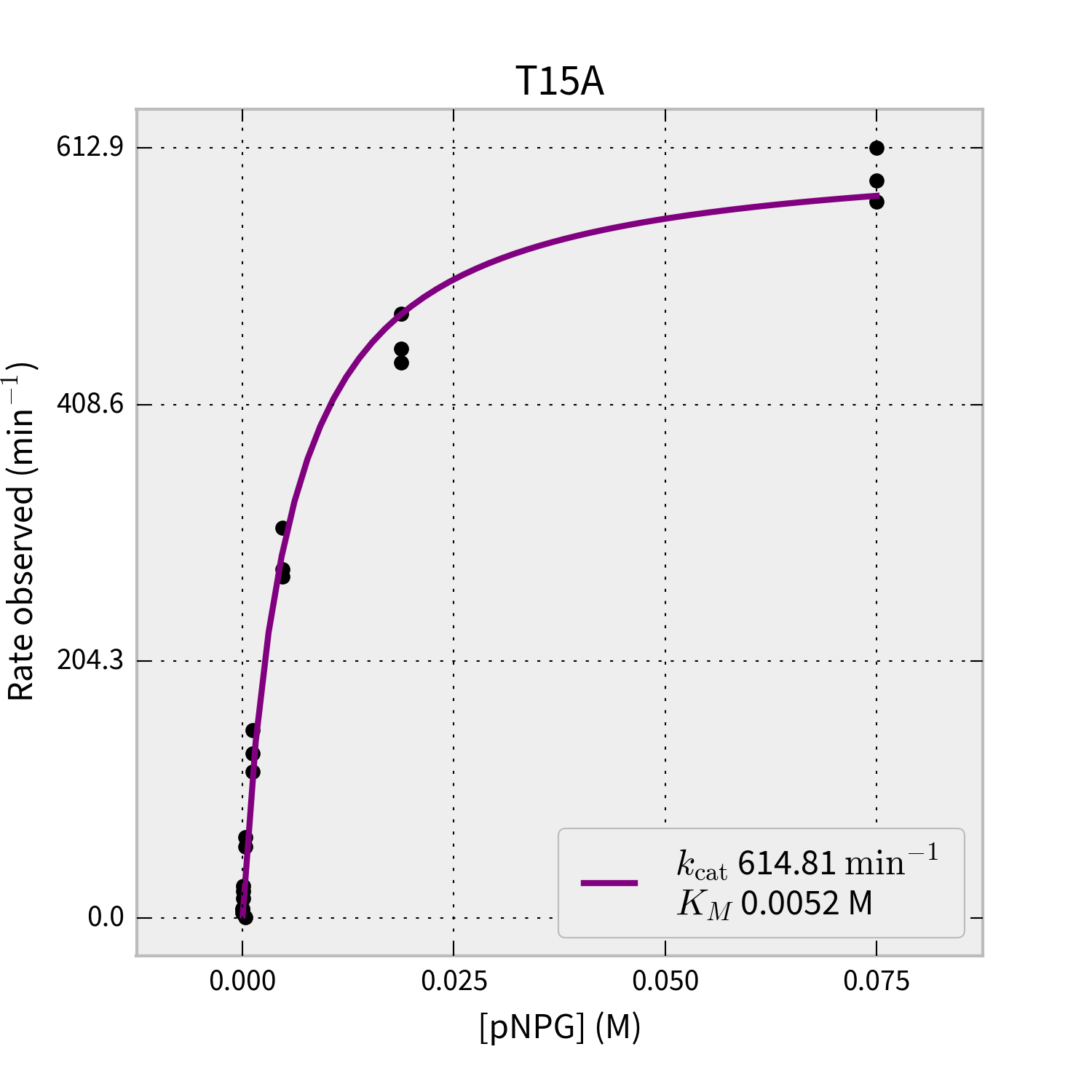

Supplement: S2 Figs — (ZIP) [file pone.0176255.s005.zip › S2 Figures/T15A.png]

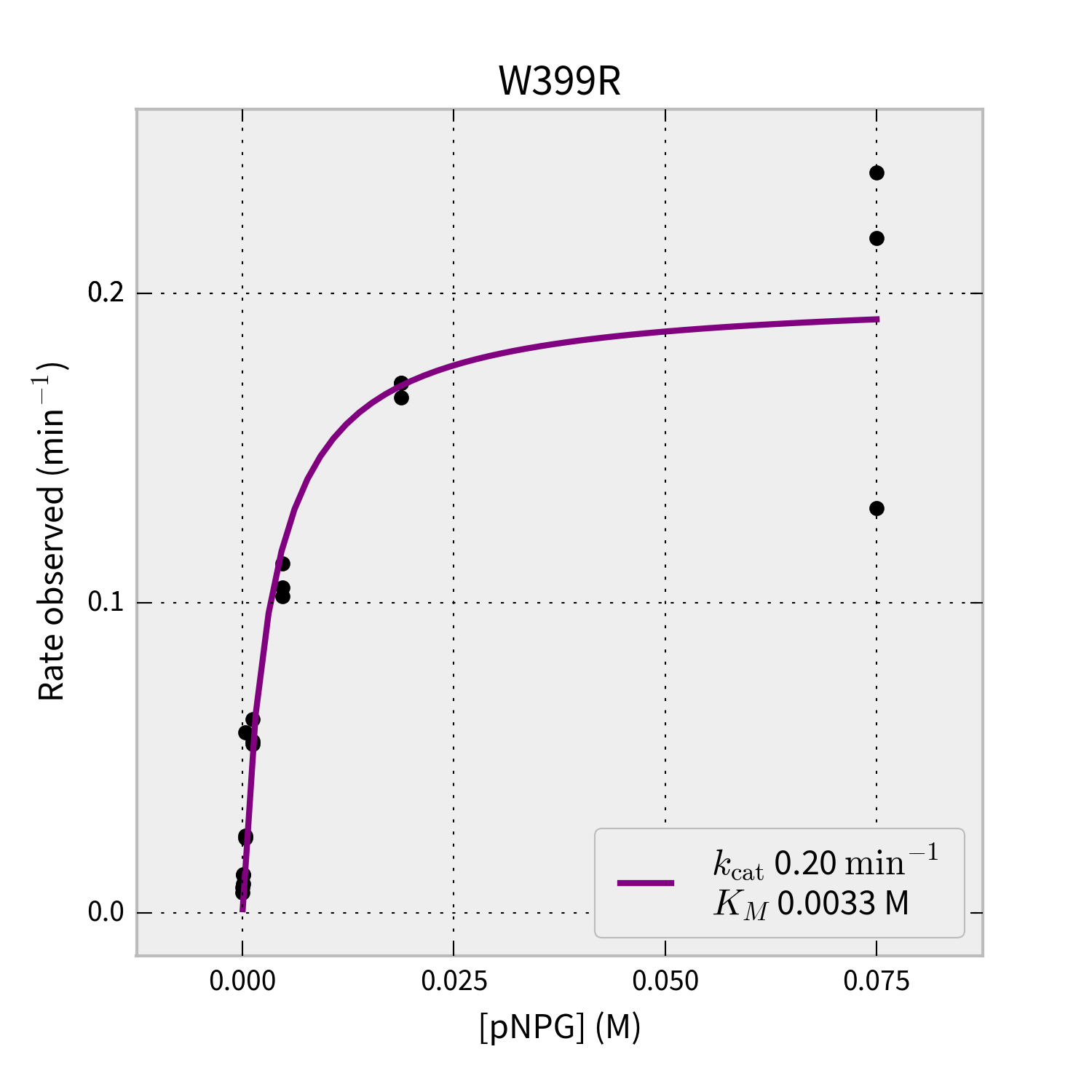

Supplement: S2 Figs — (ZIP) [file pone.0176255.s005.zip › S2 Figures/W399R.png]

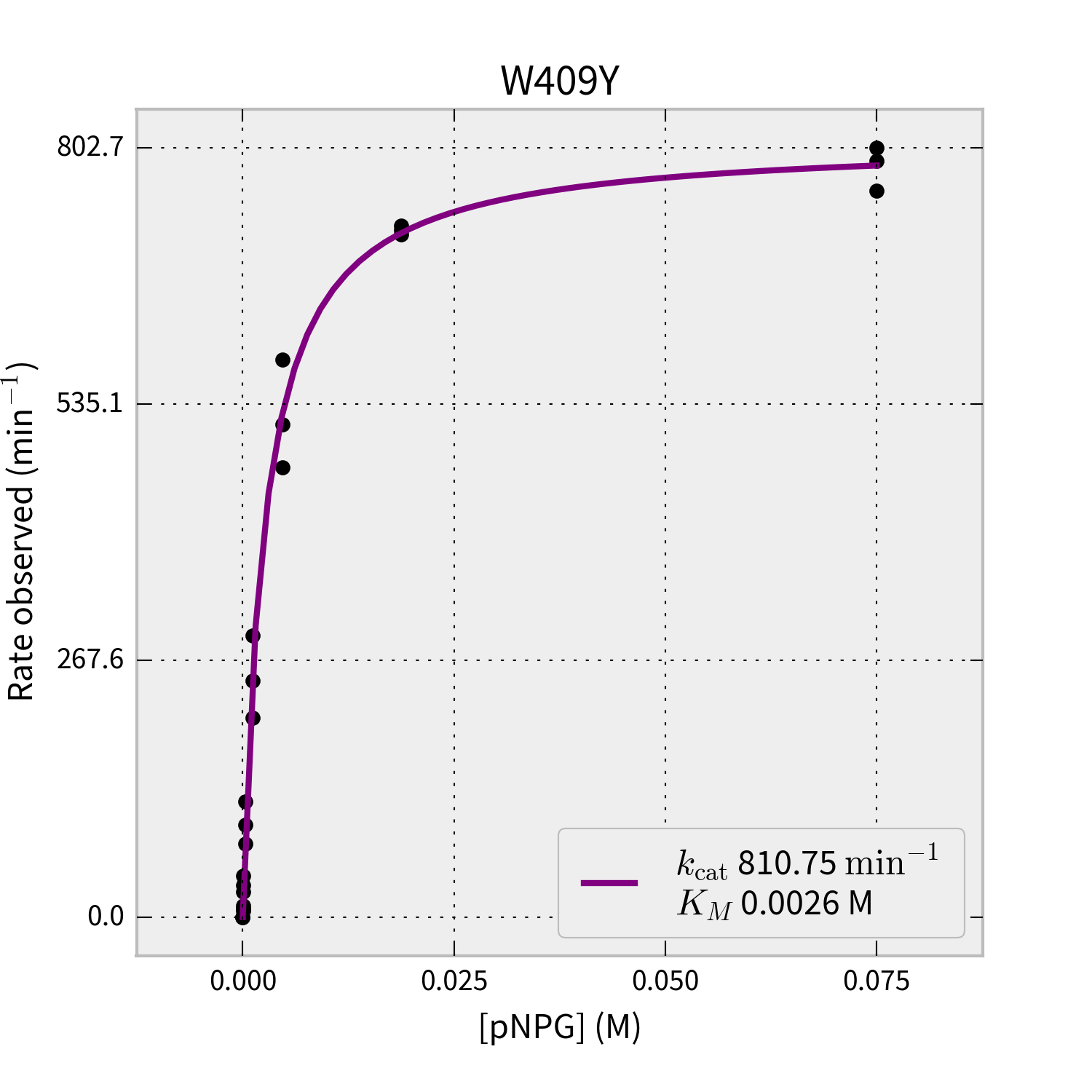

Supplement: S2 Figs — (ZIP) [file pone.0176255.s005.zip › S2 Figures/W409Y.png]

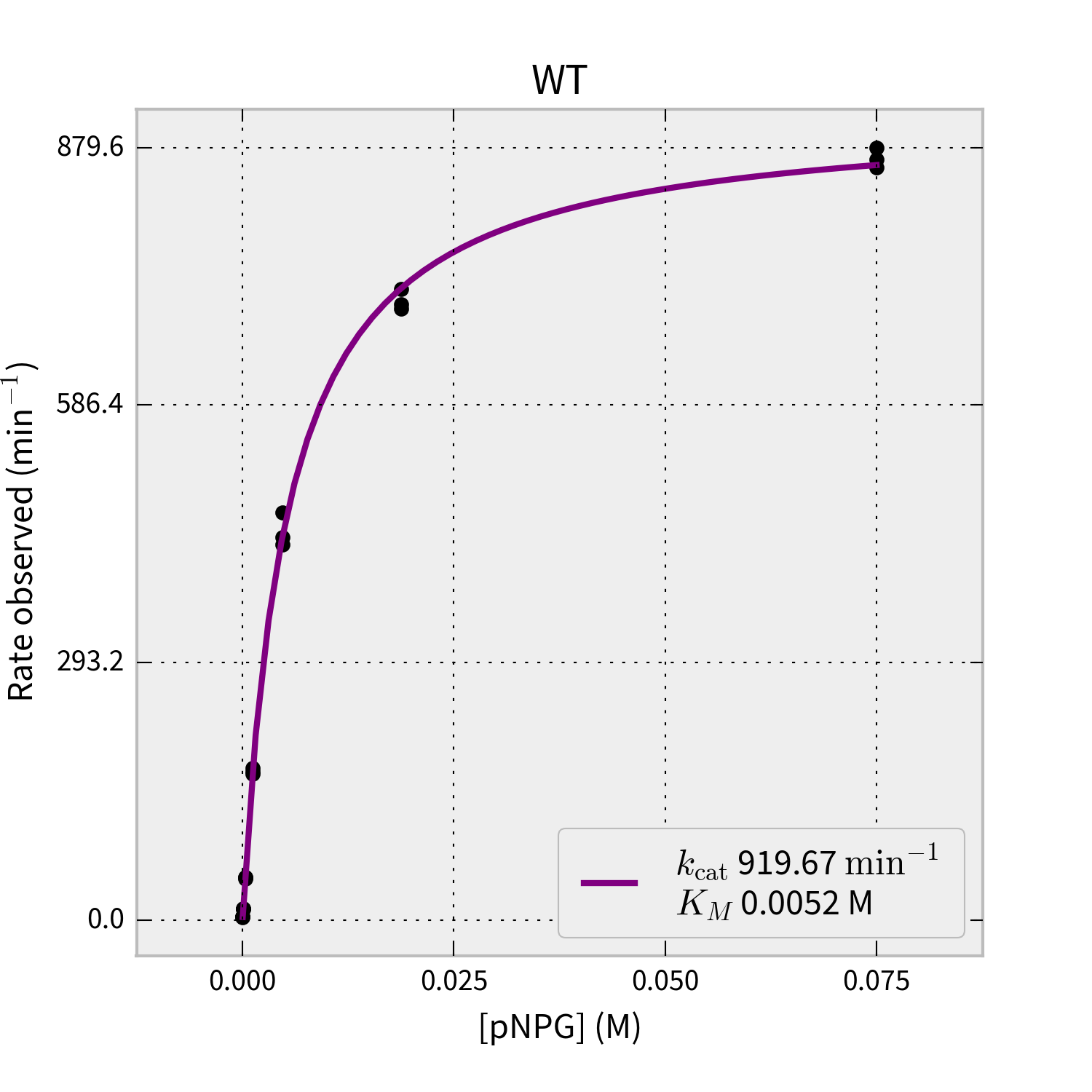

Supplement: S2 Figs — (ZIP) [file pone.0176255.s005.zip › S2 Figures/WT.png]

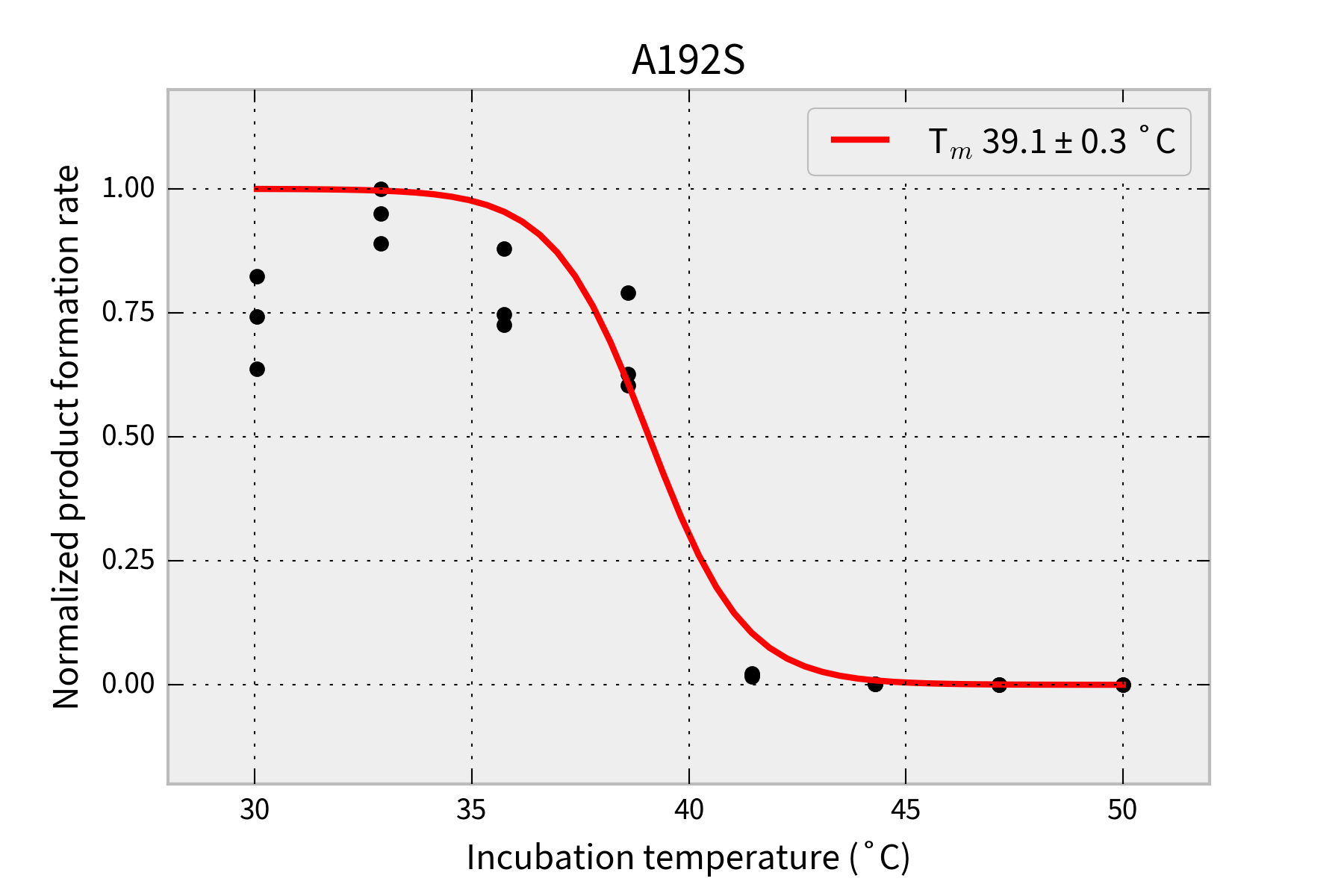

Supplement: S3 Figs — (ZIP) [file pone.0176255.s006.zip › S3 Figures/A192S.png]

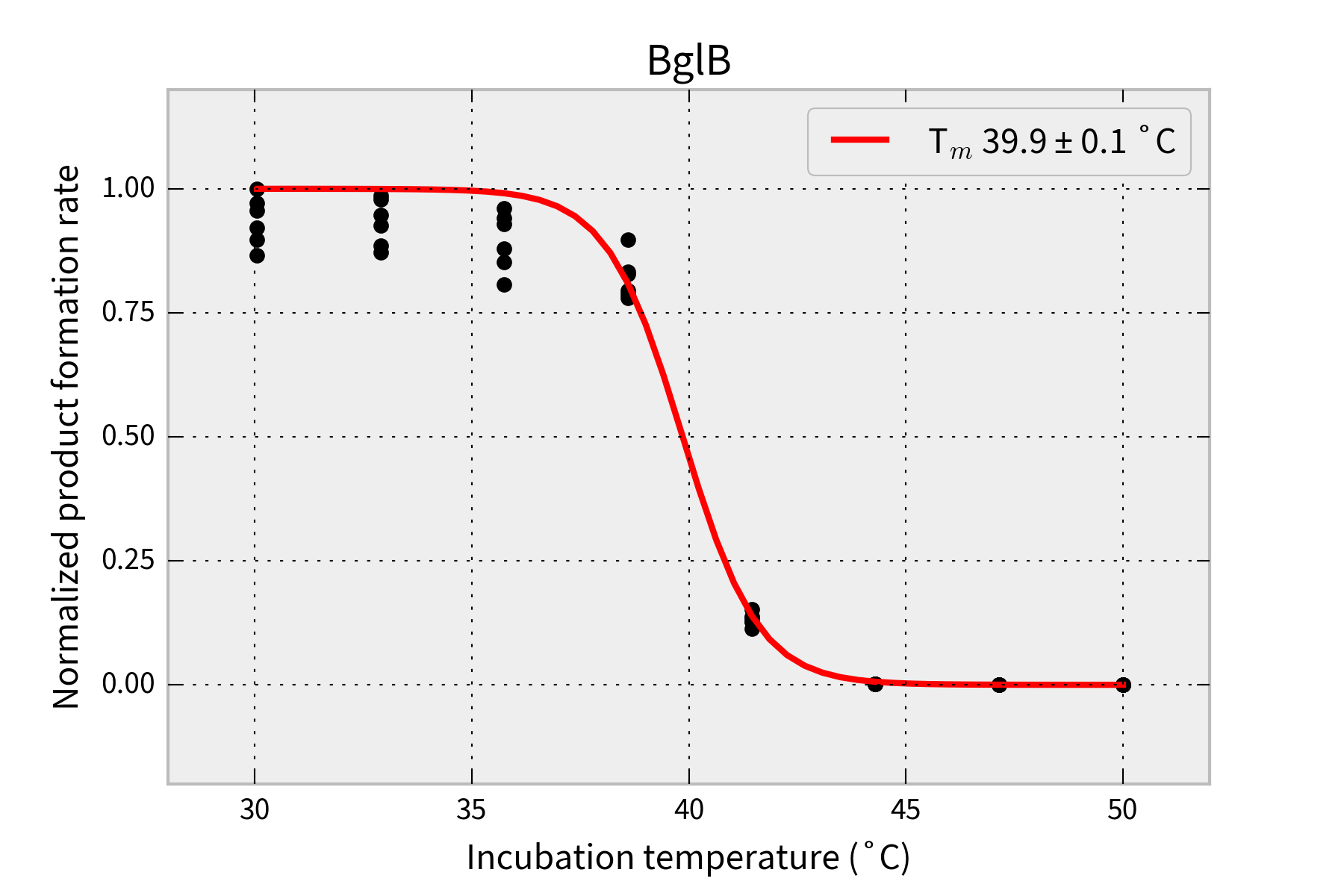

Supplement: S3 Figs — (ZIP) [file pone.0176255.s006.zip › S3 Figures/BglB.png]

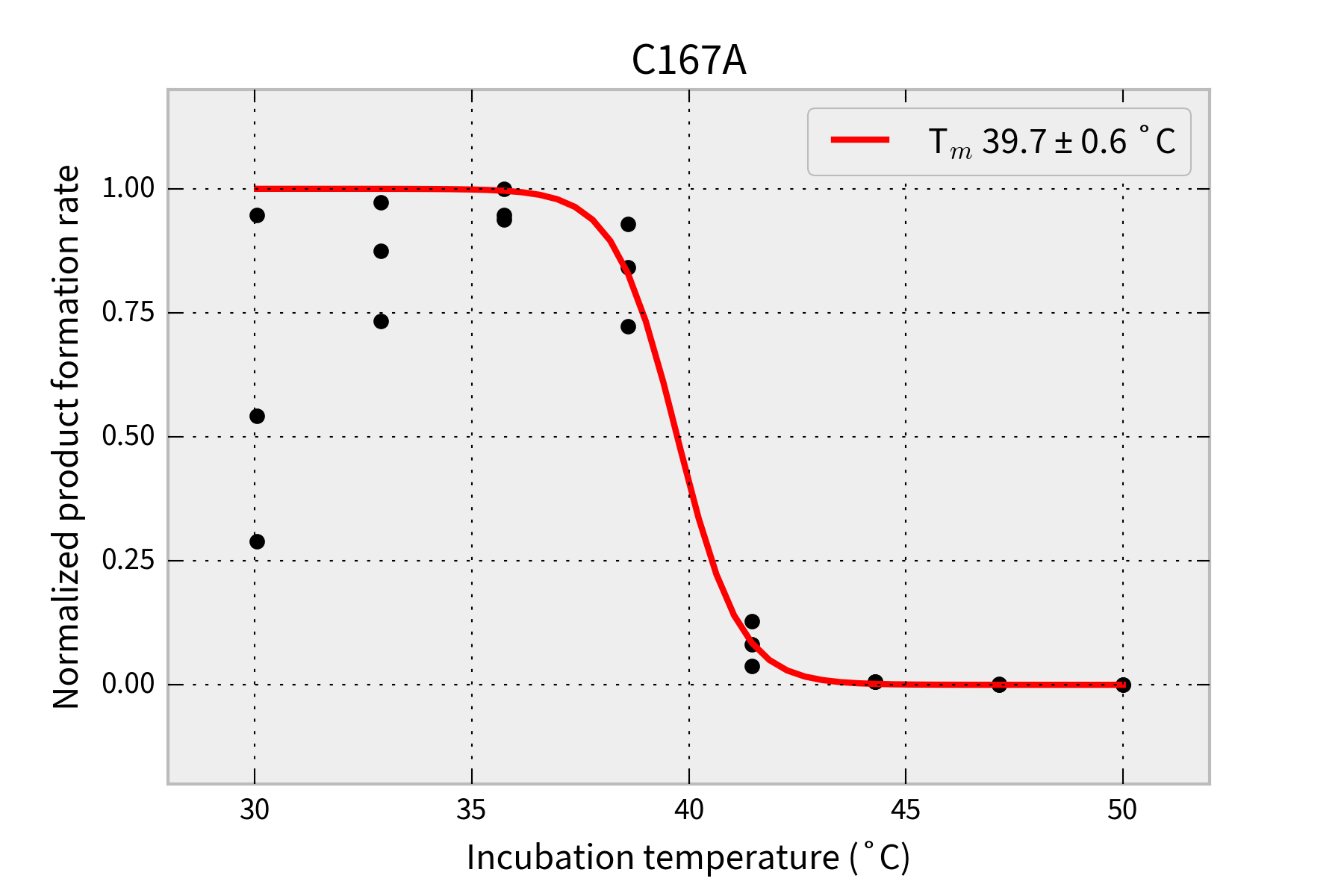

Supplement: S3 Figs — (ZIP) [file pone.0176255.s006.zip › S3 Figures/C167A.png]

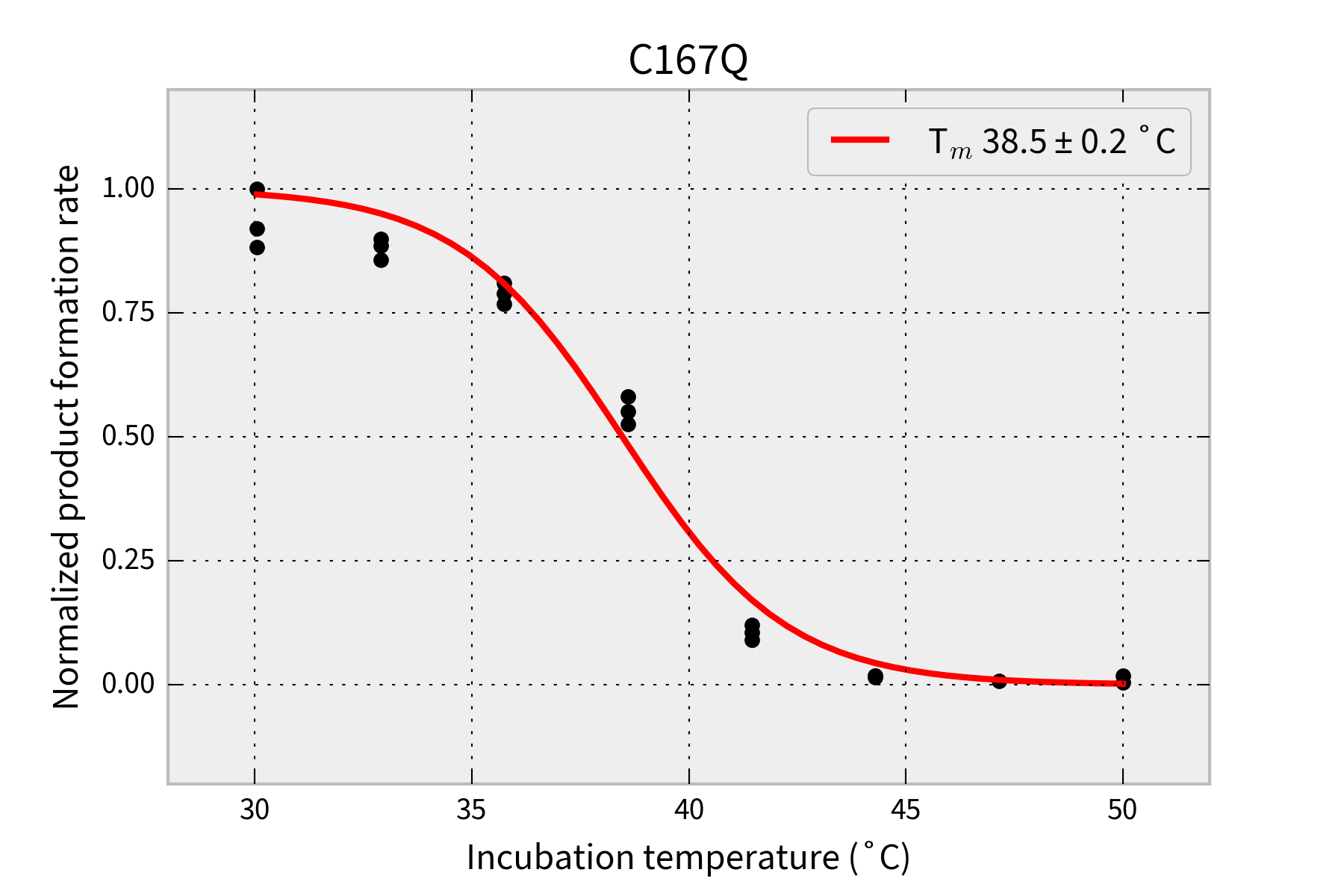

Supplement: S3 Figs — (ZIP) [file pone.0176255.s006.zip › S3 Figures/C167Q.png]

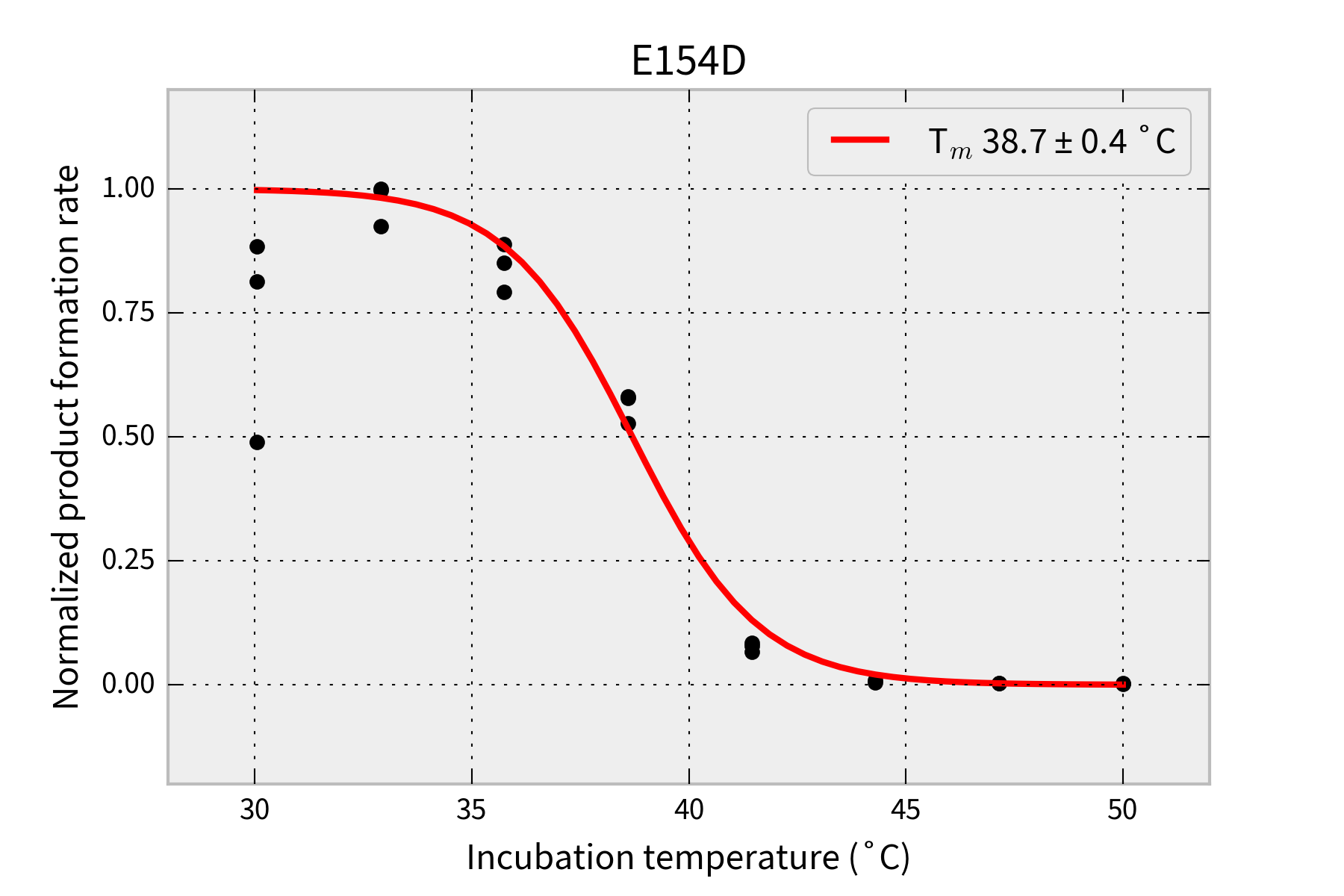

Supplement: S3 Figs — (ZIP) [file pone.0176255.s006.zip › S3 Figures/E154D.png]

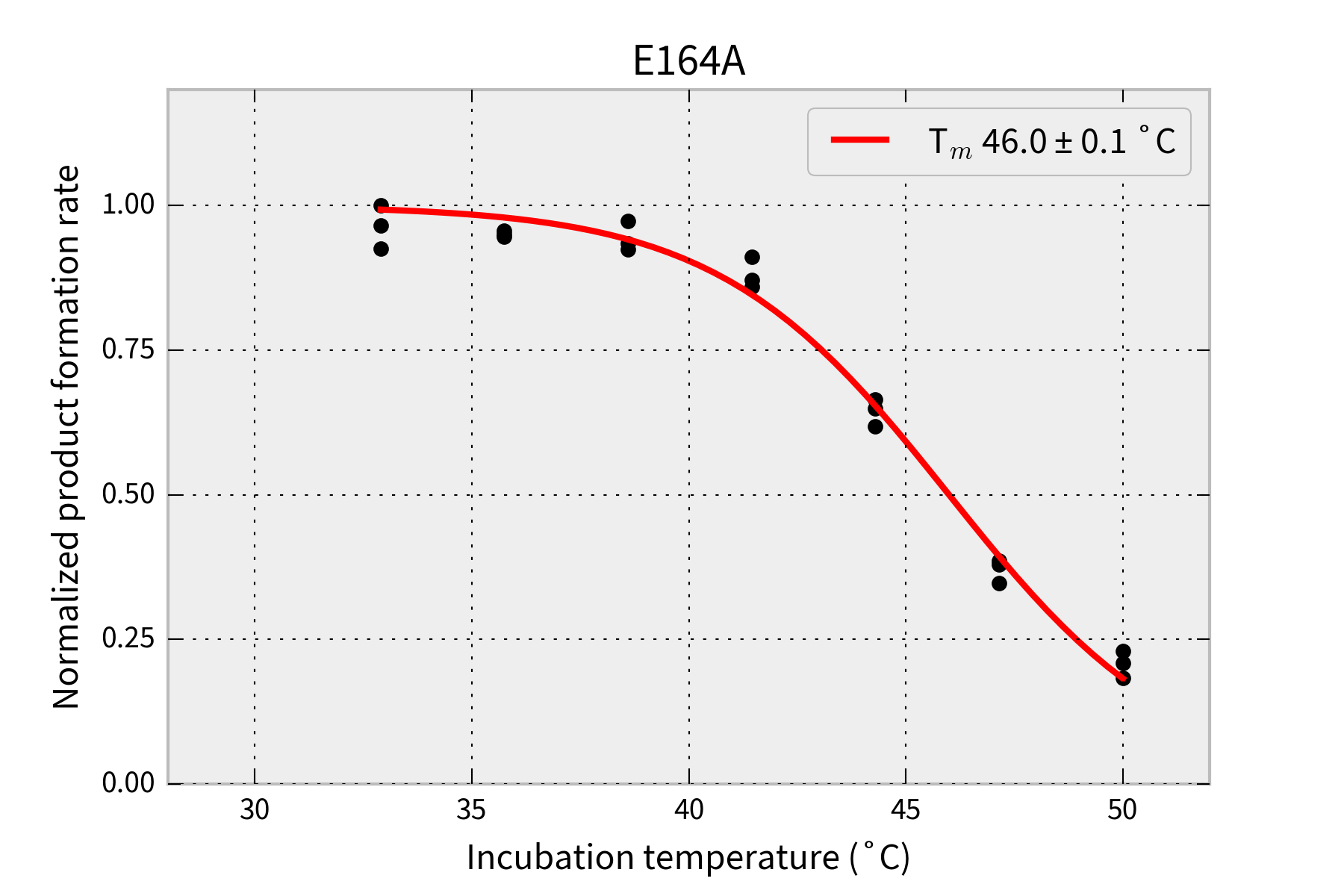

Supplement: S3 Figs — (ZIP) [file pone.0176255.s006.zip › S3 Figures/E164A.png]

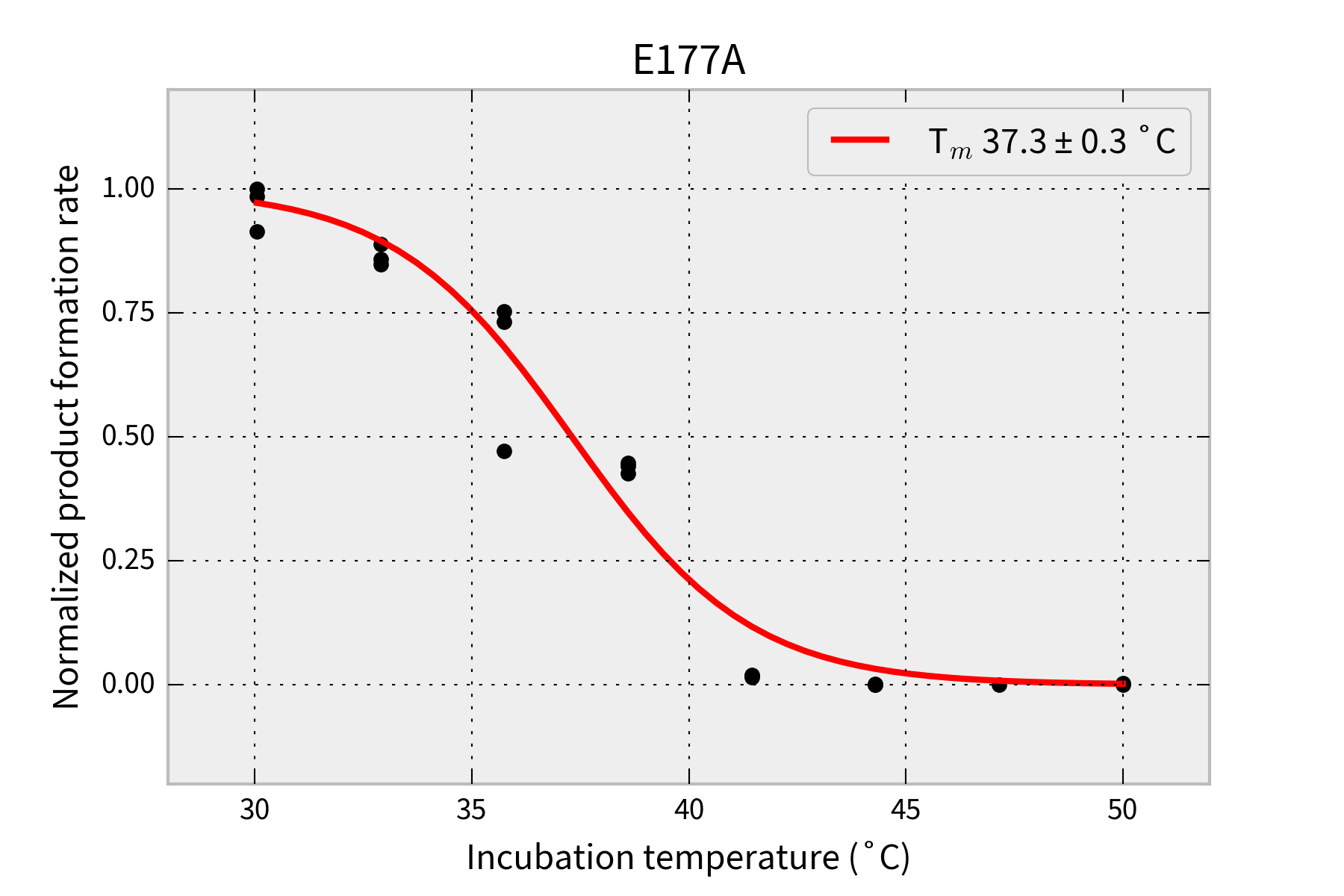

Supplement: S3 Figs — (ZIP) [file pone.0176255.s006.zip › S3 Figures/E177A.png]

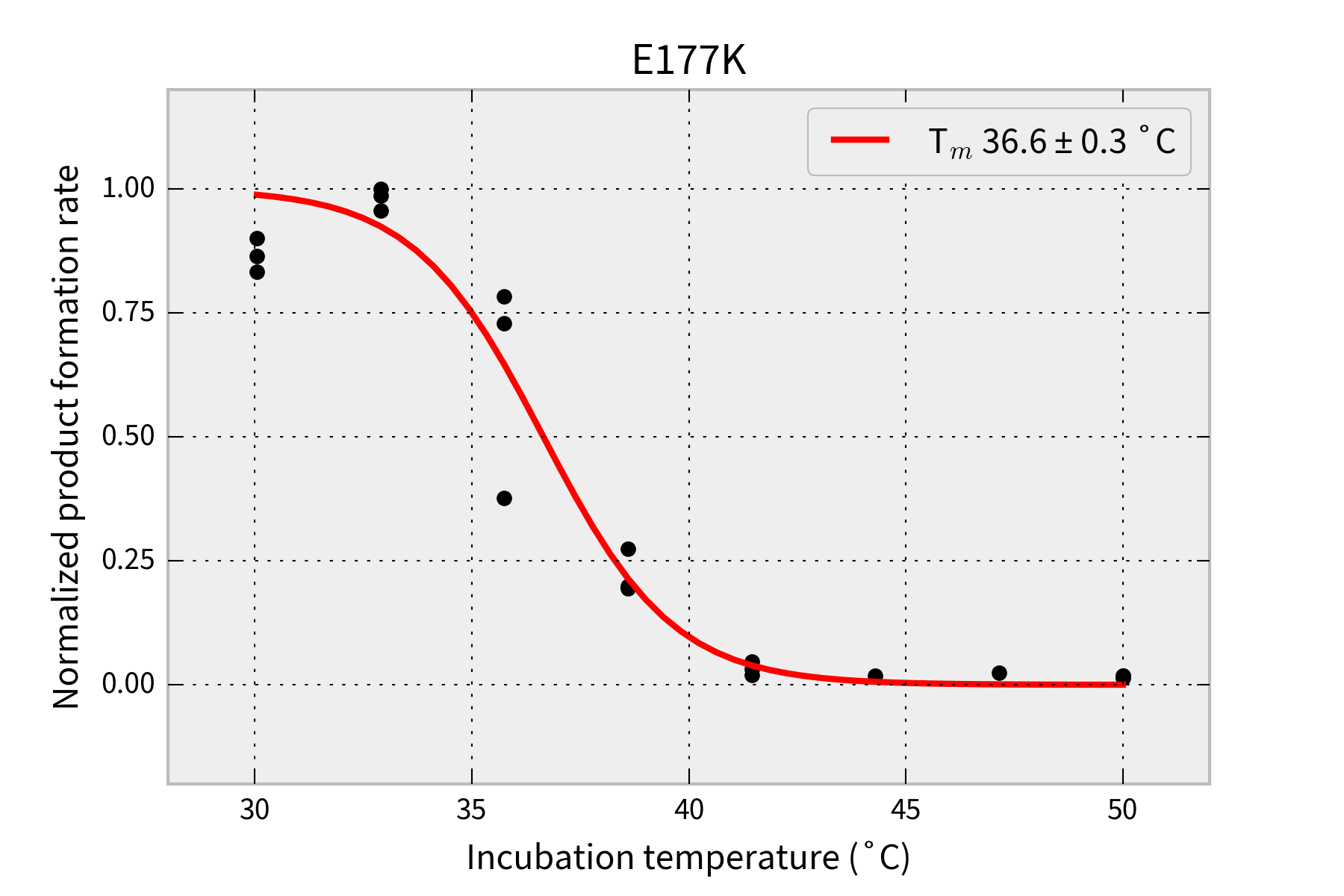

Supplement: S3 Figs — (ZIP) [file pone.0176255.s006.zip › S3 Figures/E177K.png]

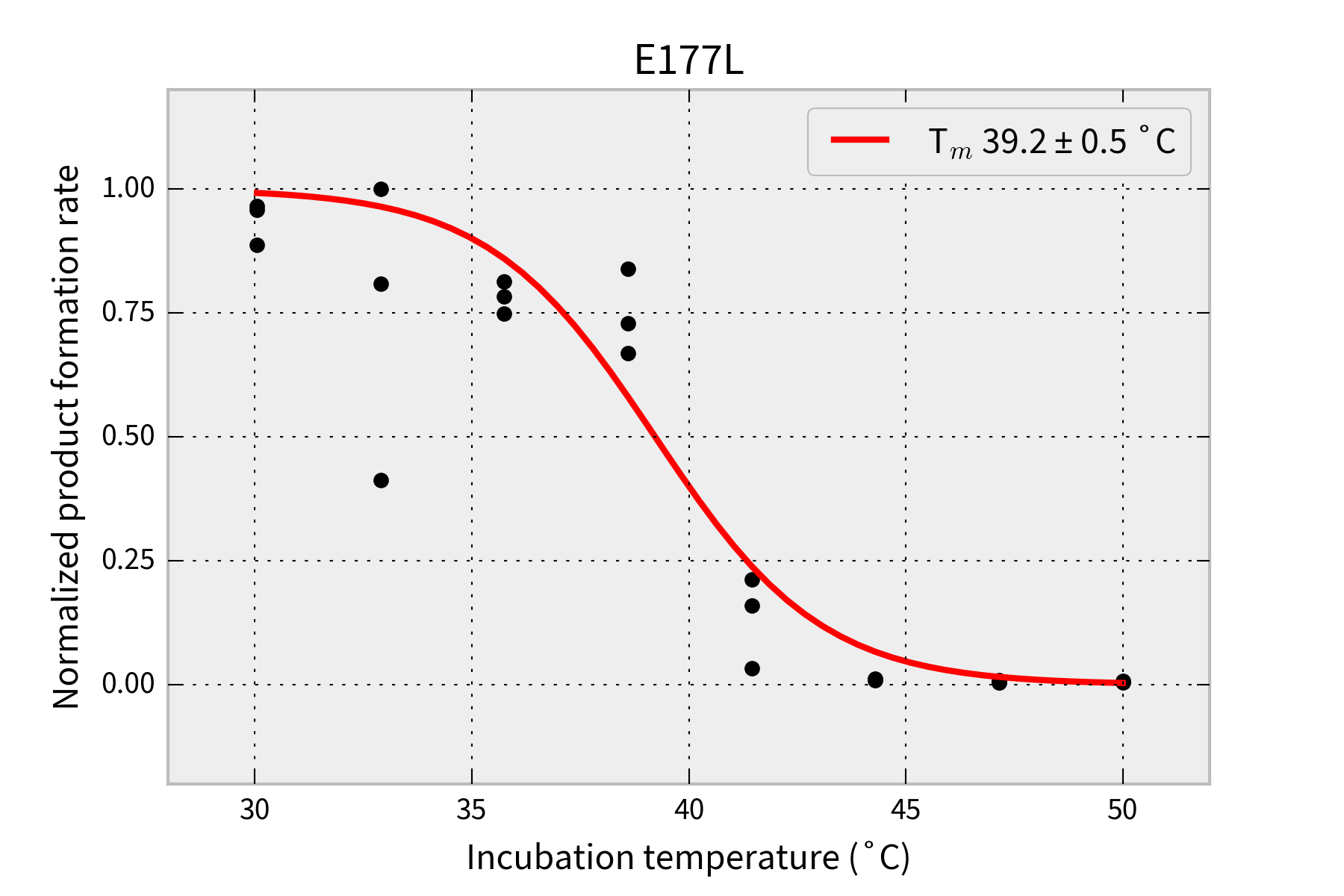

Supplement: S3 Figs — (ZIP) [file pone.0176255.s006.zip › S3 Figures/E177L.png]

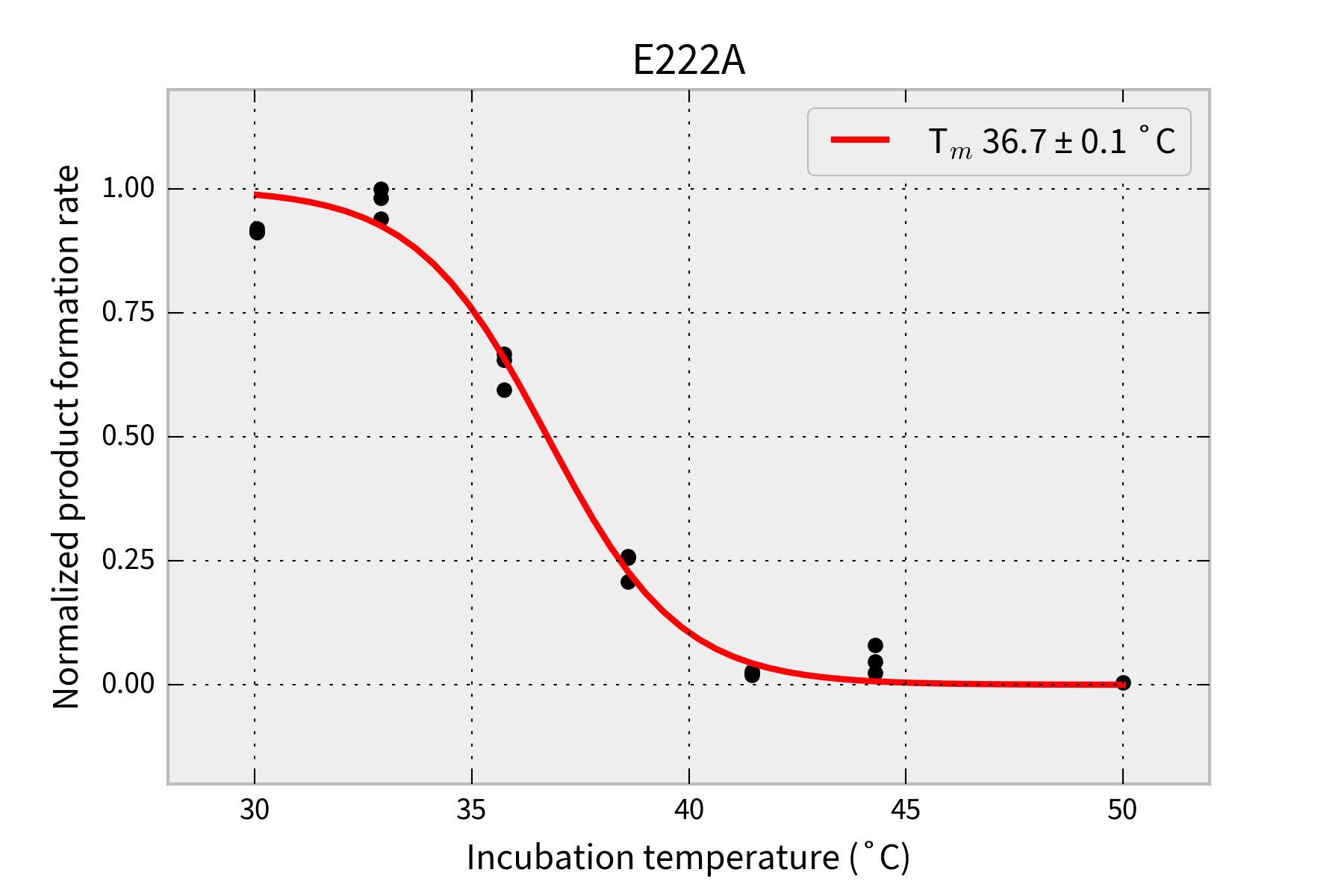

Supplement: S3 Figs — (ZIP) [file pone.0176255.s006.zip › S3 Figures/E222A.png]

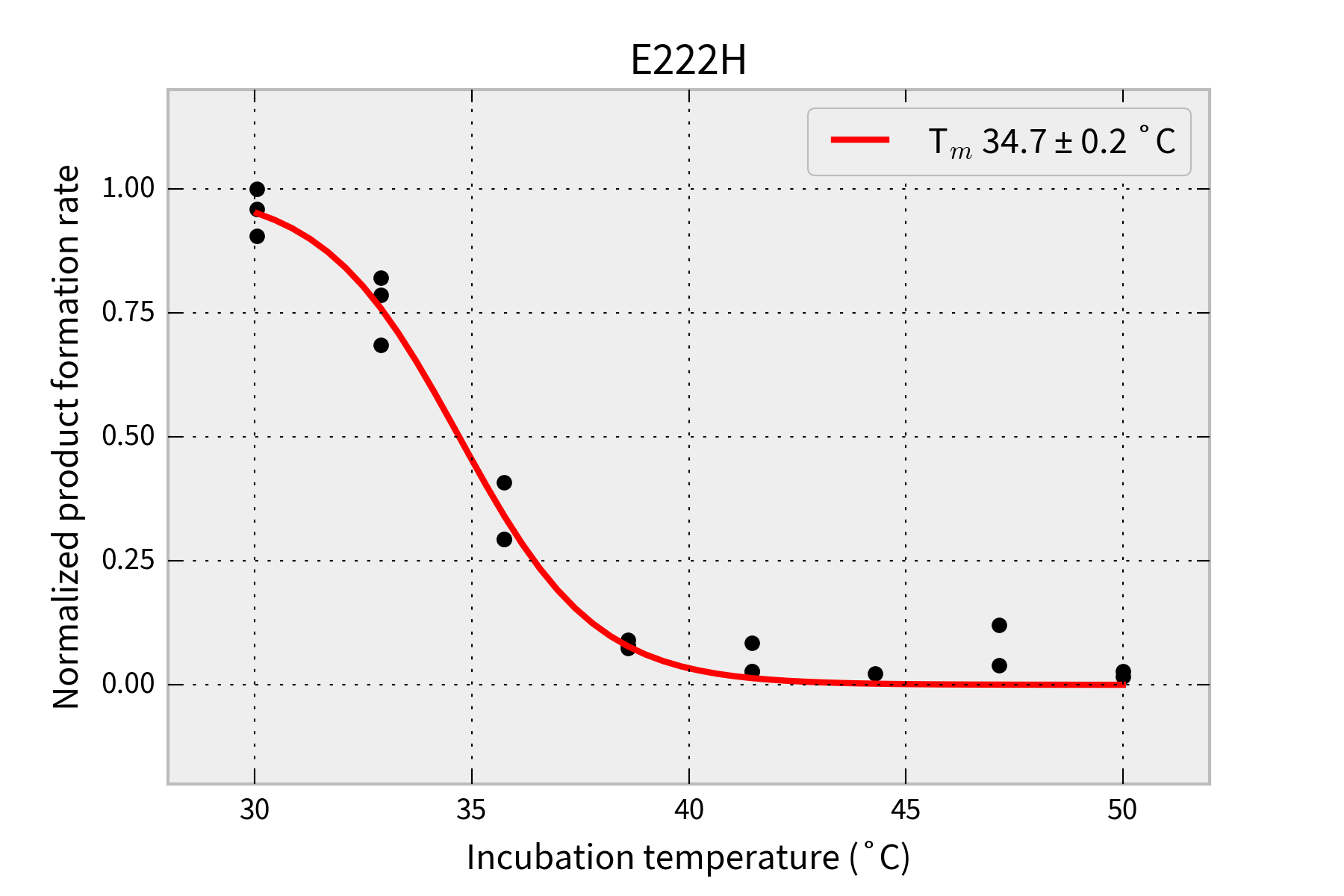

Supplement: S3 Figs — (ZIP) [file pone.0176255.s006.zip › S3 Figures/E222H.png]

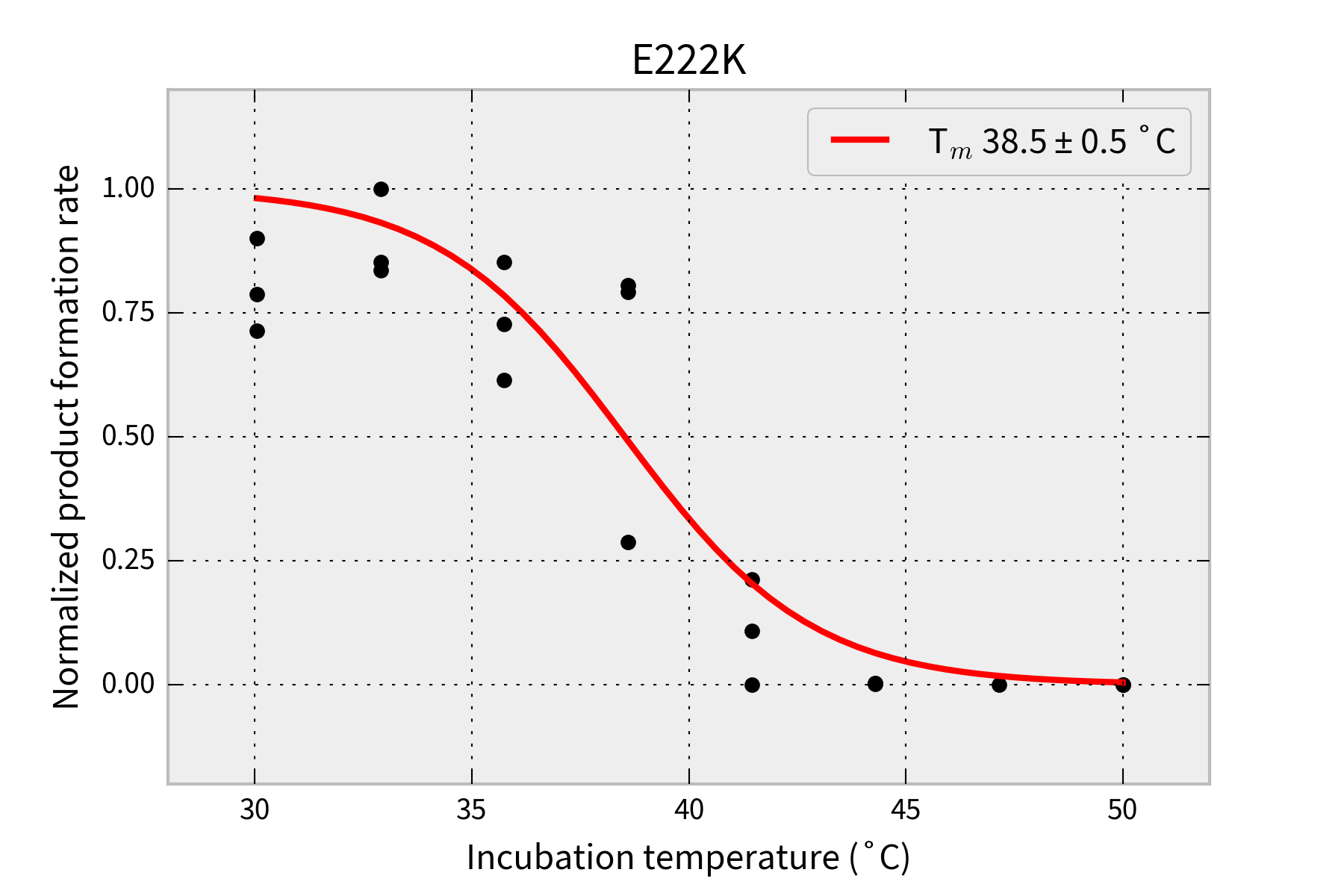

Supplement: S3 Figs — (ZIP) [file pone.0176255.s006.zip › S3 Figures/E222K.png]

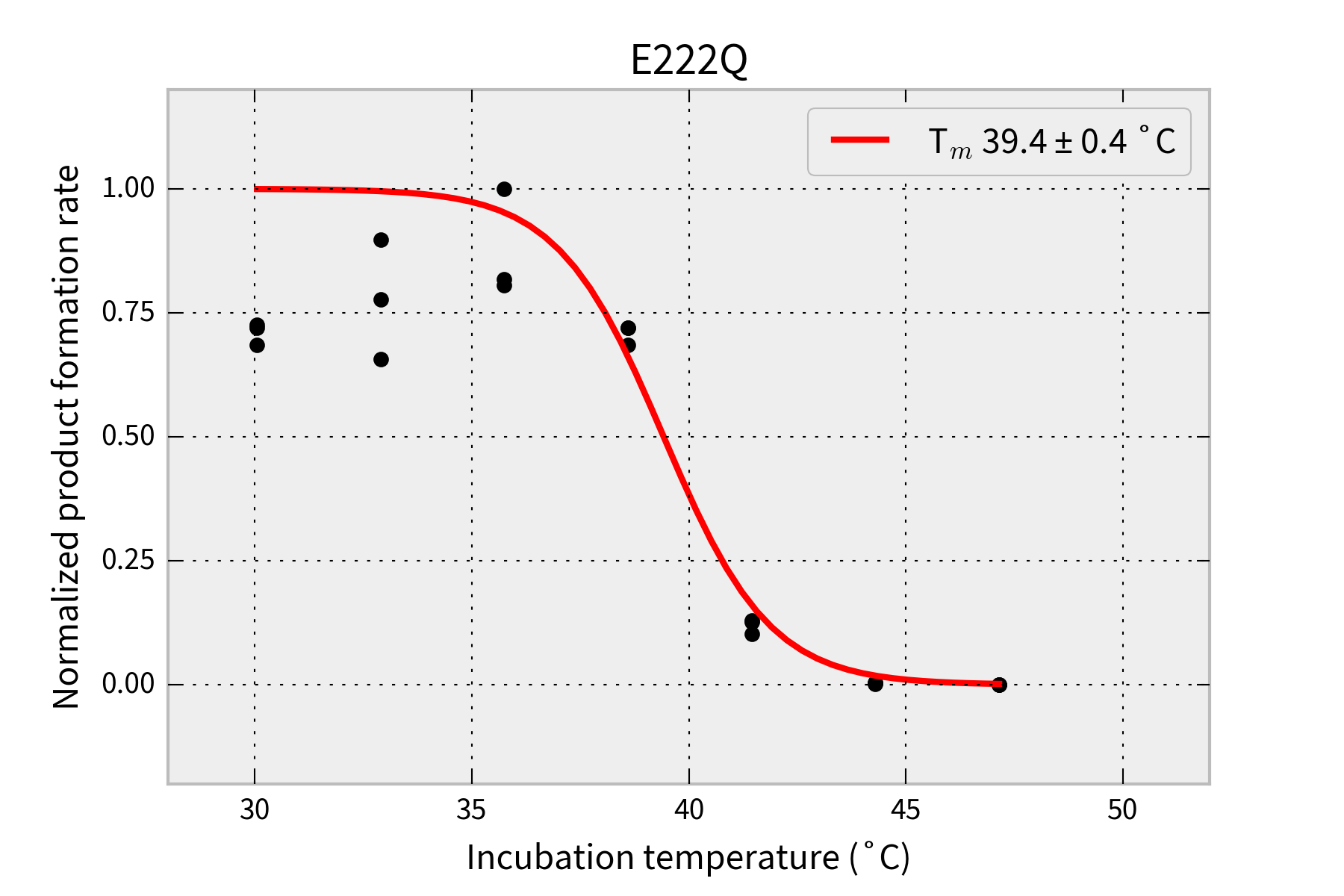

Supplement: S3 Figs — (ZIP) [file pone.0176255.s006.zip › S3 Figures/E222Q.png]

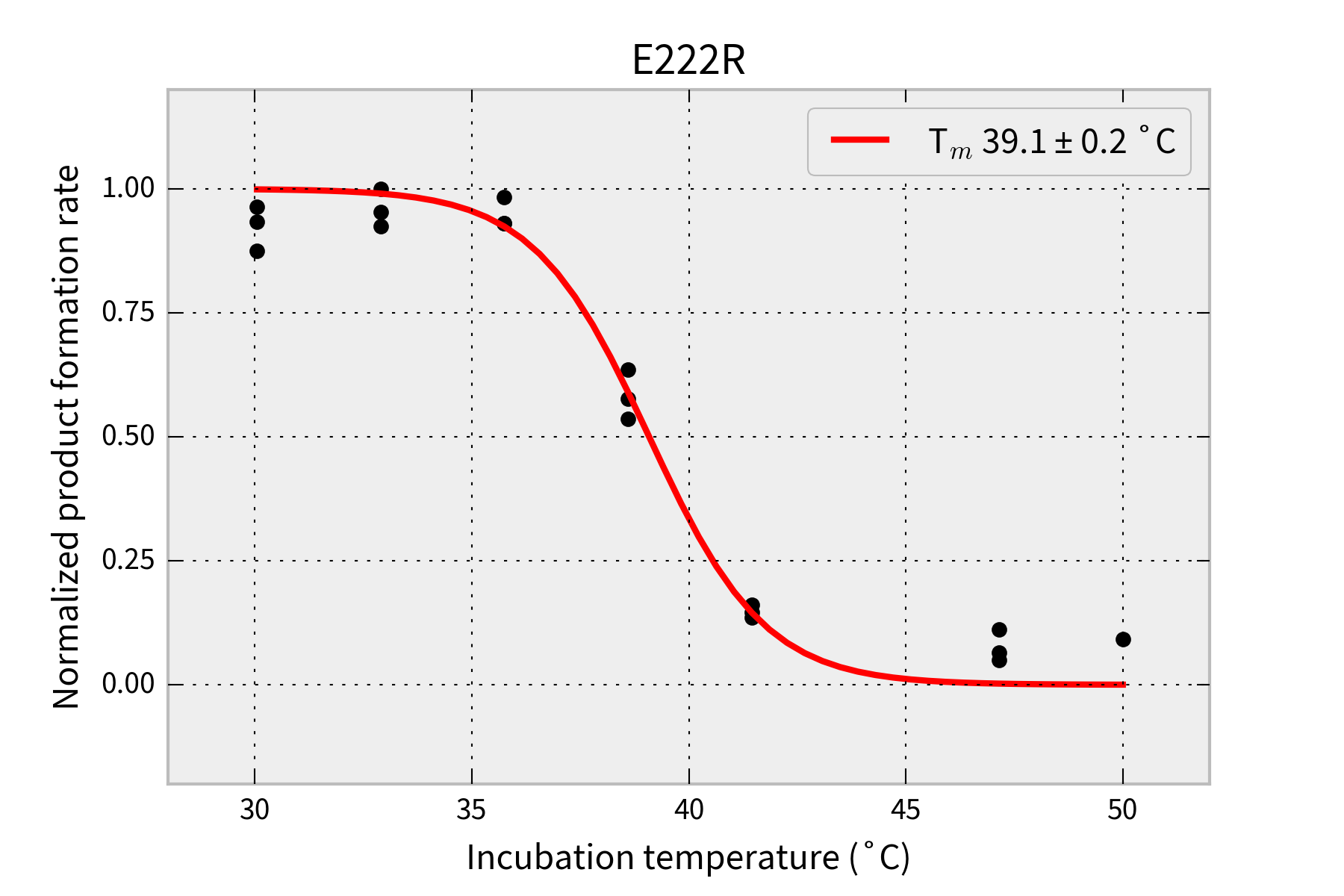

Supplement: S3 Figs — (ZIP) [file pone.0176255.s006.zip › S3 Figures/E222R.png]

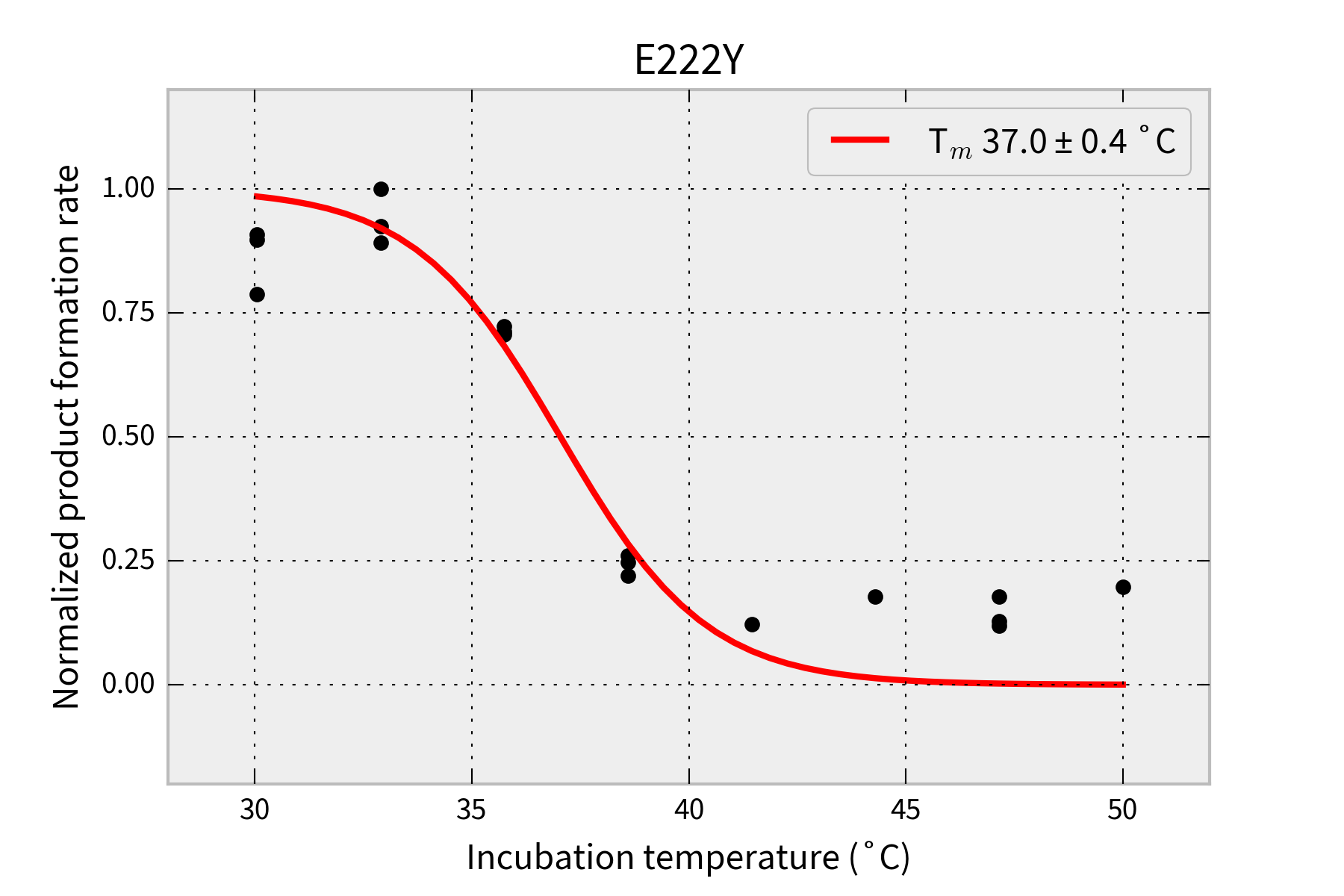

Supplement: S3 Figs — (ZIP) [file pone.0176255.s006.zip › S3 Figures/E222Y.png]

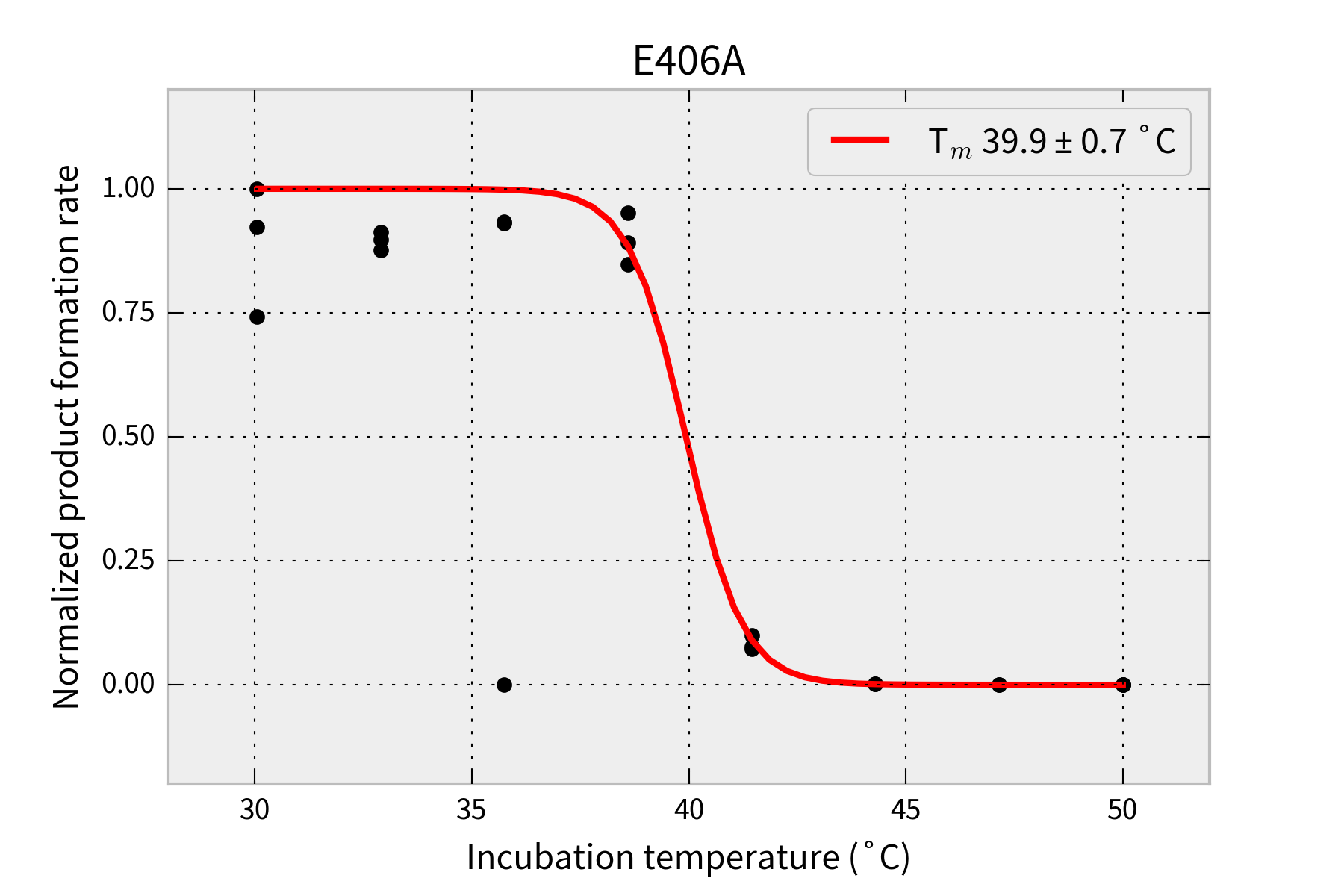

Supplement: S3 Figs — (ZIP) [file pone.0176255.s006.zip › S3 Figures/E406A.png]

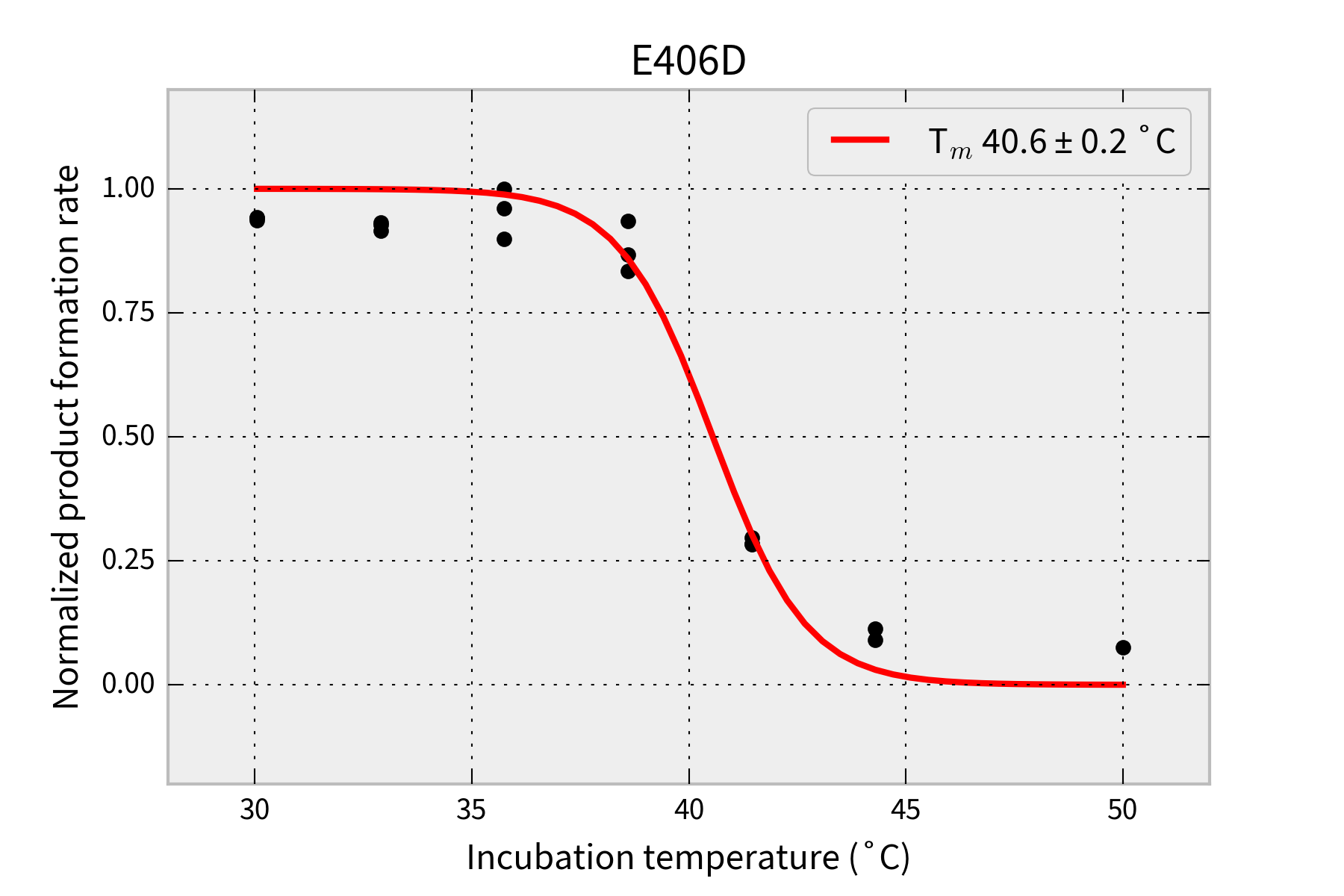

Supplement: S3 Figs — (ZIP) [file pone.0176255.s006.zip › S3 Figures/E406D.png]

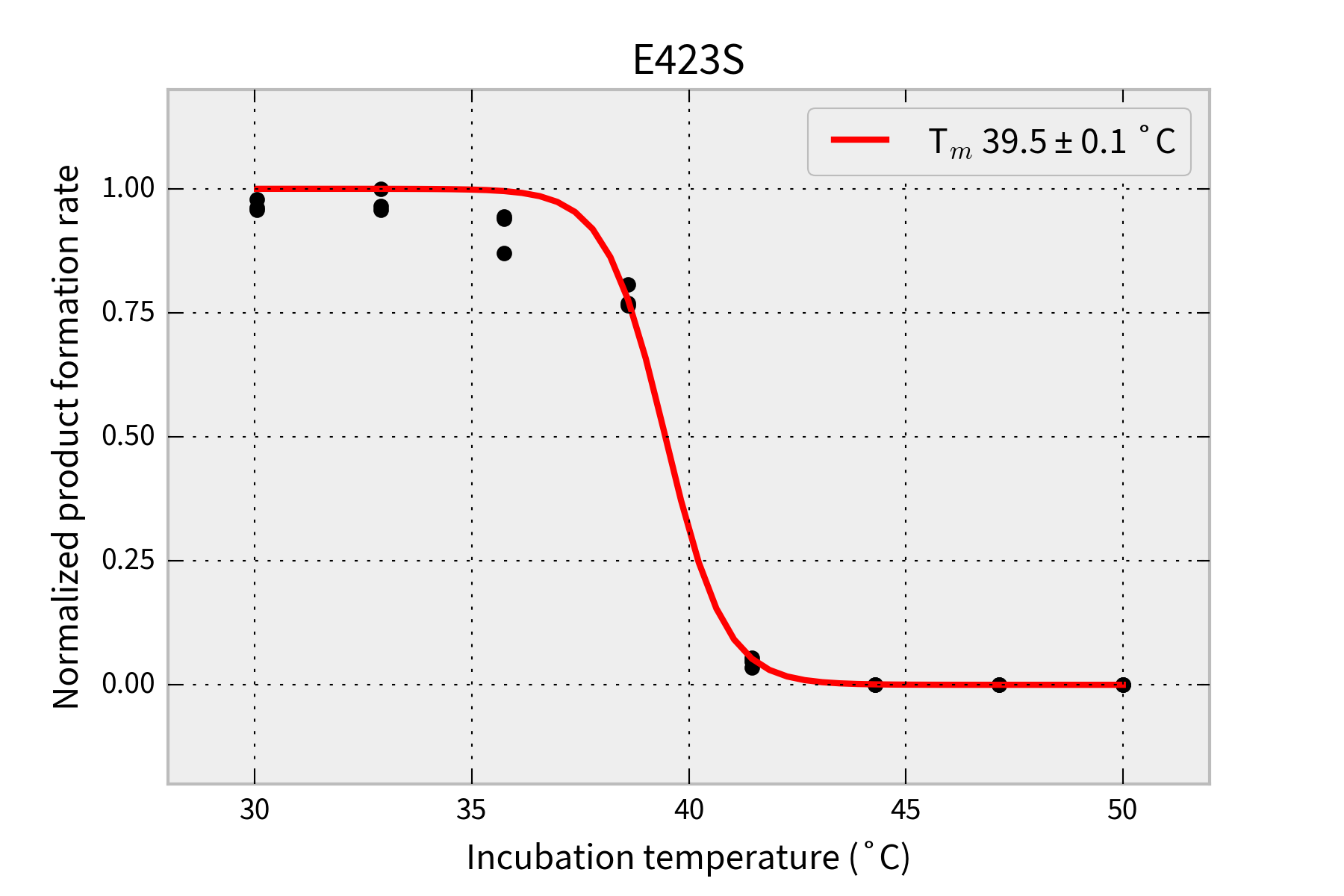

Supplement: S3 Figs — (ZIP) [file pone.0176255.s006.zip › S3 Figures/E423S.png]

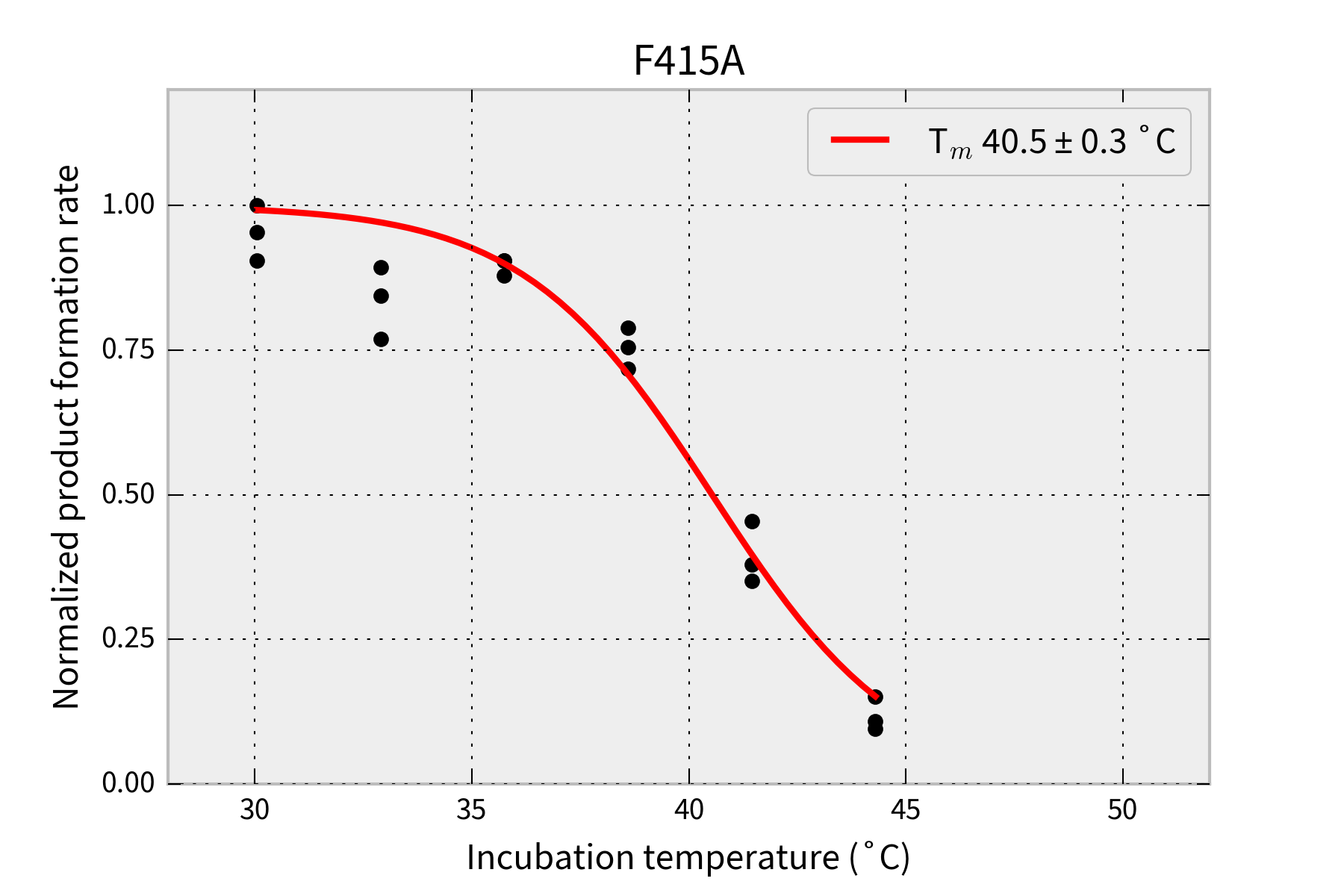

Supplement: S3 Figs — (ZIP) [file pone.0176255.s006.zip › S3 Figures/F415A.png]

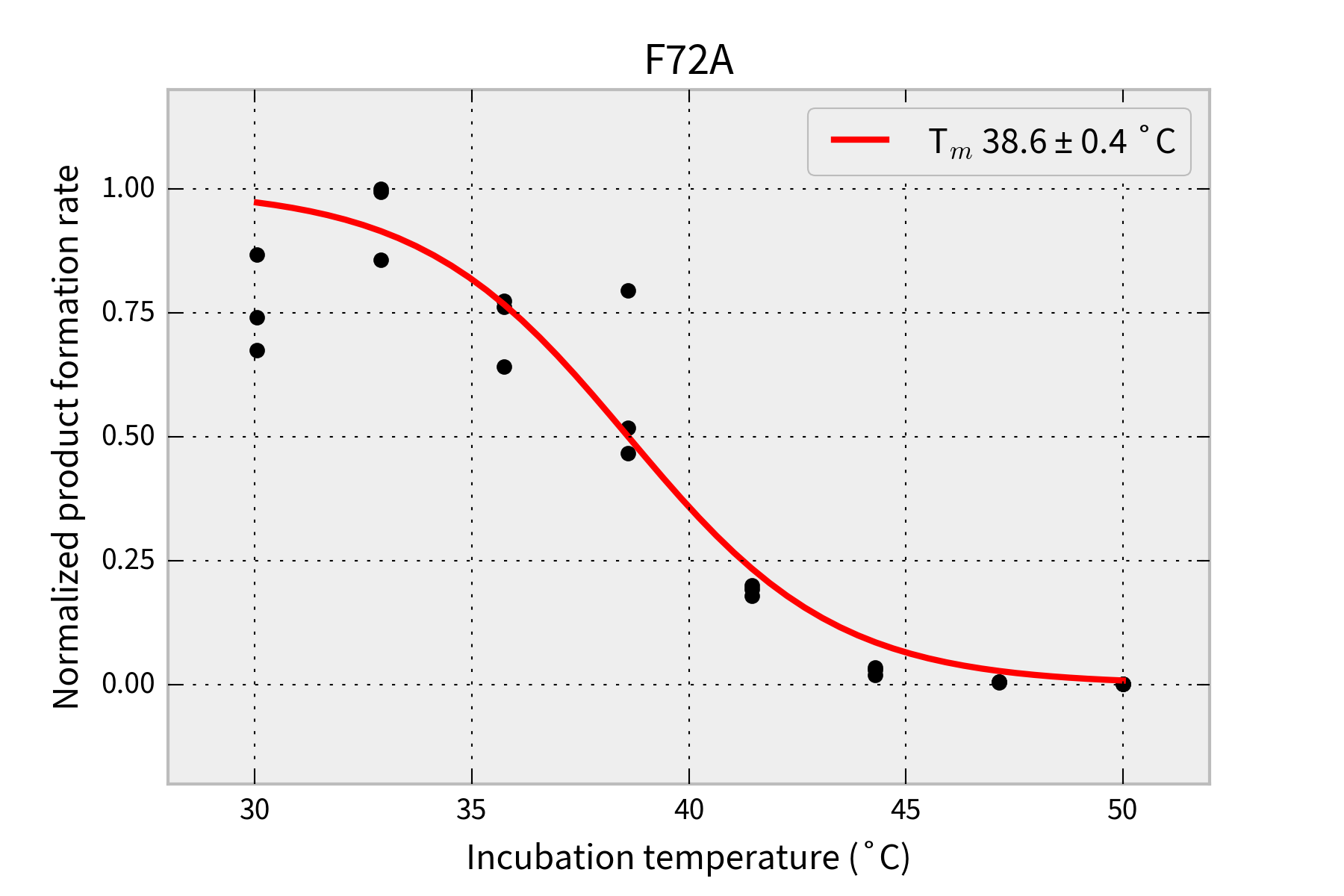

Supplement: S3 Figs — (ZIP) [file pone.0176255.s006.zip › S3 Figures/F72A.png]

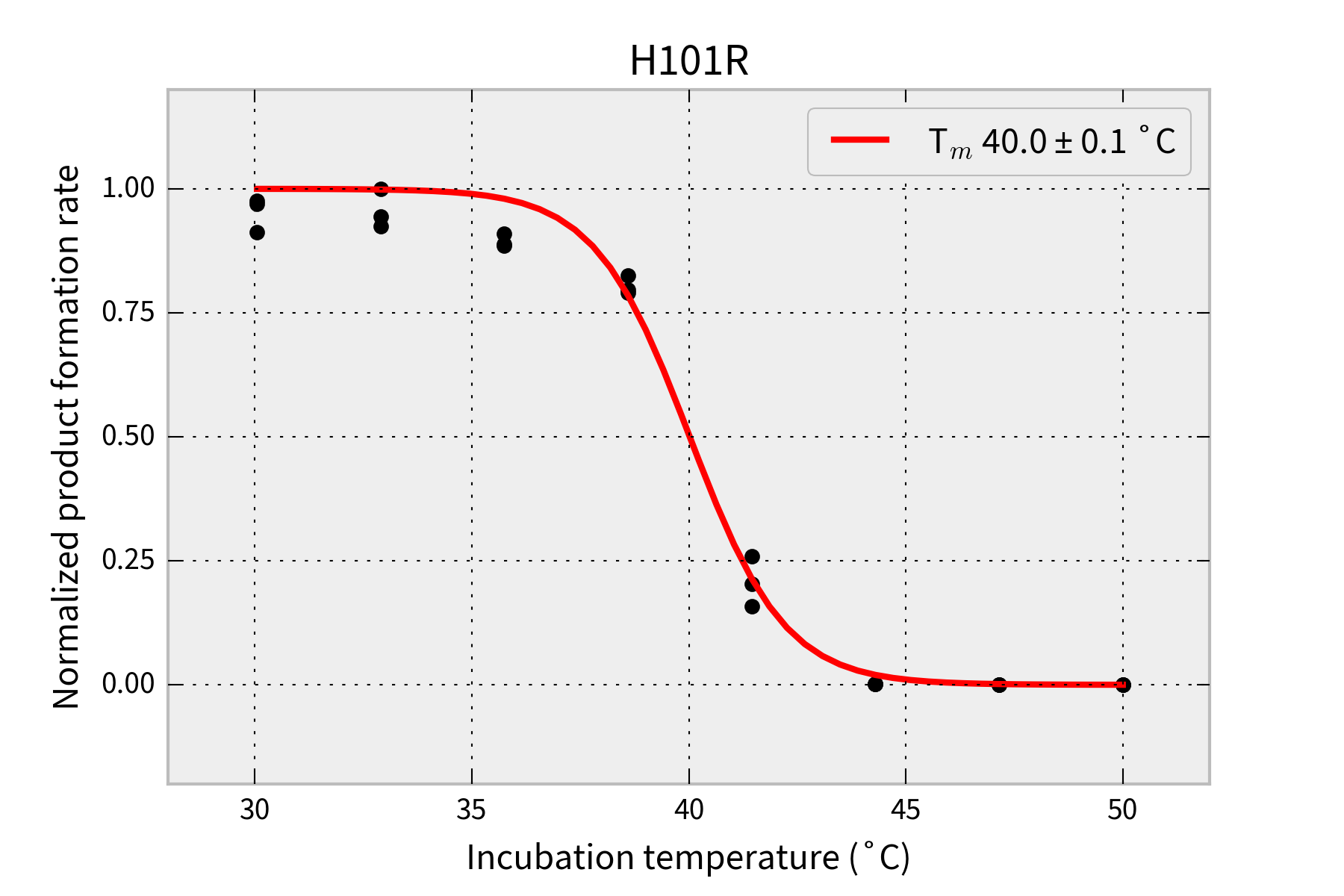

Supplement: S3 Figs — (ZIP) [file pone.0176255.s006.zip › S3 Figures/H101R.png]

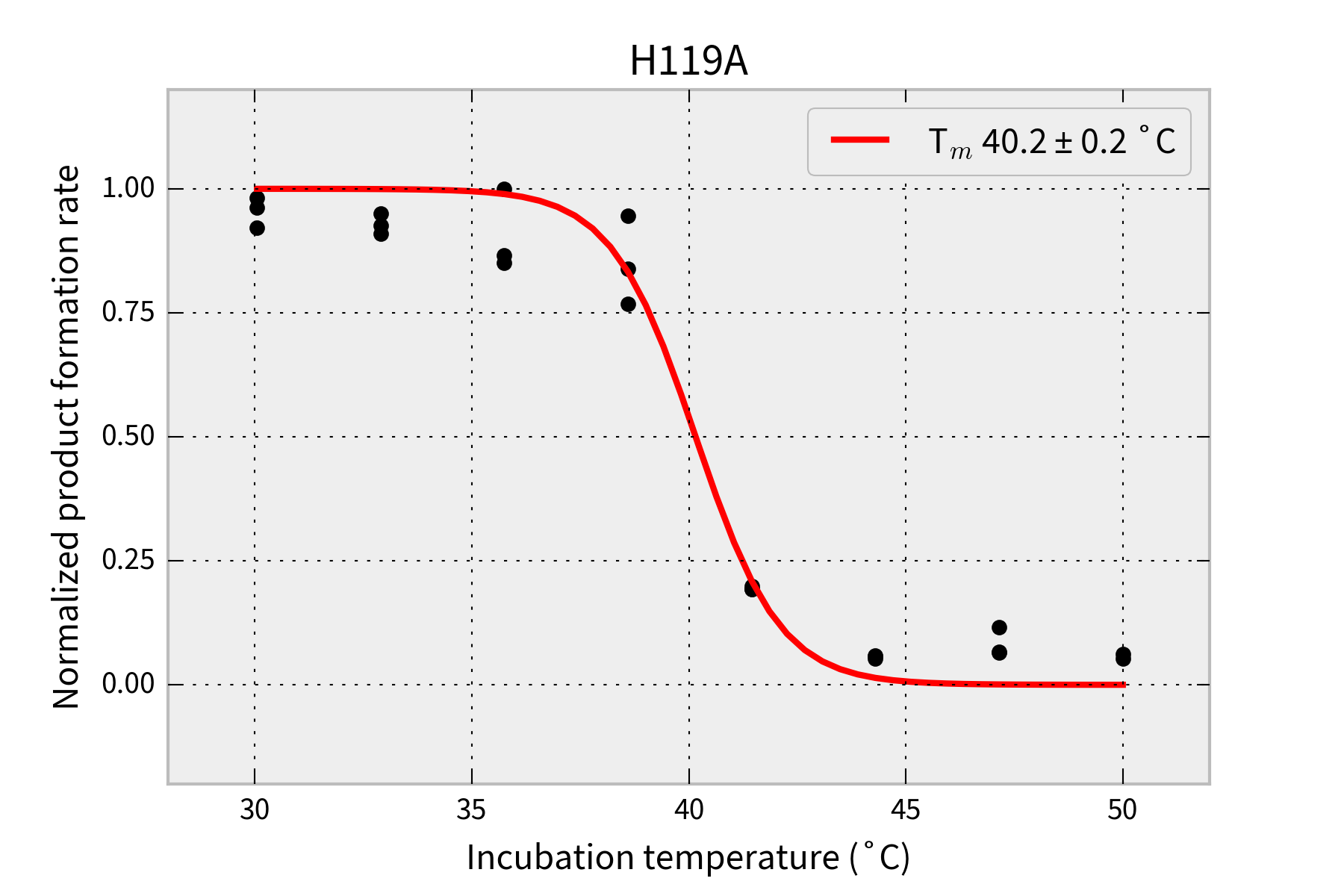

Supplement: S3 Figs — (ZIP) [file pone.0176255.s006.zip › S3 Figures/H119A.png]

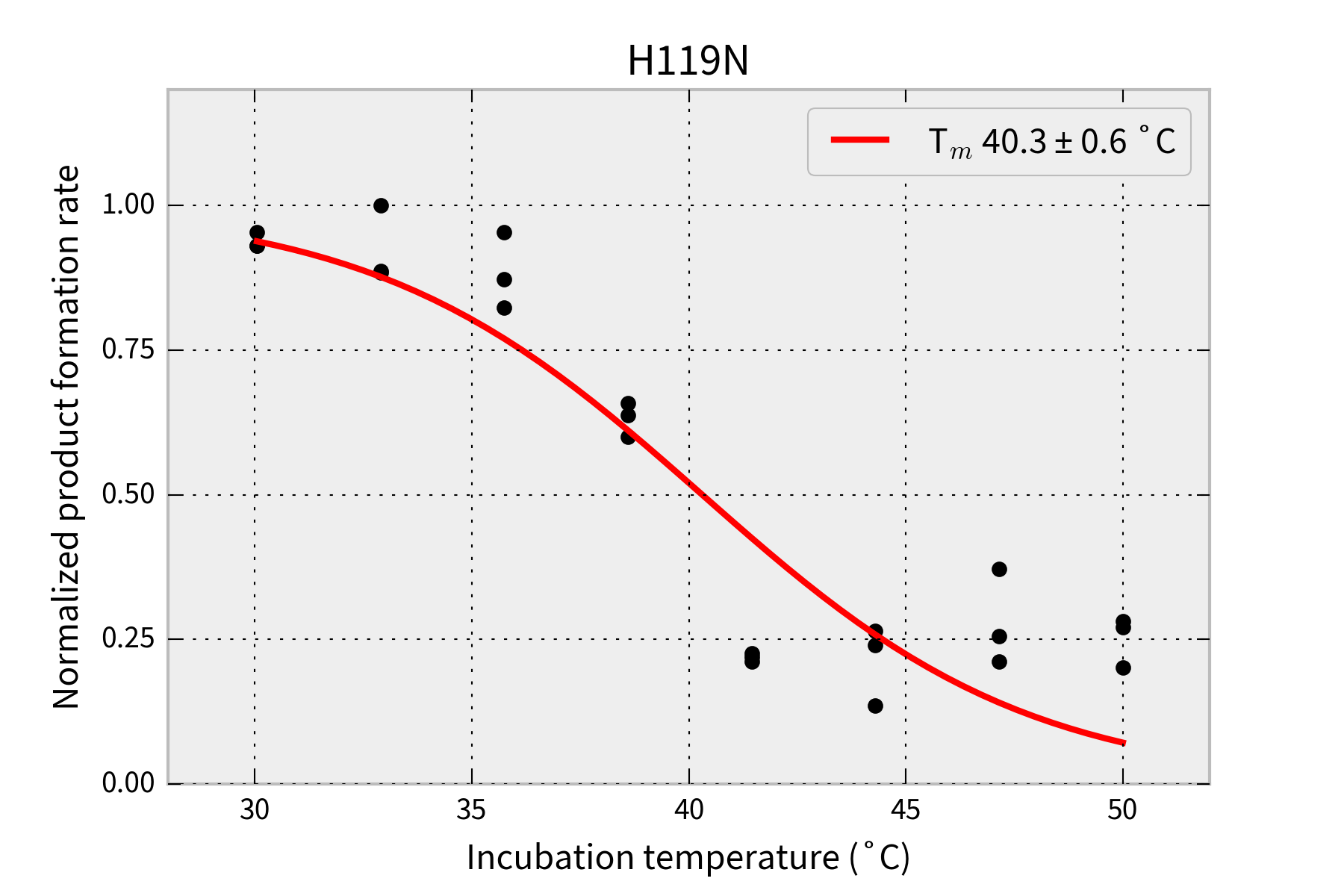

Supplement: S3 Figs — (ZIP) [file pone.0176255.s006.zip › S3 Figures/H119N.png]

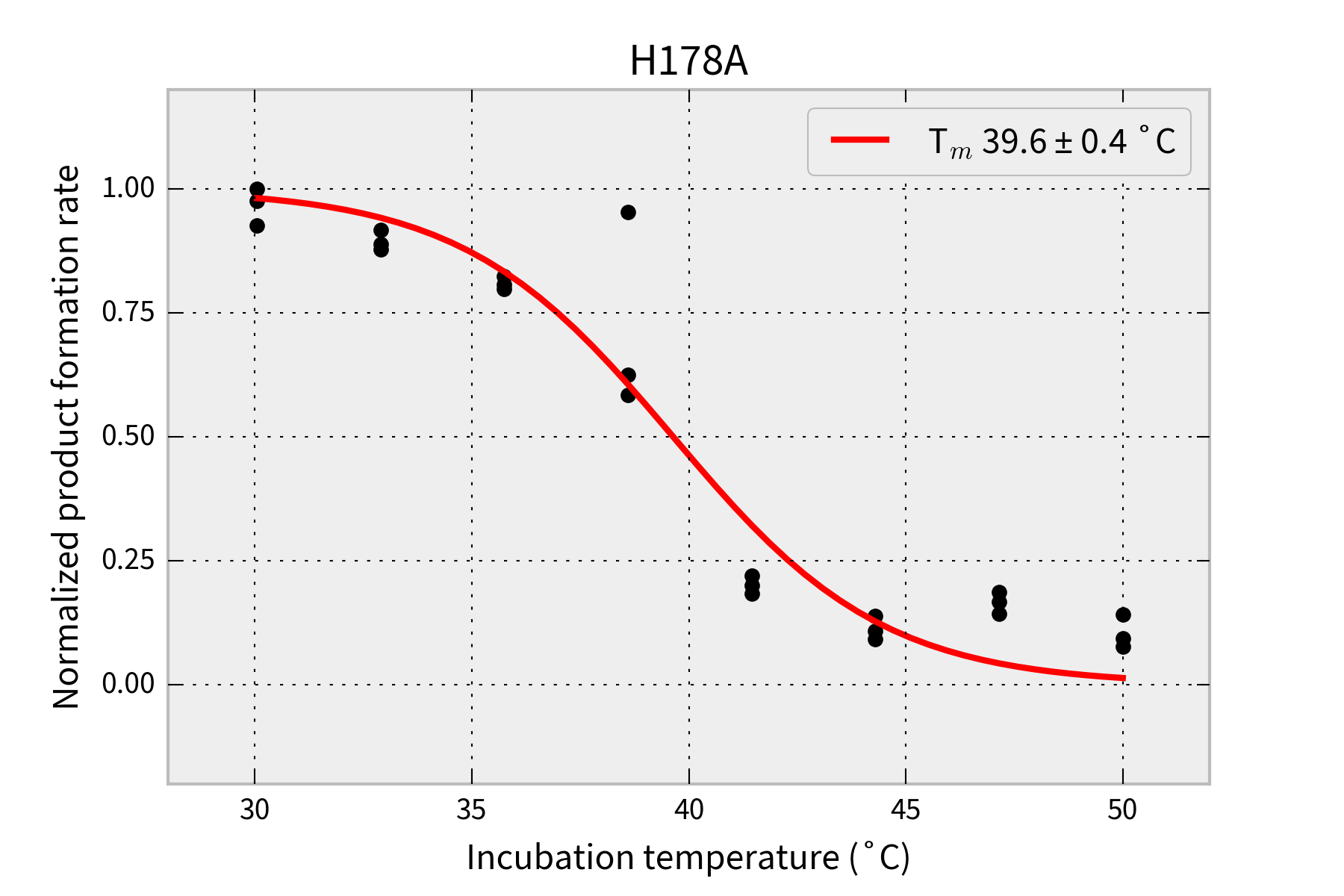

Supplement: S3 Figs — (ZIP) [file pone.0176255.s006.zip › S3 Figures/H178A.png]

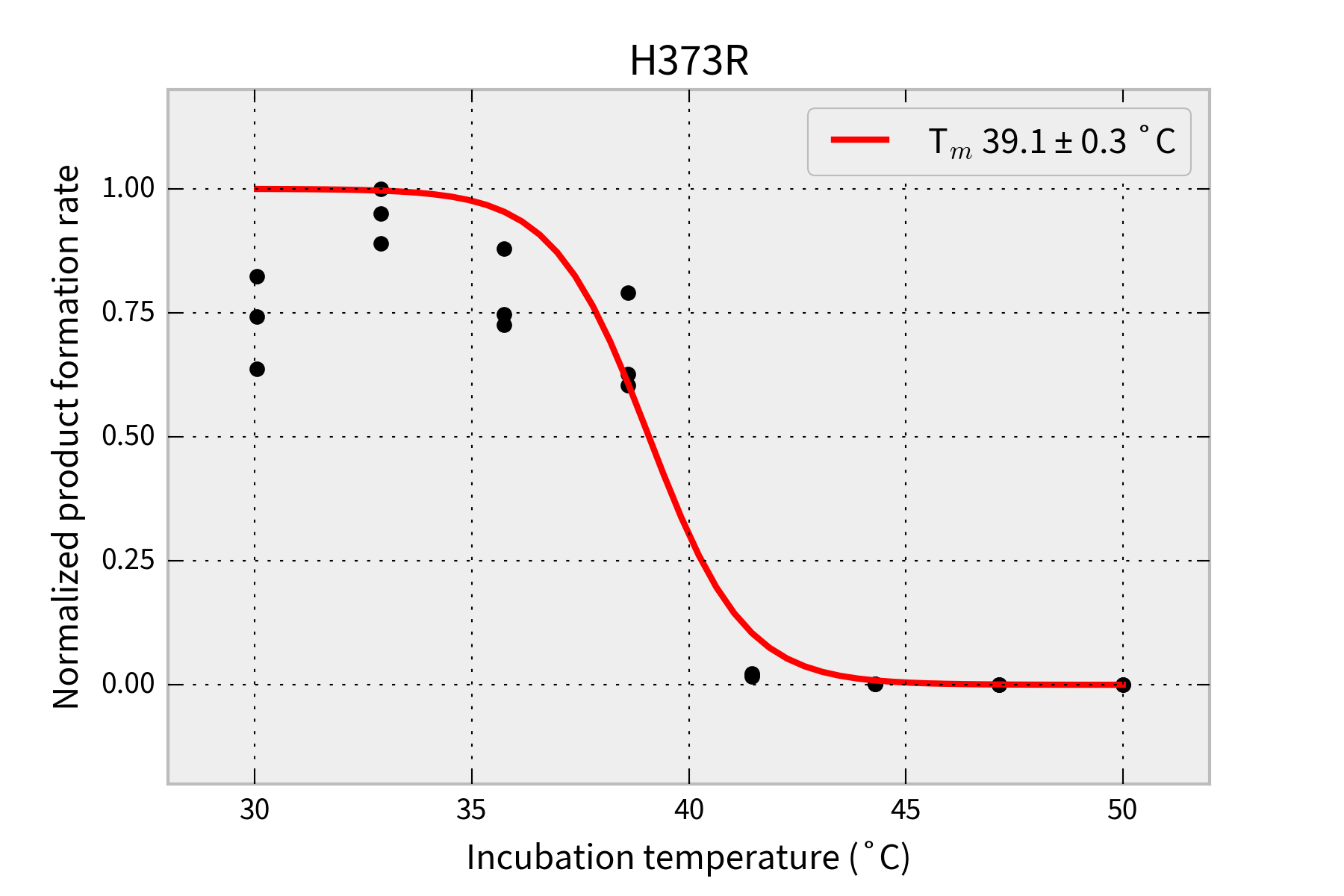

Supplement: S3 Figs — (ZIP) [file pone.0176255.s006.zip › S3 Figures/H373R.png]

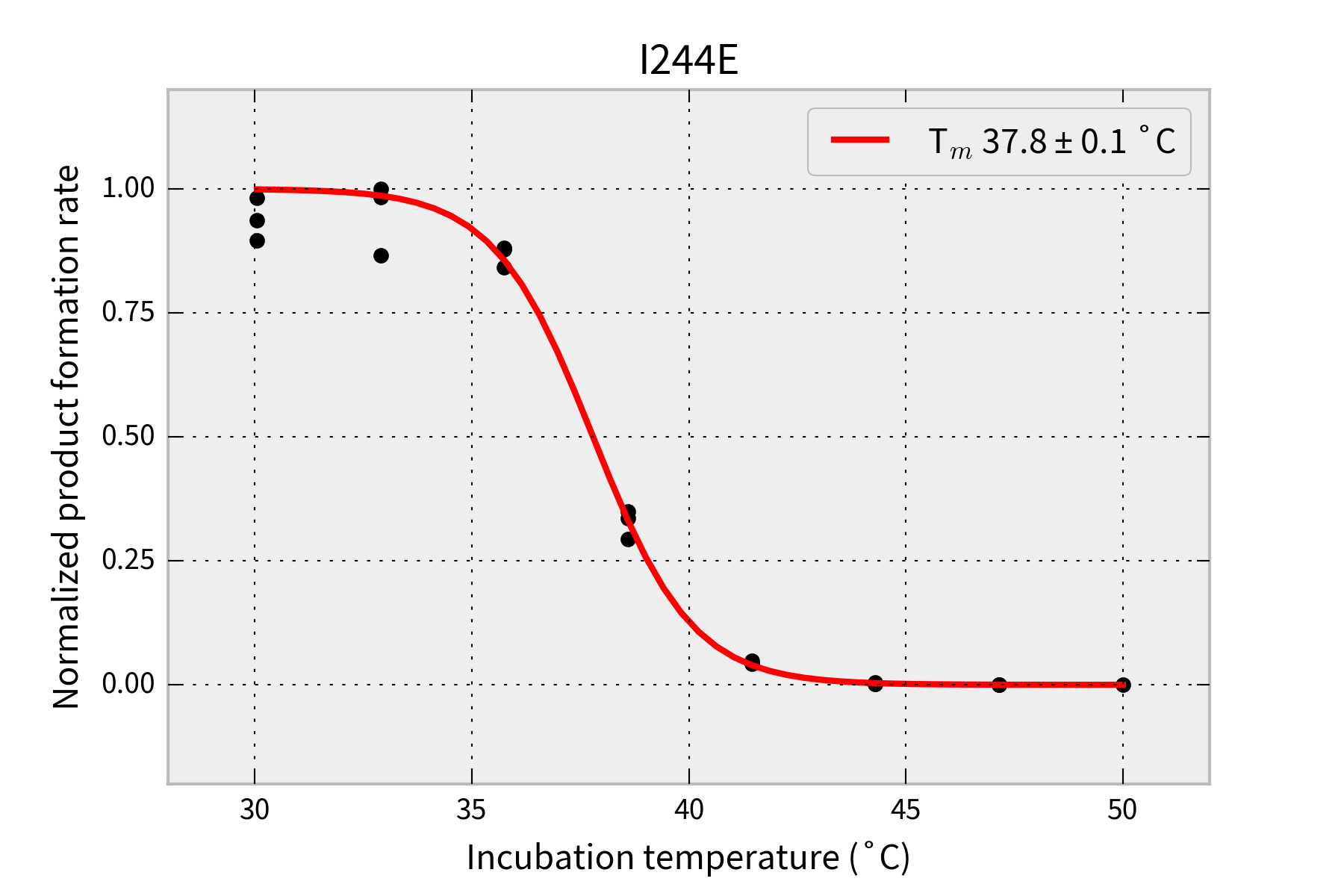

Supplement: S3 Figs — (ZIP) [file pone.0176255.s006.zip › S3 Figures/I244E.png]

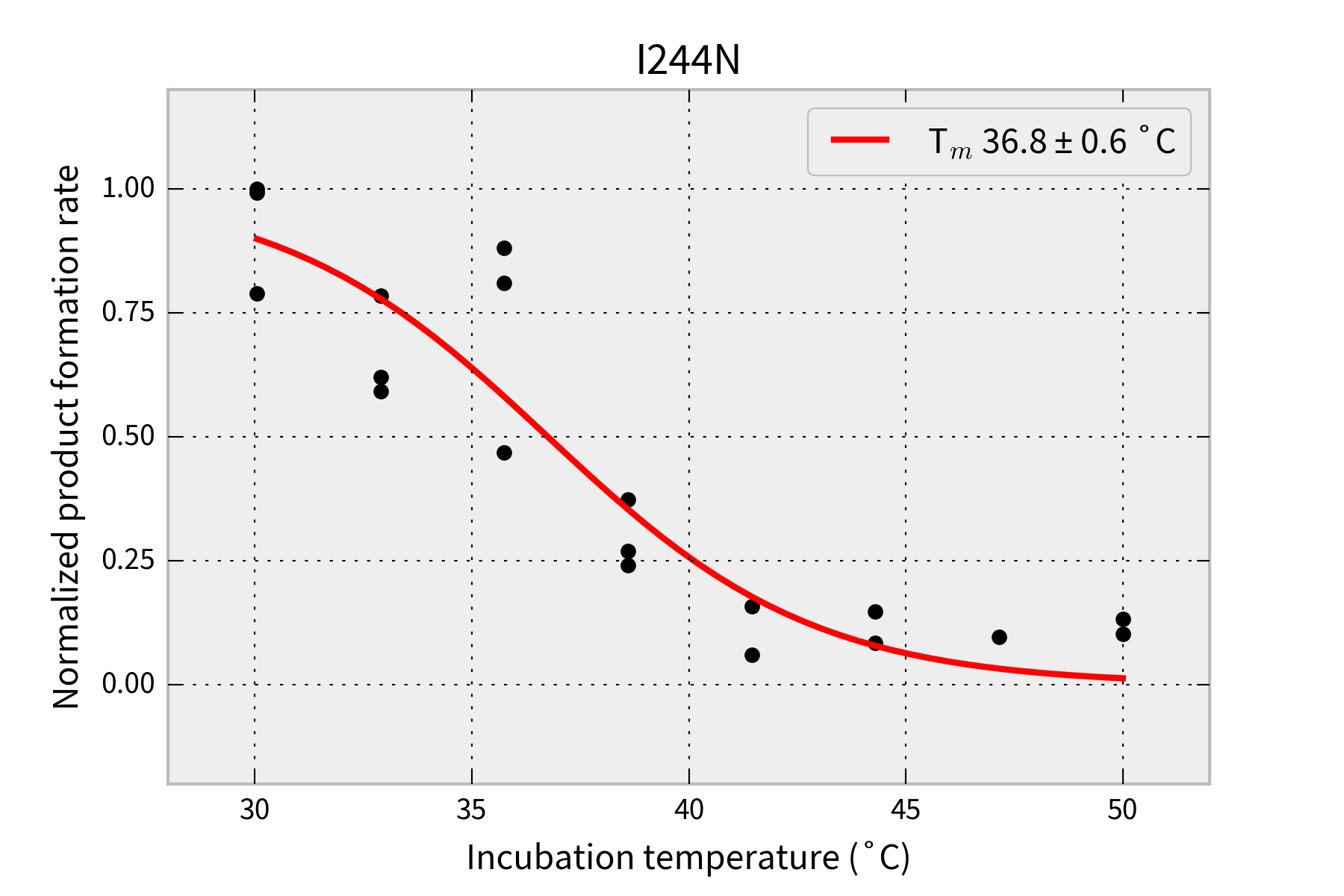

Supplement: S3 Figs — (ZIP) [file pone.0176255.s006.zip › S3 Figures/I244N.png]

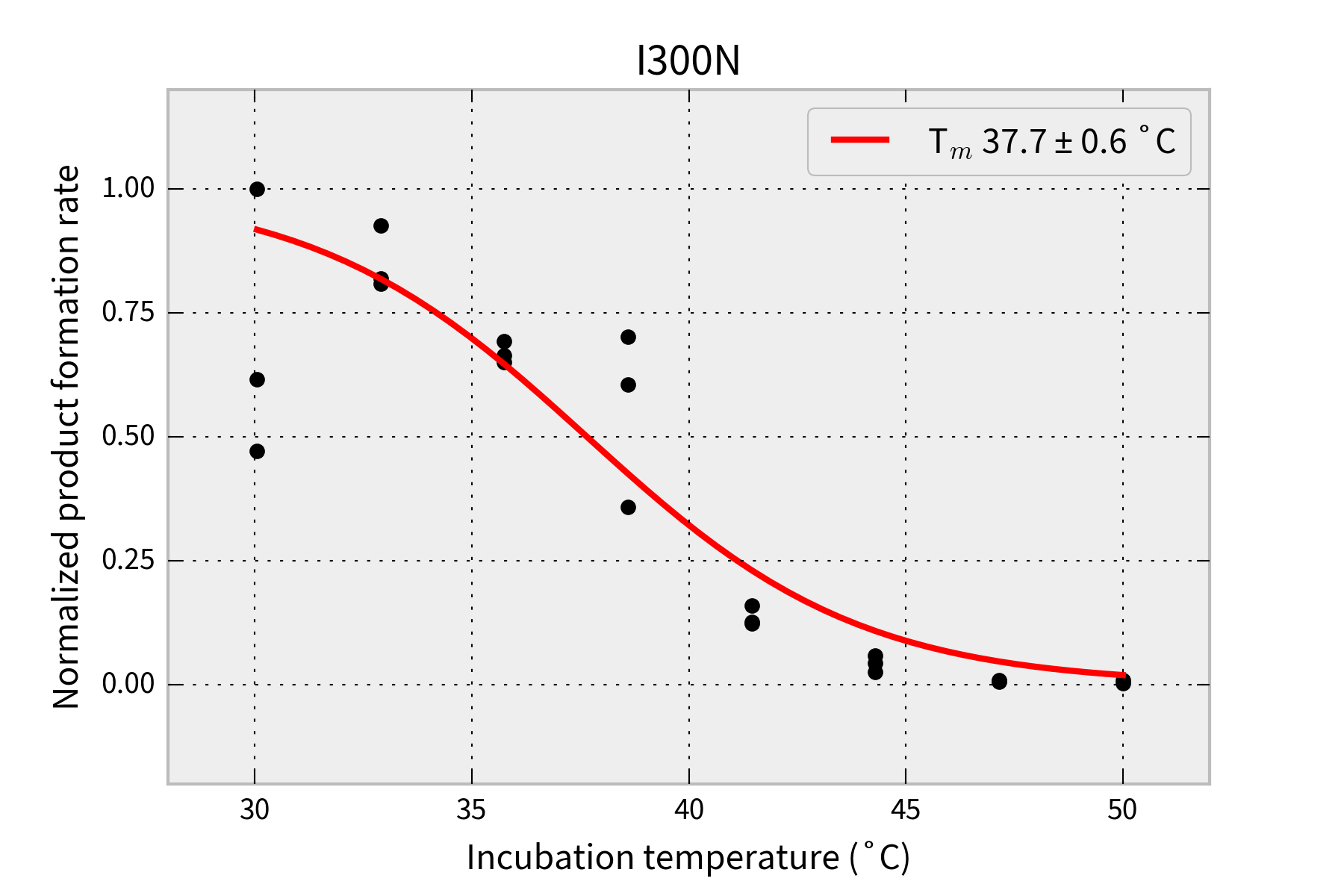

Supplement: S3 Figs — (ZIP) [file pone.0176255.s006.zip › S3 Figures/I300N.png]

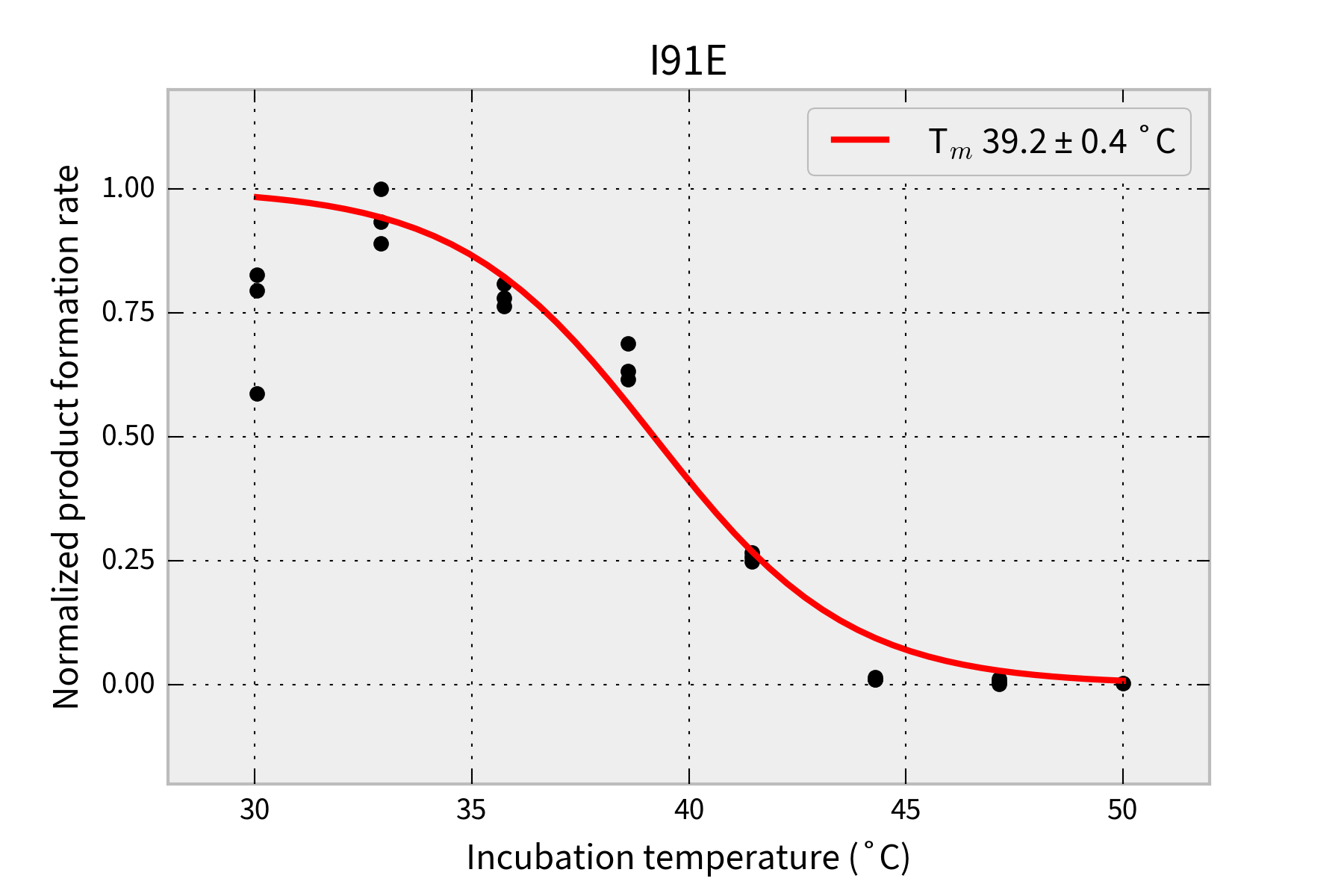

Supplement: S3 Figs — (ZIP) [file pone.0176255.s006.zip › S3 Figures/I91E.png]

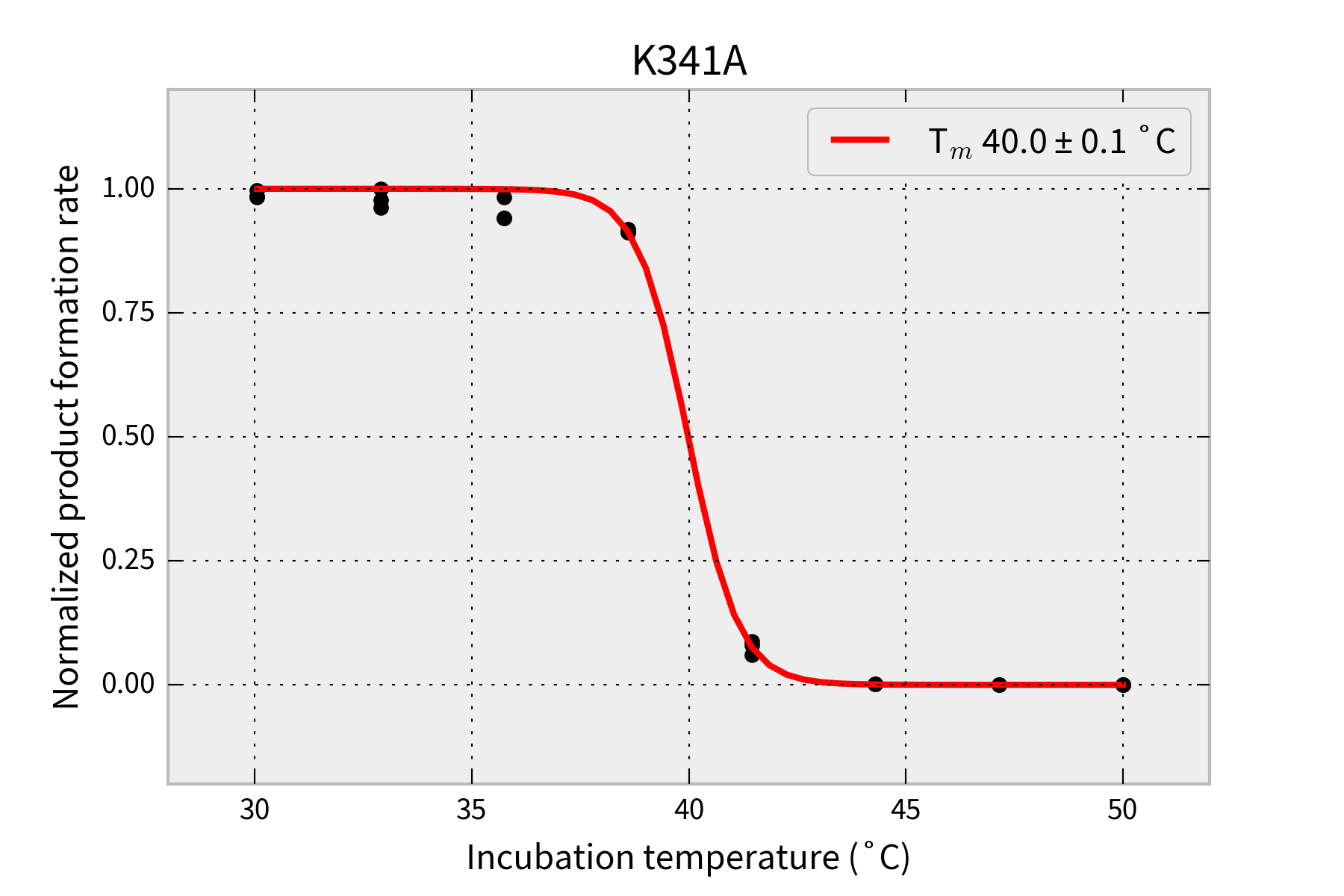

Supplement: S3 Figs — (ZIP) [file pone.0176255.s006.zip › S3 Figures/K341A.png]

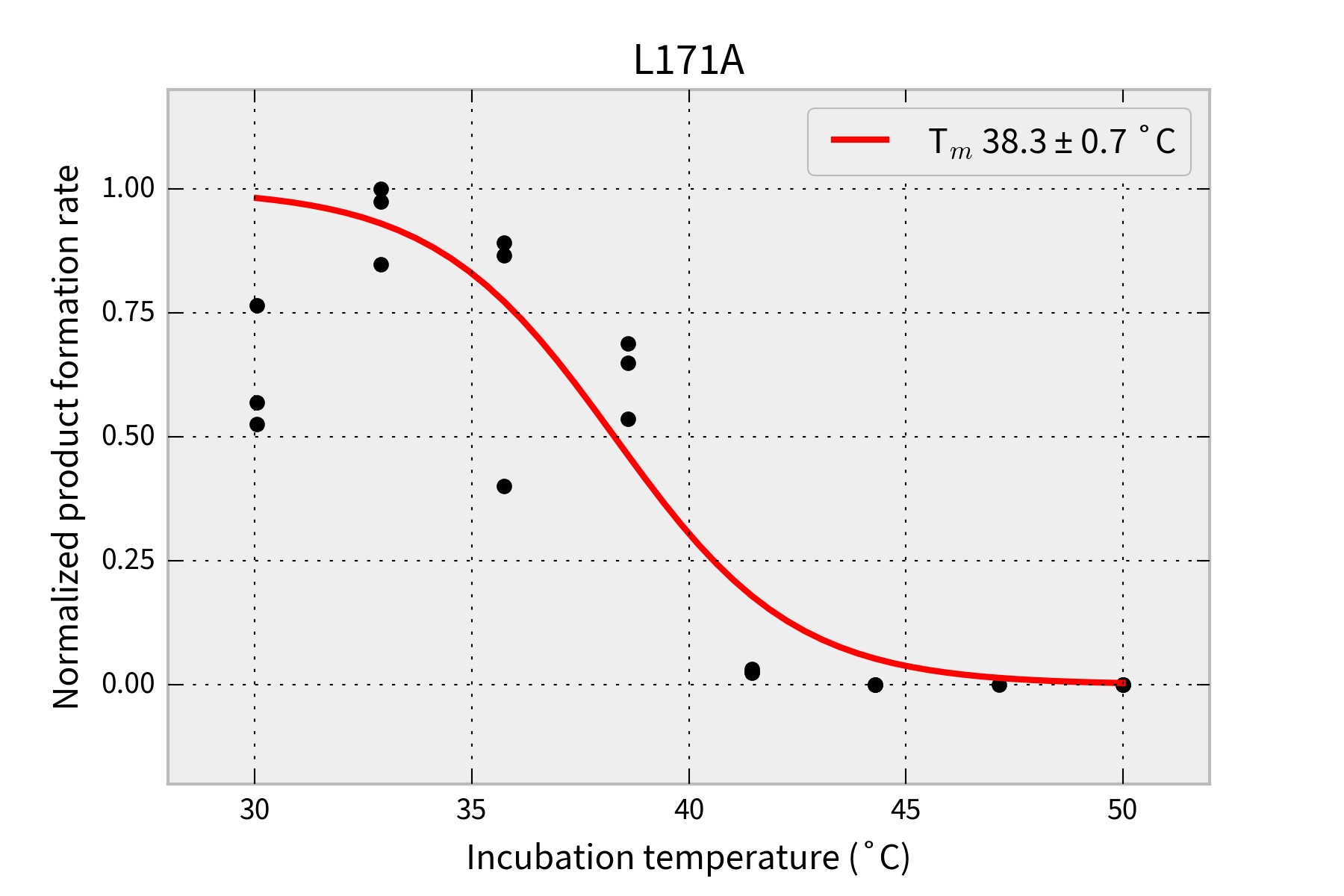

Supplement: S3 Figs — (ZIP) [file pone.0176255.s006.zip › S3 Figures/L171A.png]

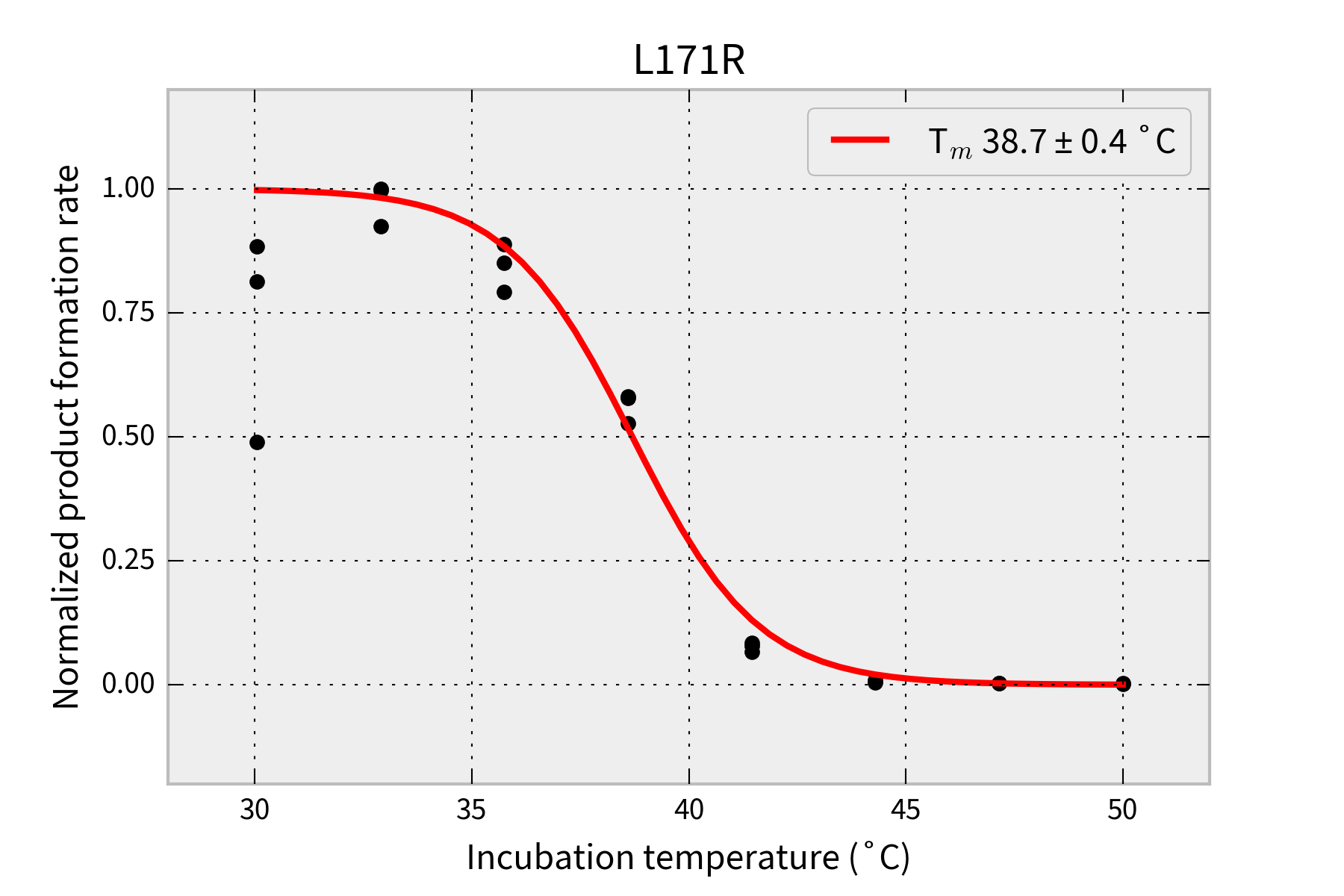

Supplement: S3 Figs — (ZIP) [file pone.0176255.s006.zip › S3 Figures/L171R.png]

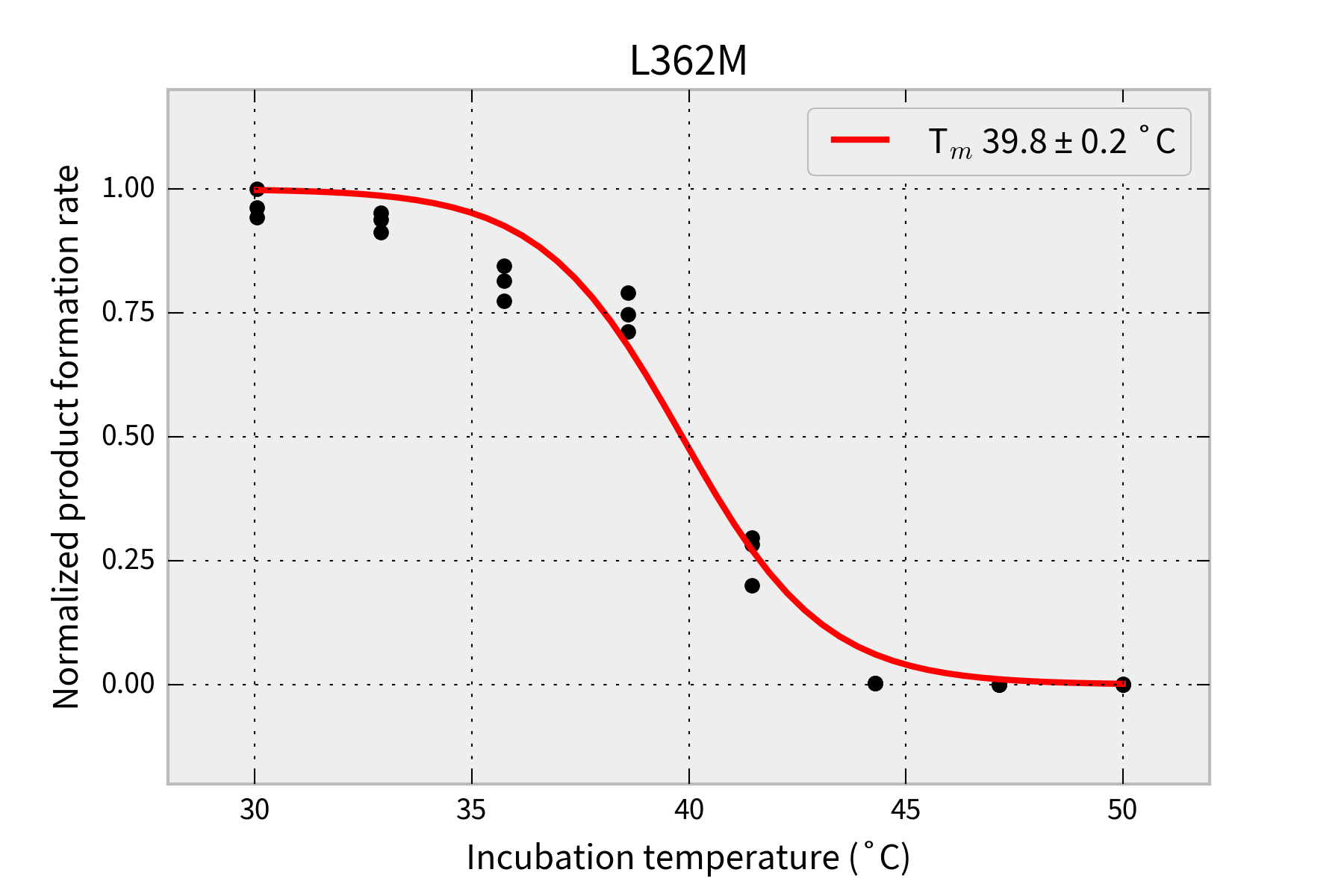

Supplement: S3 Figs — (ZIP) [file pone.0176255.s006.zip › S3 Figures/L362M.png]

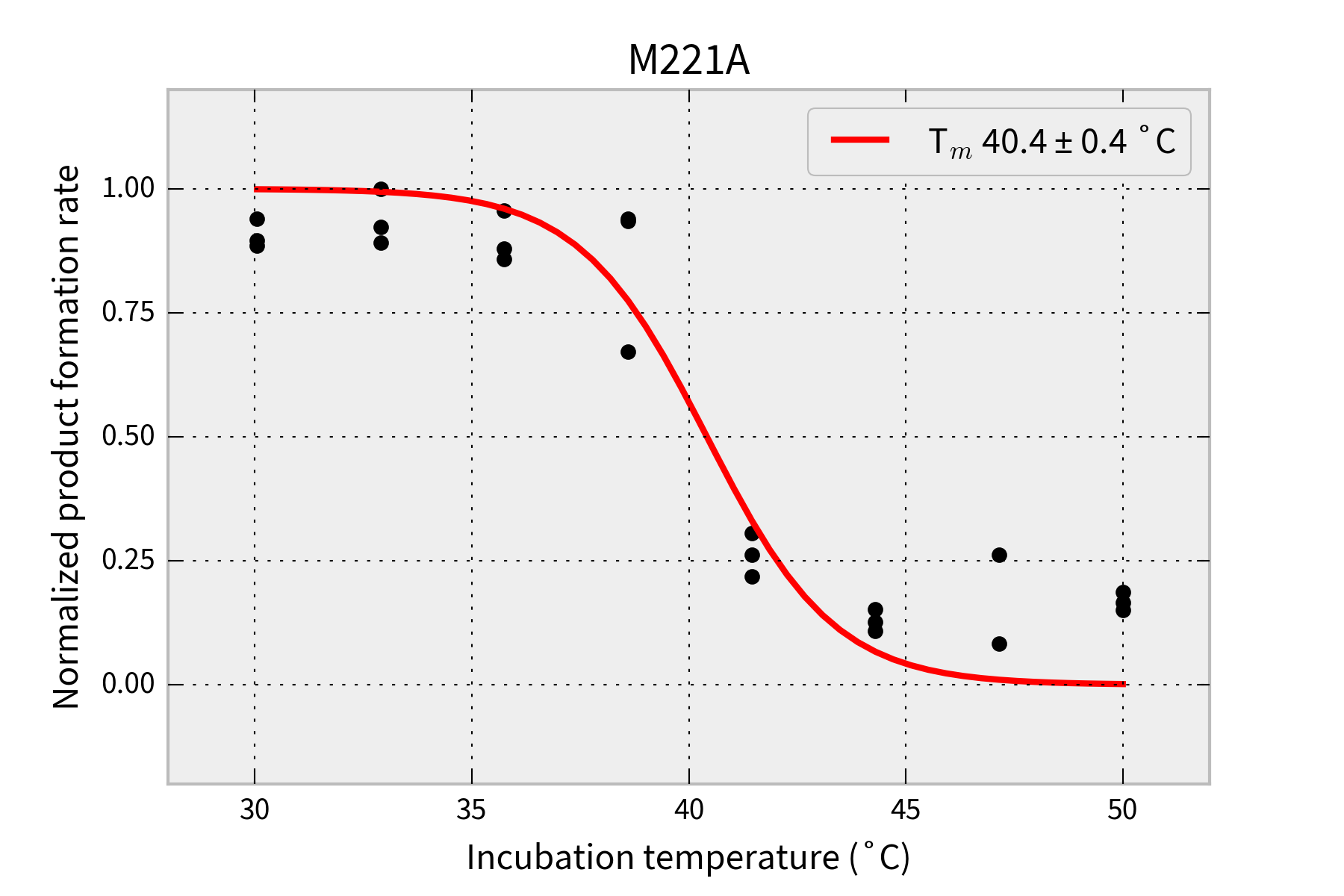

Supplement: S3 Figs — (ZIP) [file pone.0176255.s006.zip › S3 Figures/M221A.png]

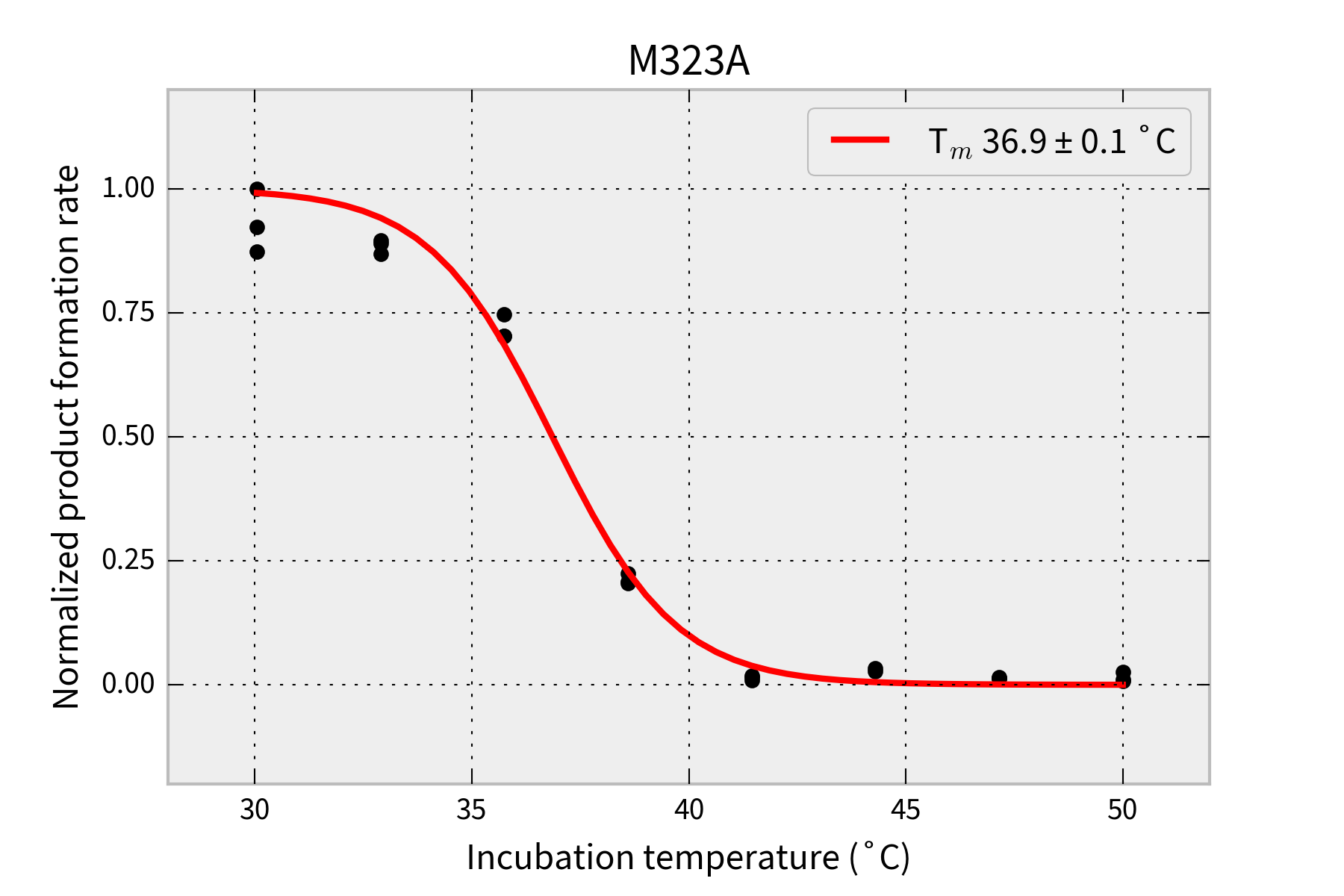

Supplement: S3 Figs — (ZIP) [file pone.0176255.s006.zip › S3 Figures/M323A.png]

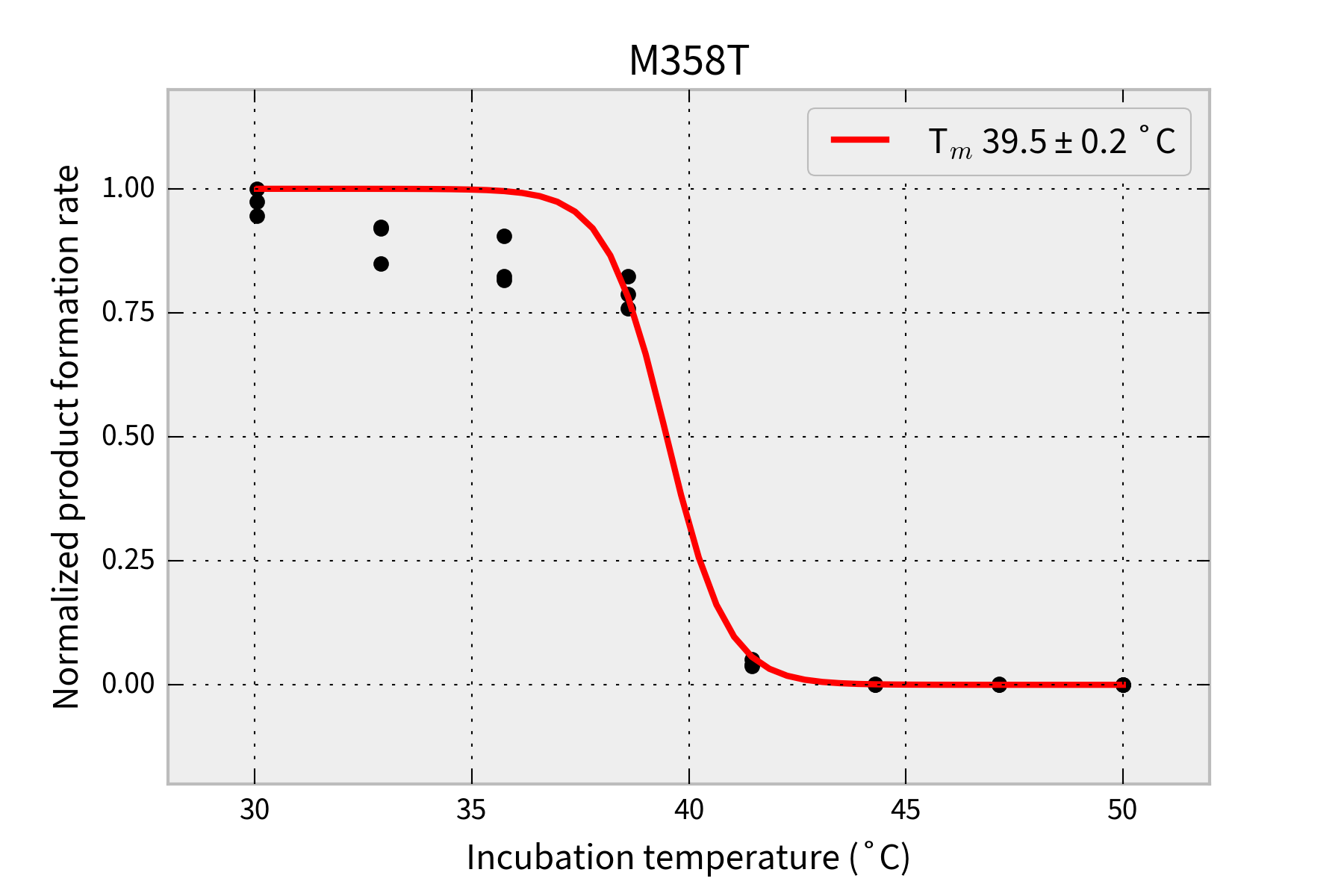

Supplement: S3 Figs — (ZIP) [file pone.0176255.s006.zip › S3 Figures/M358T.png]

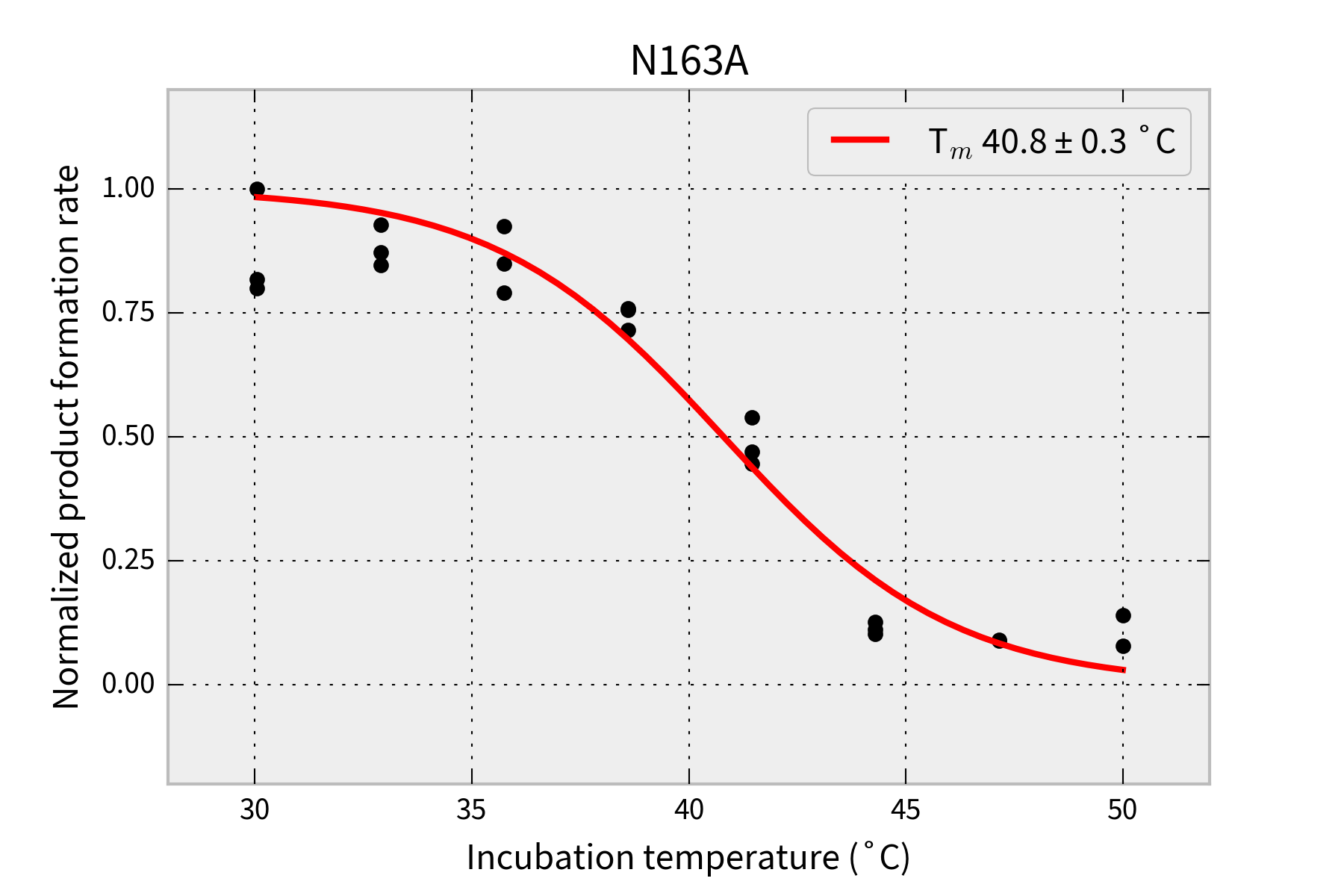

Supplement: S3 Figs — (ZIP) [file pone.0176255.s006.zip › S3 Figures/N163A.png]

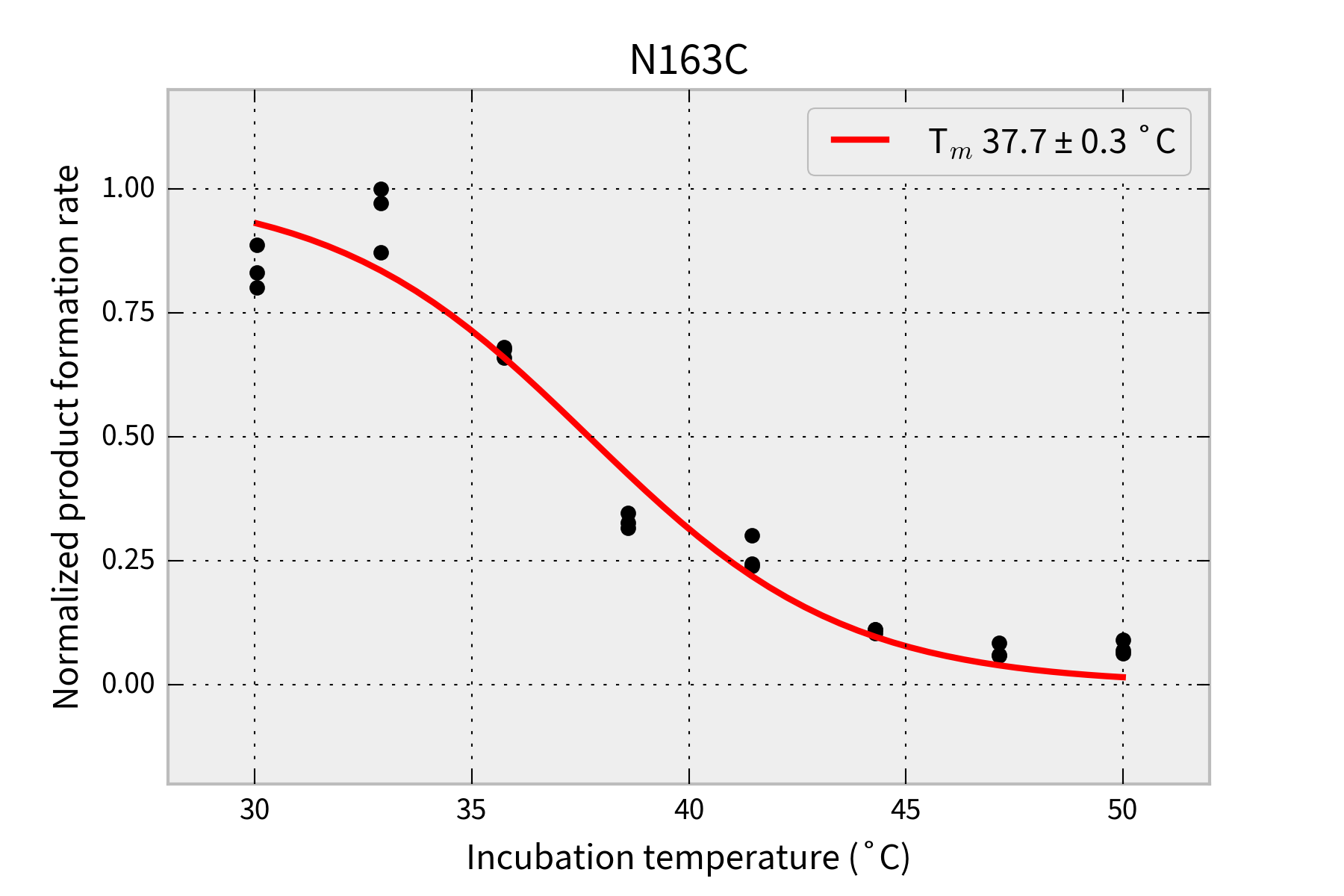

Supplement: S3 Figs — (ZIP) [file pone.0176255.s006.zip › S3 Figures/N163C.png]

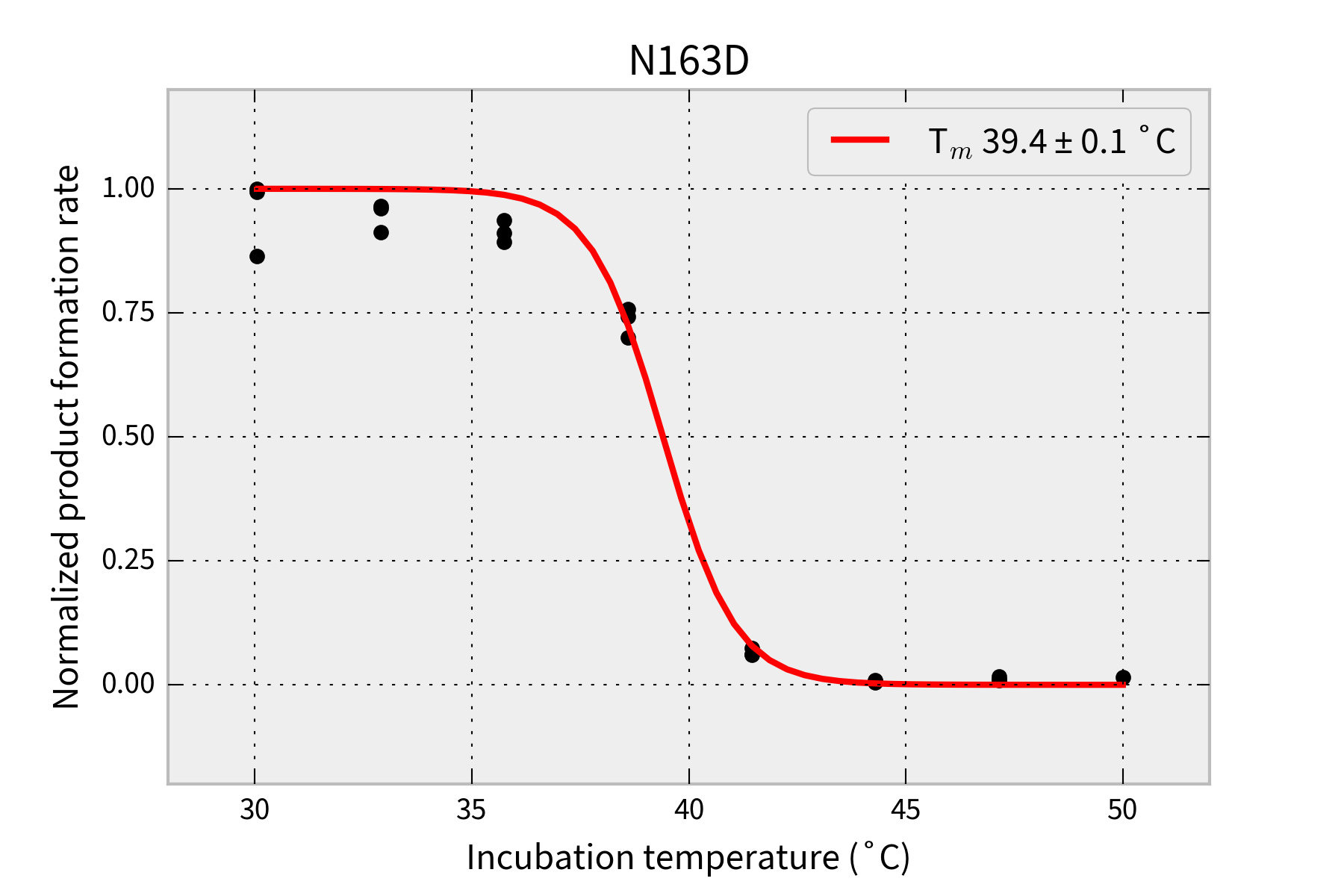

Supplement: S3 Figs — (ZIP) [file pone.0176255.s006.zip › S3 Figures/N163D.png]

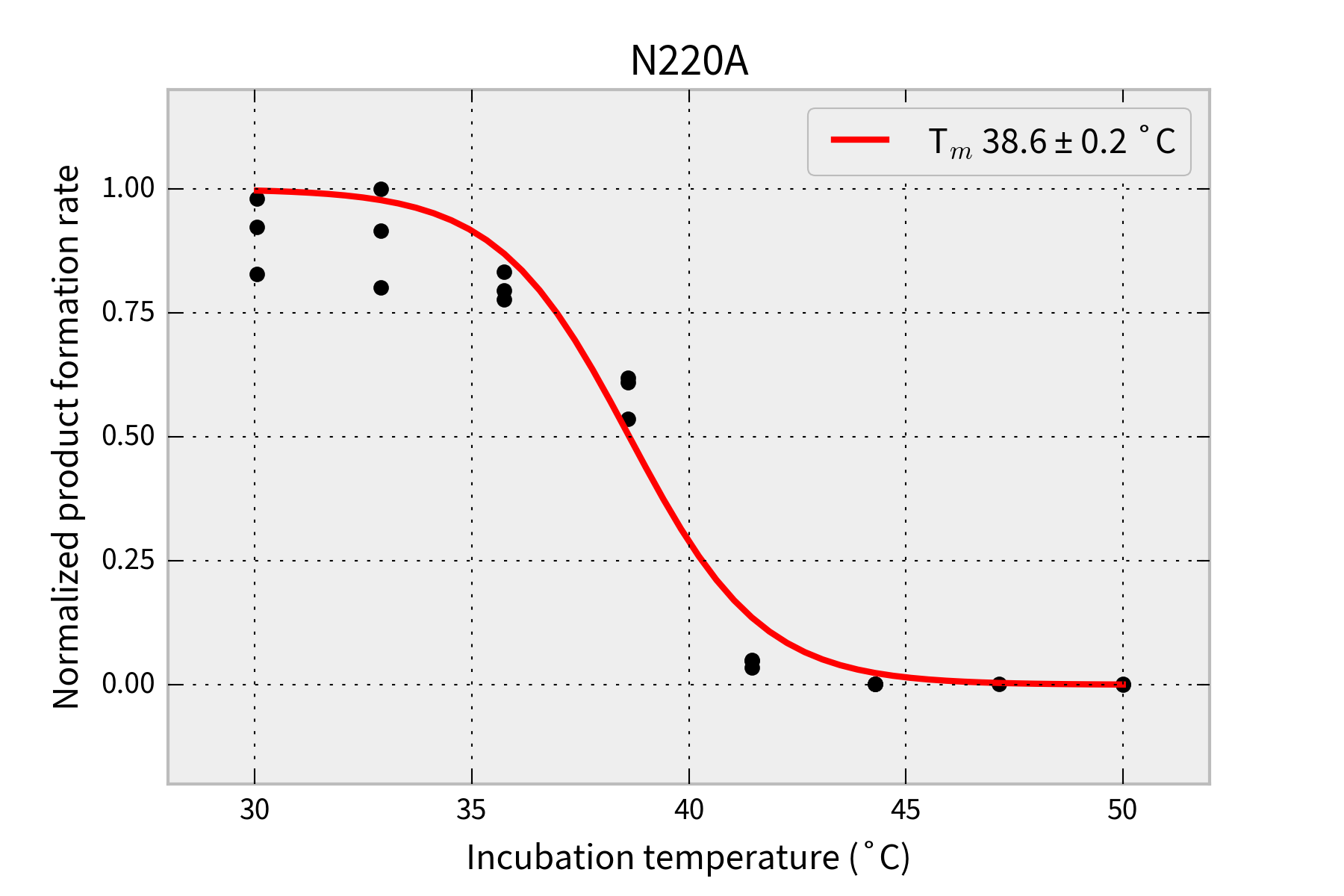

Supplement: S3 Figs — (ZIP) [file pone.0176255.s006.zip › S3 Figures/N220A.png]

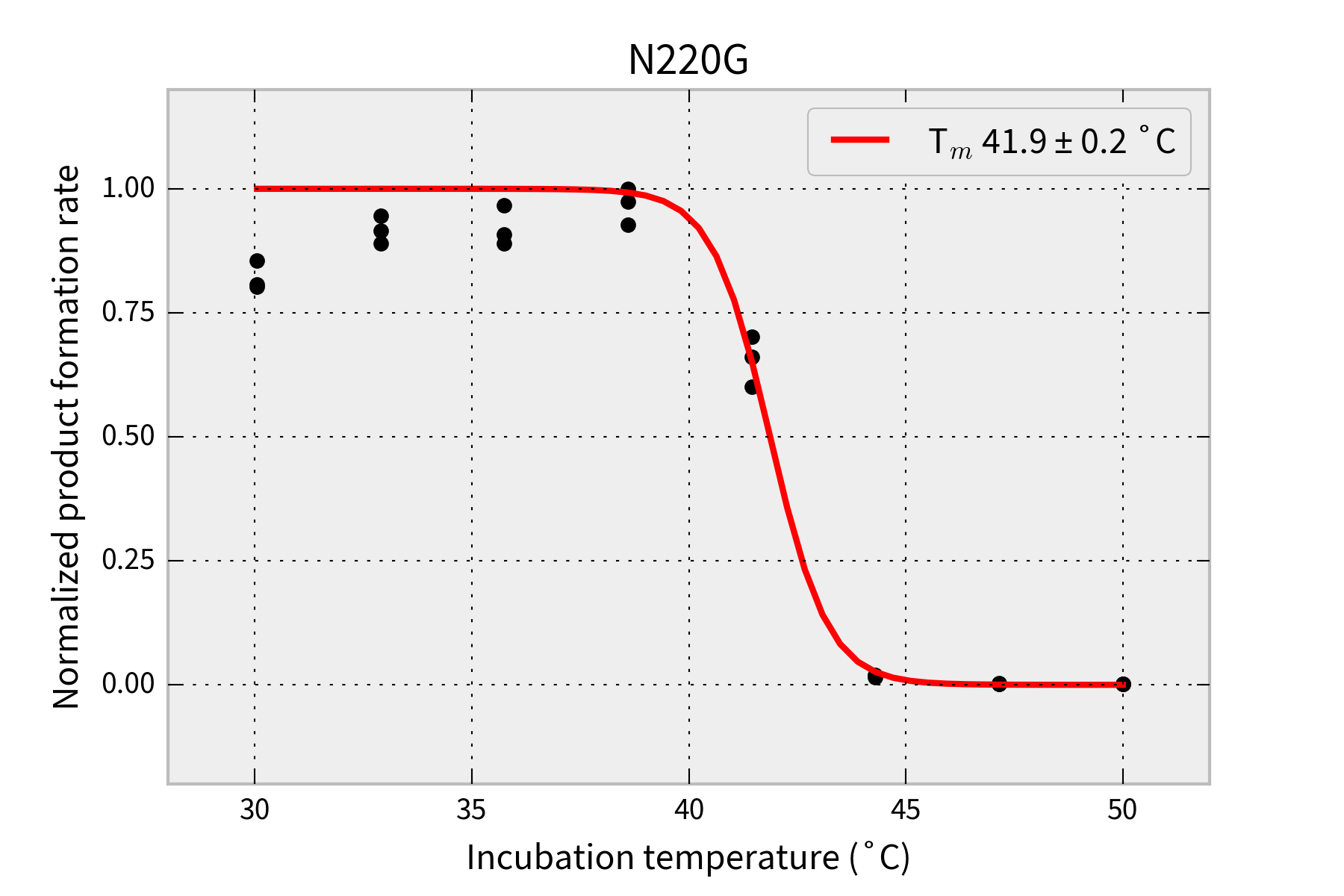

Supplement: S3 Figs — (ZIP) [file pone.0176255.s006.zip › S3 Figures/N220G.png]

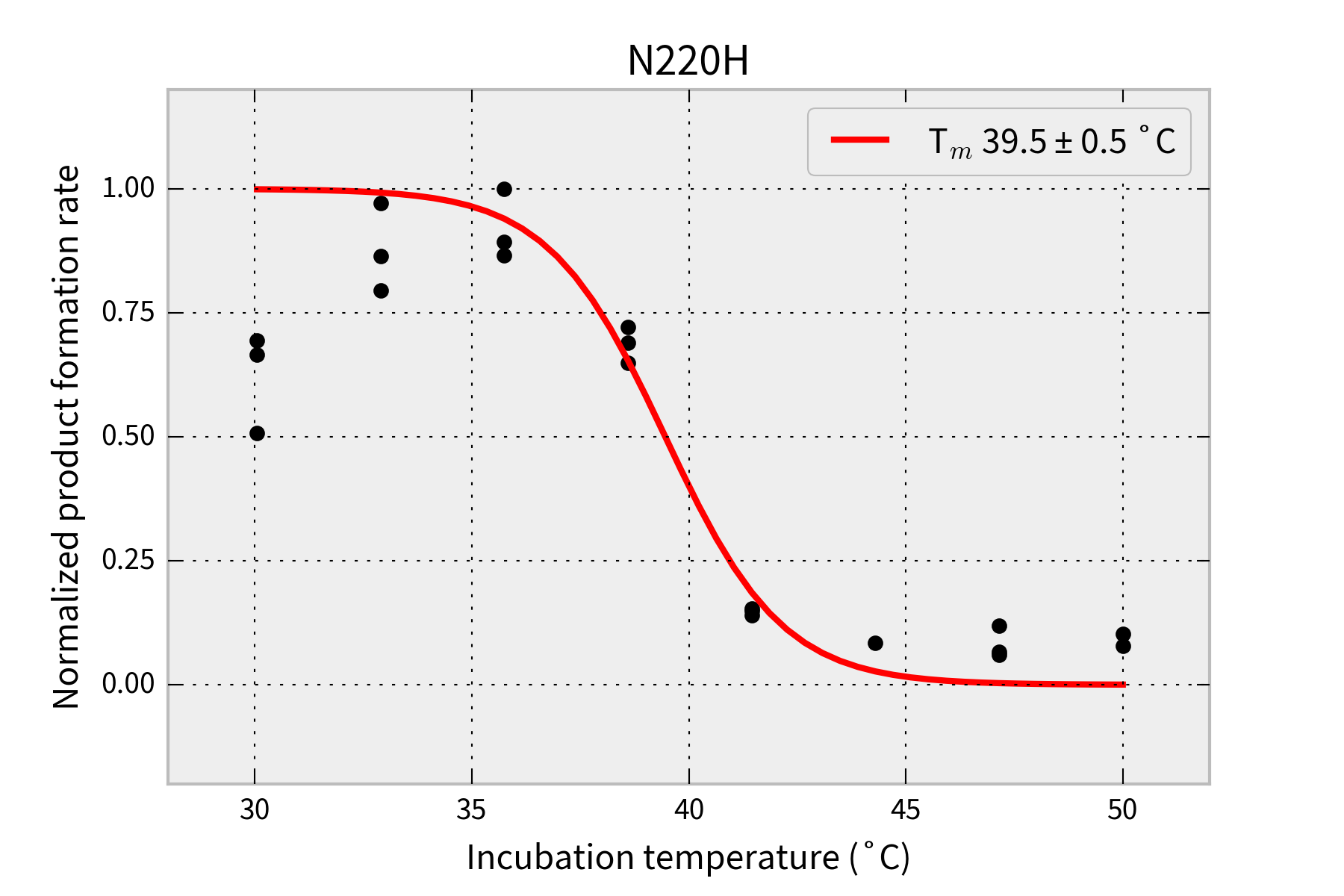

Supplement: S3 Figs — (ZIP) [file pone.0176255.s006.zip › S3 Figures/N220H.png]

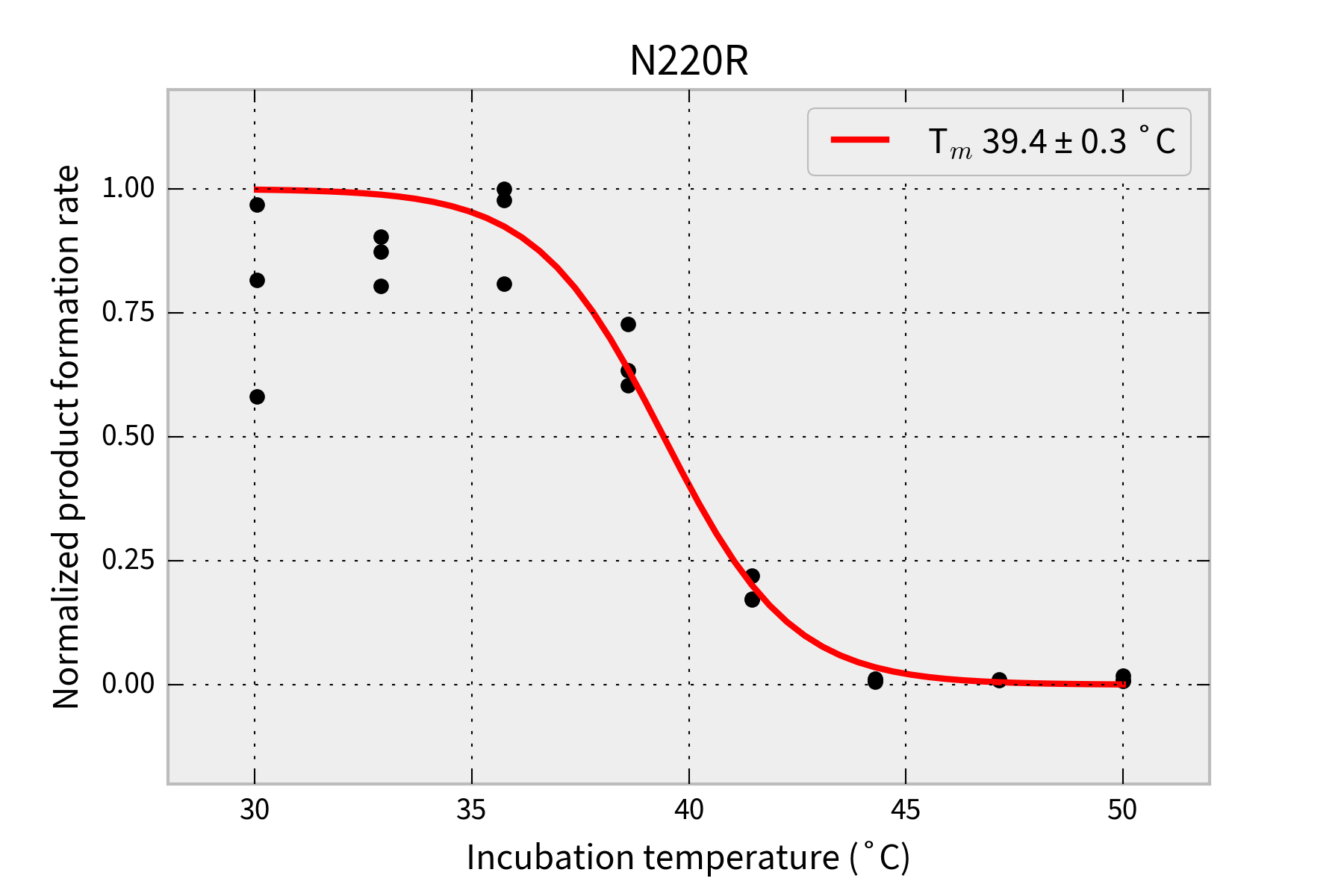

Supplement: S3 Figs — (ZIP) [file pone.0176255.s006.zip › S3 Figures/N220R.png]

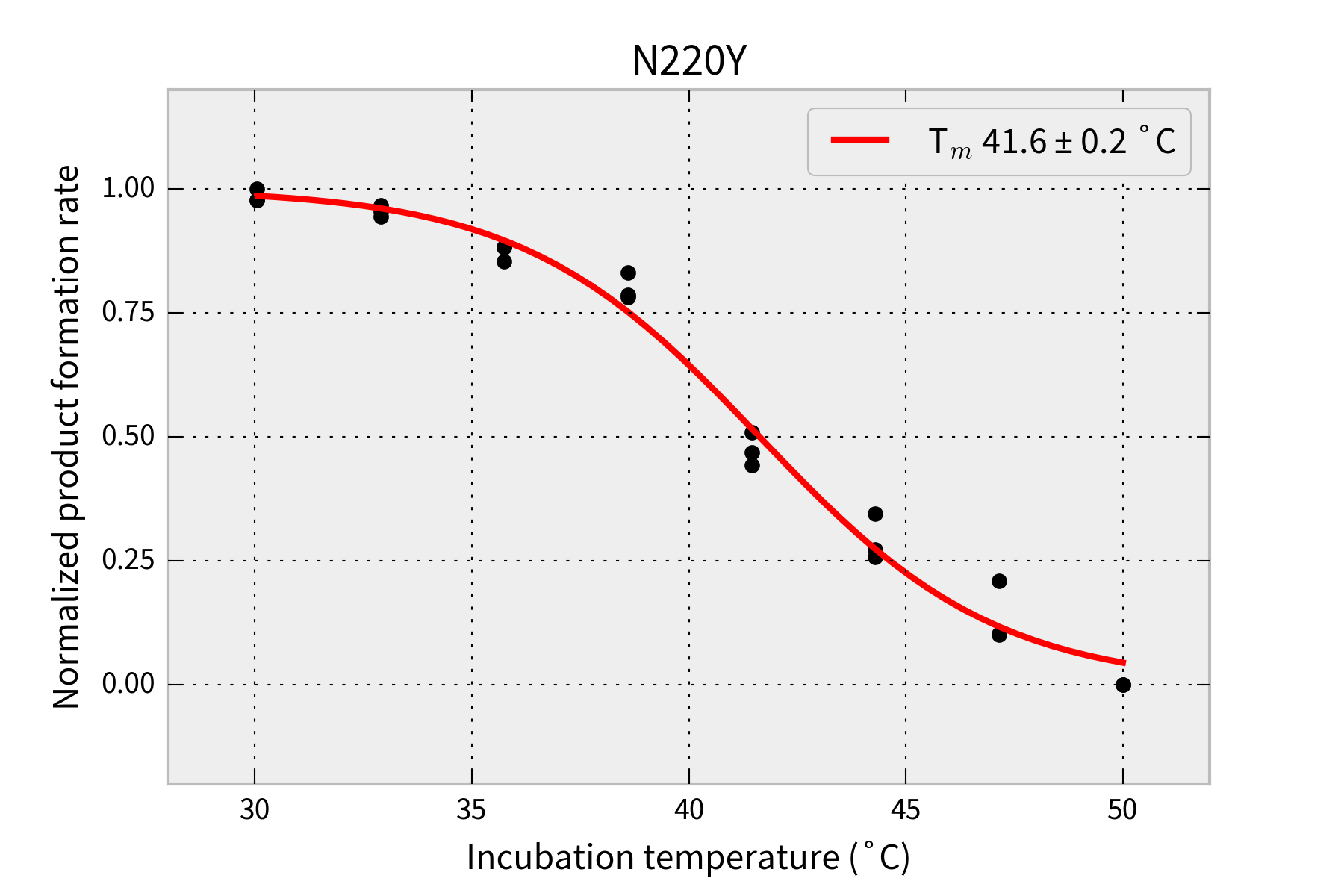

Supplement: S3 Figs — (ZIP) [file pone.0176255.s006.zip › S3 Figures/N220Y.png]

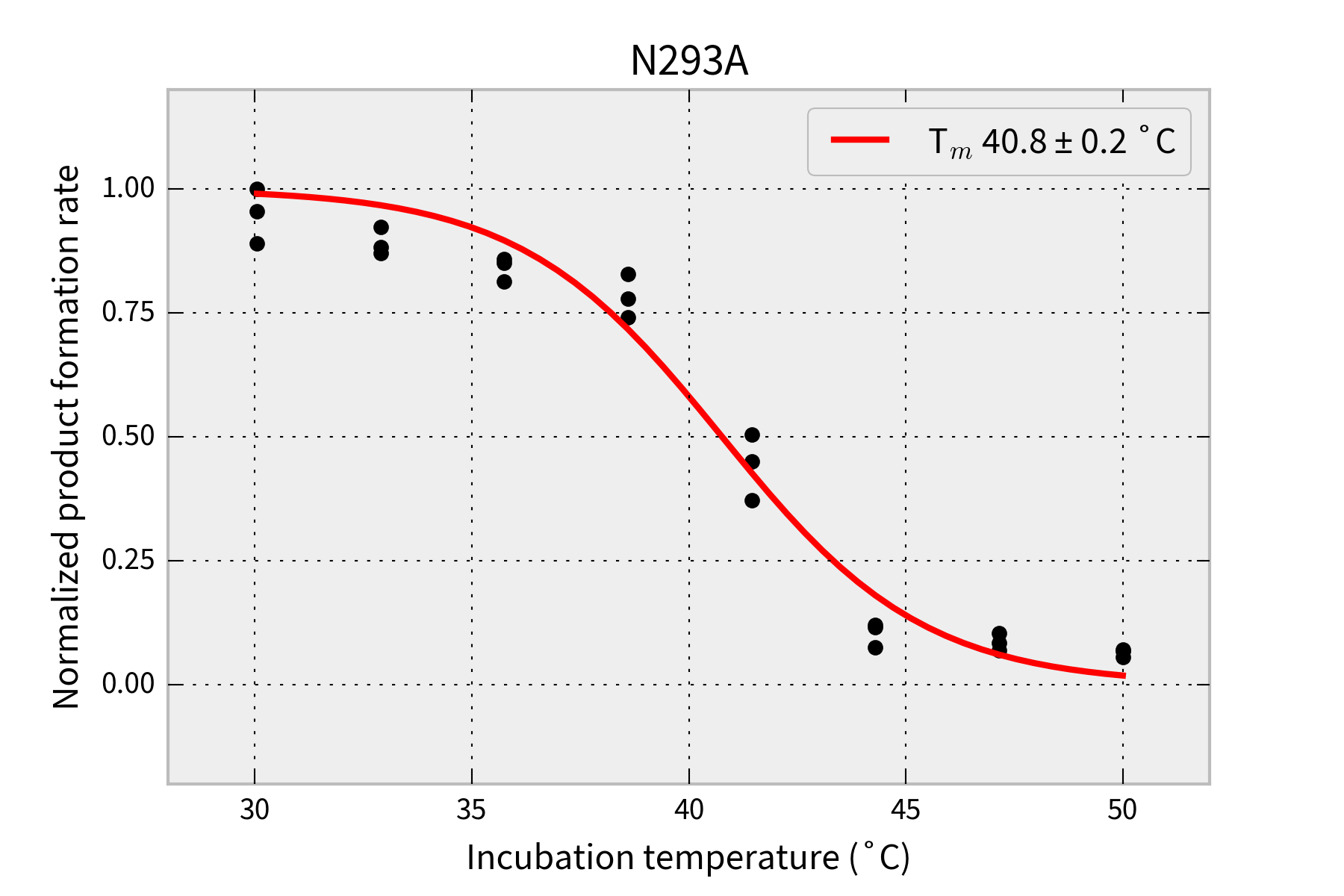

Supplement: S3 Figs — (ZIP) [file pone.0176255.s006.zip › S3 Figures/N293A.png]

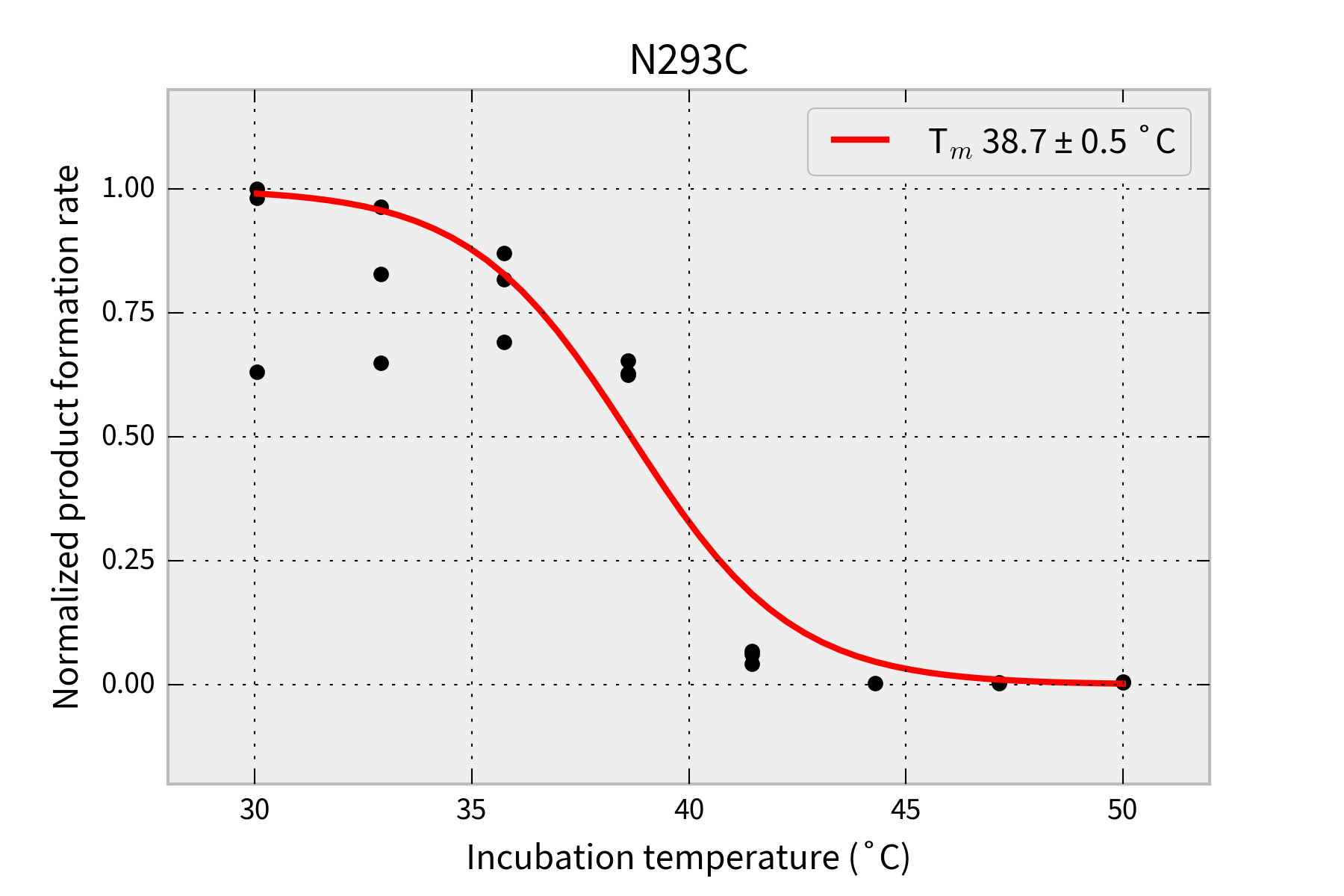

Supplement: S3 Figs — (ZIP) [file pone.0176255.s006.zip › S3 Figures/N293C.png]

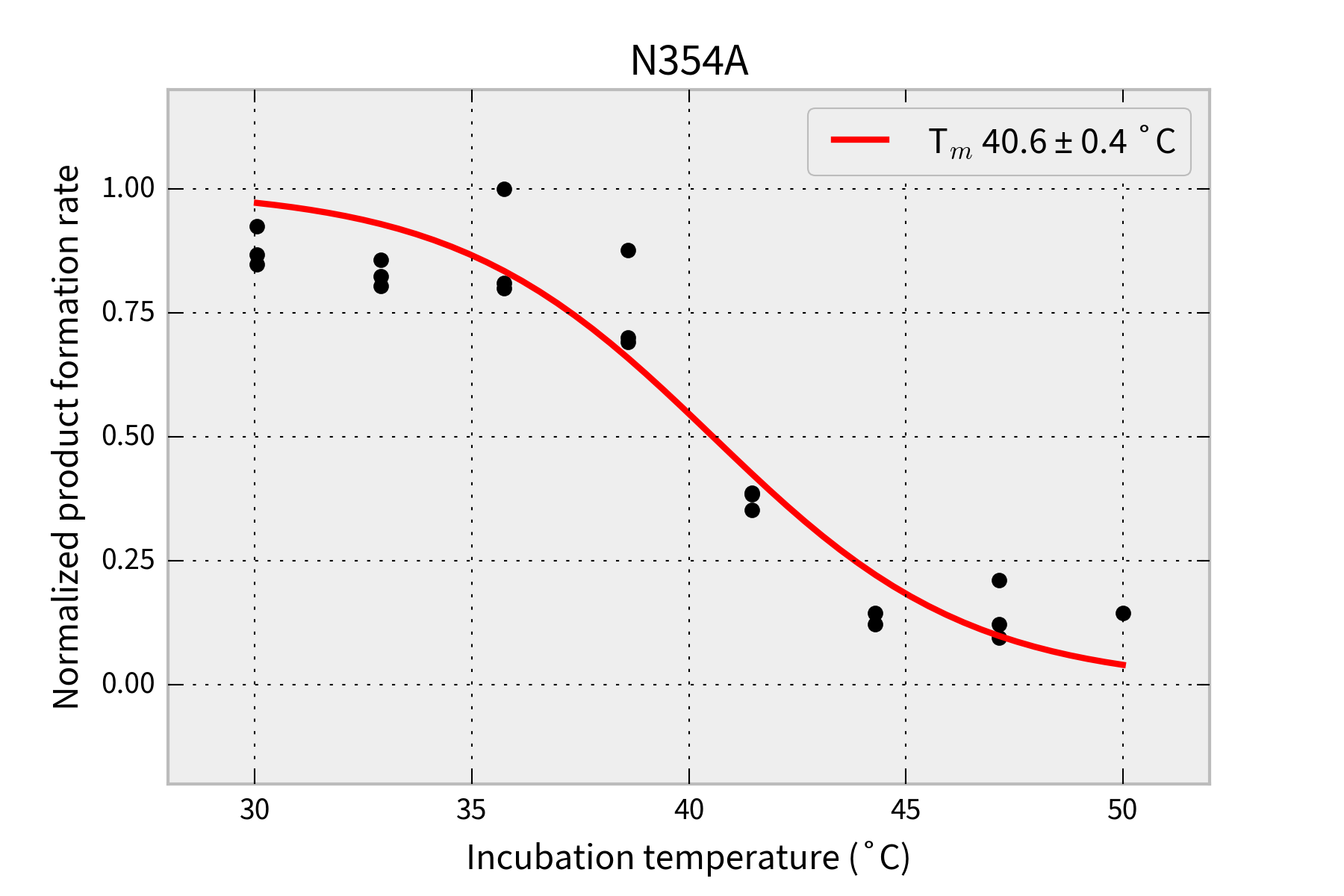

Supplement: S3 Figs — (ZIP) [file pone.0176255.s006.zip › S3 Figures/N354A.png]

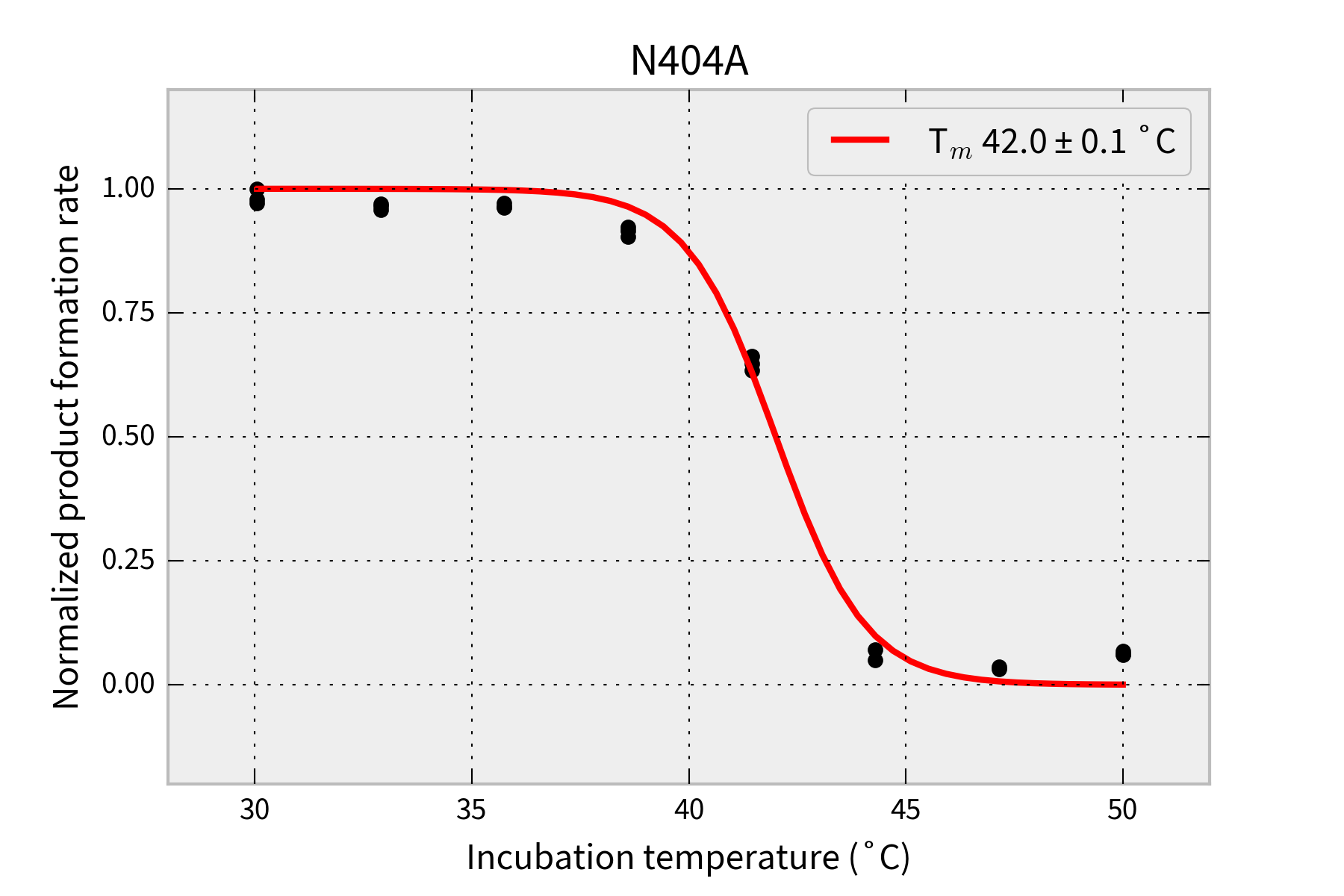

Supplement: S3 Figs — (ZIP) [file pone.0176255.s006.zip › S3 Figures/N404A.png]

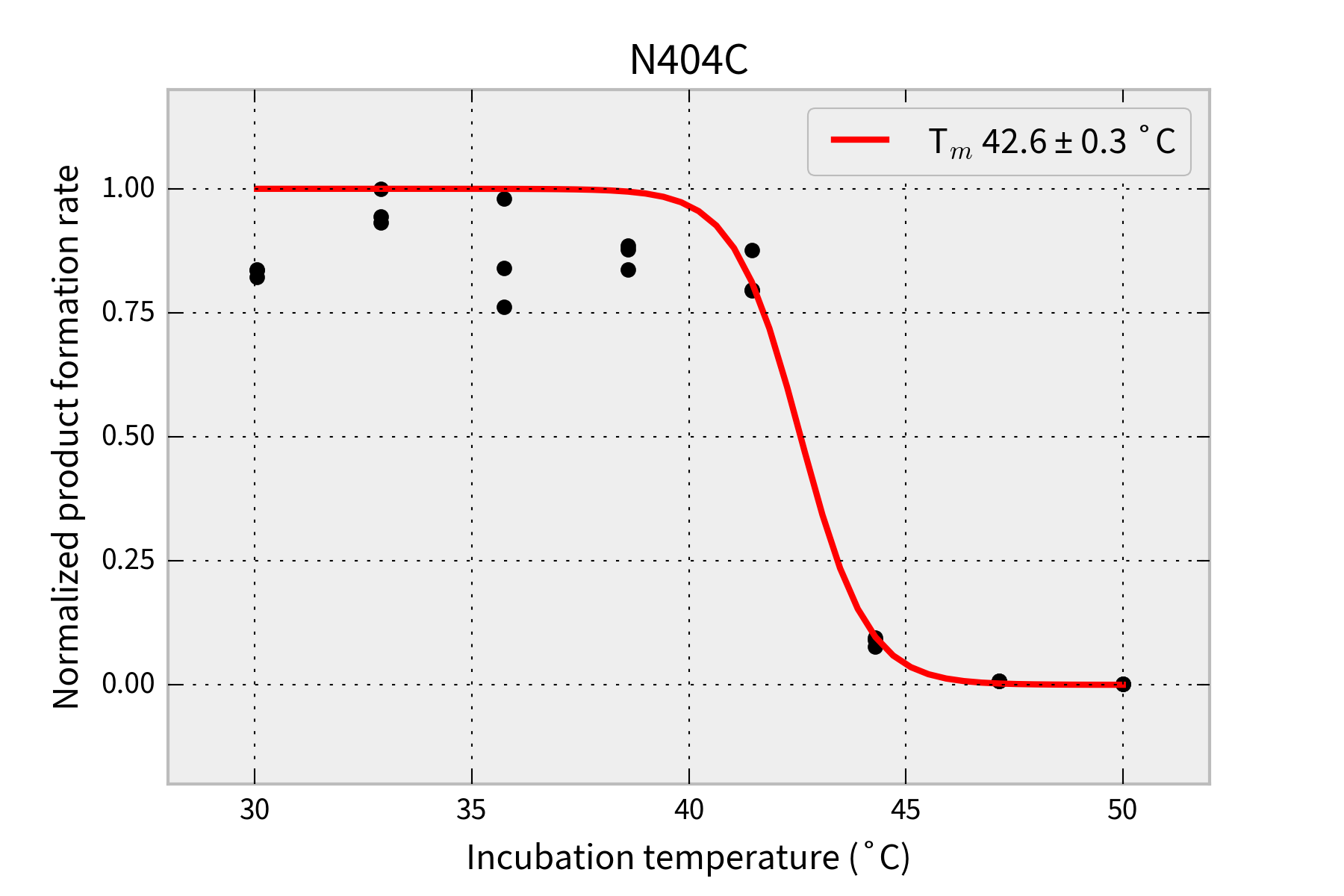

Supplement: S3 Figs — (ZIP) [file pone.0176255.s006.zip › S3 Figures/N404C.png]

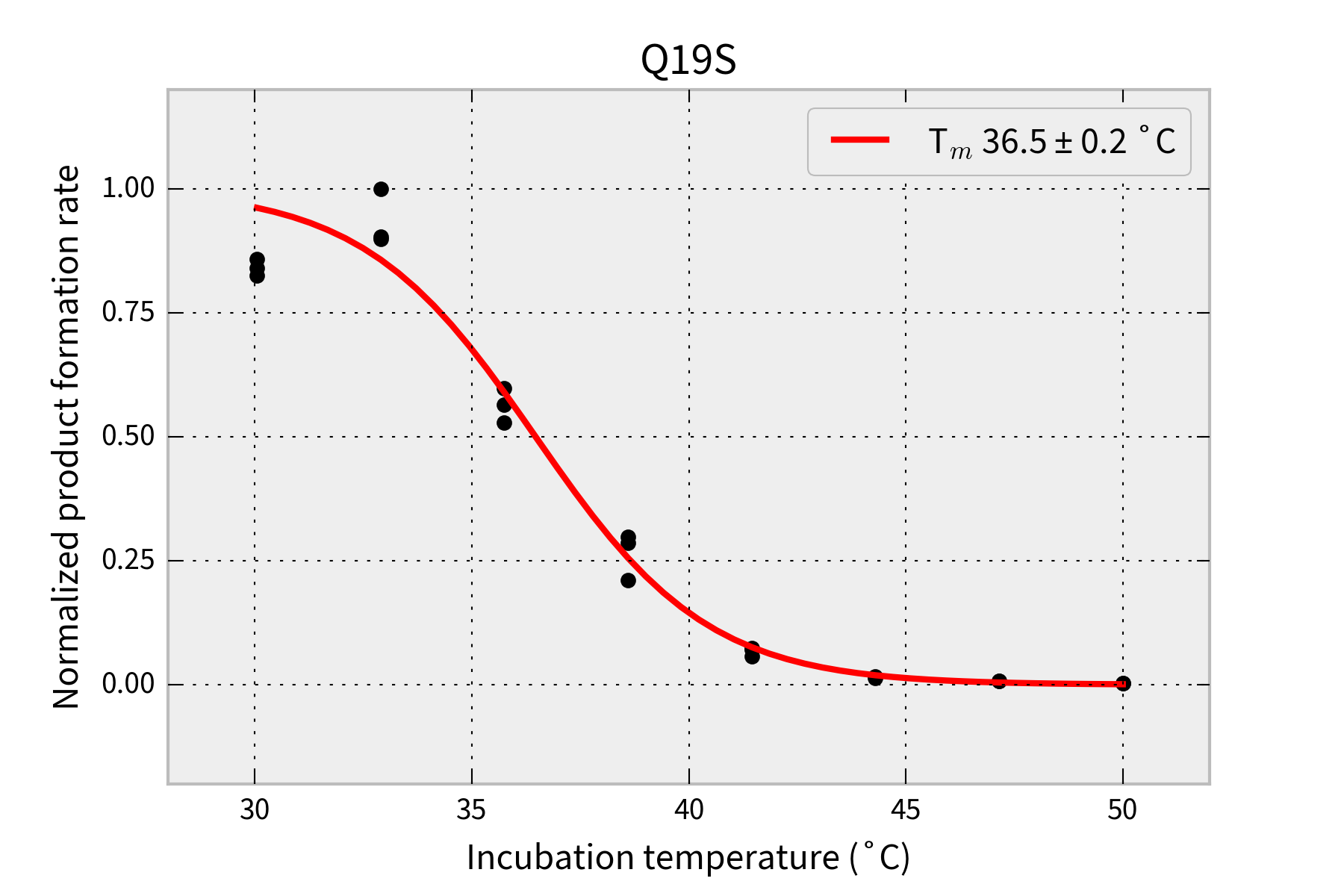

Supplement: S3 Figs — (ZIP) [file pone.0176255.s006.zip › S3 Figures/Q19S.png]

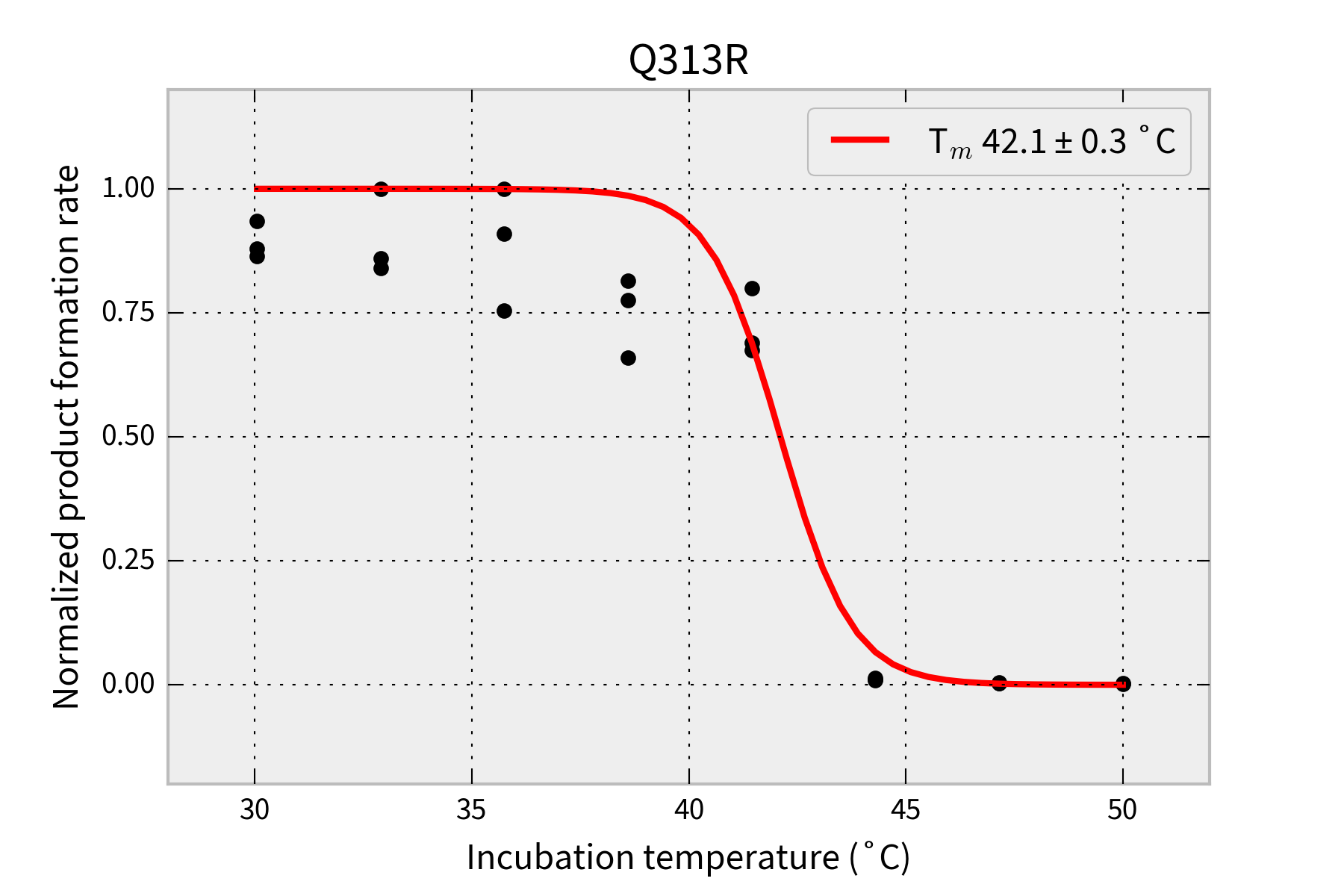

Supplement: S3 Figs — (ZIP) [file pone.0176255.s006.zip › S3 Figures/Q313R.png]

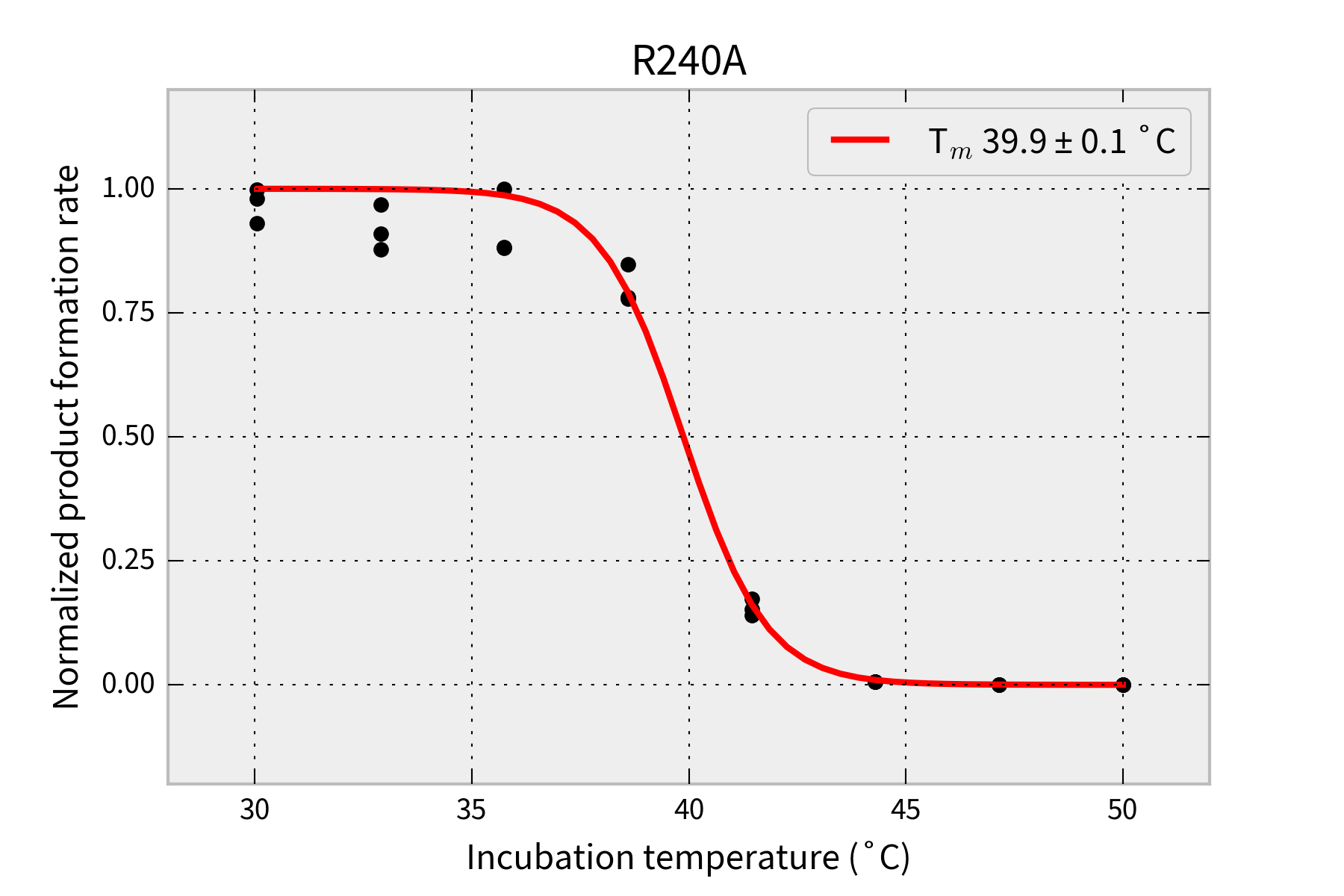

Supplement: S3 Figs — (ZIP) [file pone.0176255.s006.zip › S3 Figures/R240A.png]

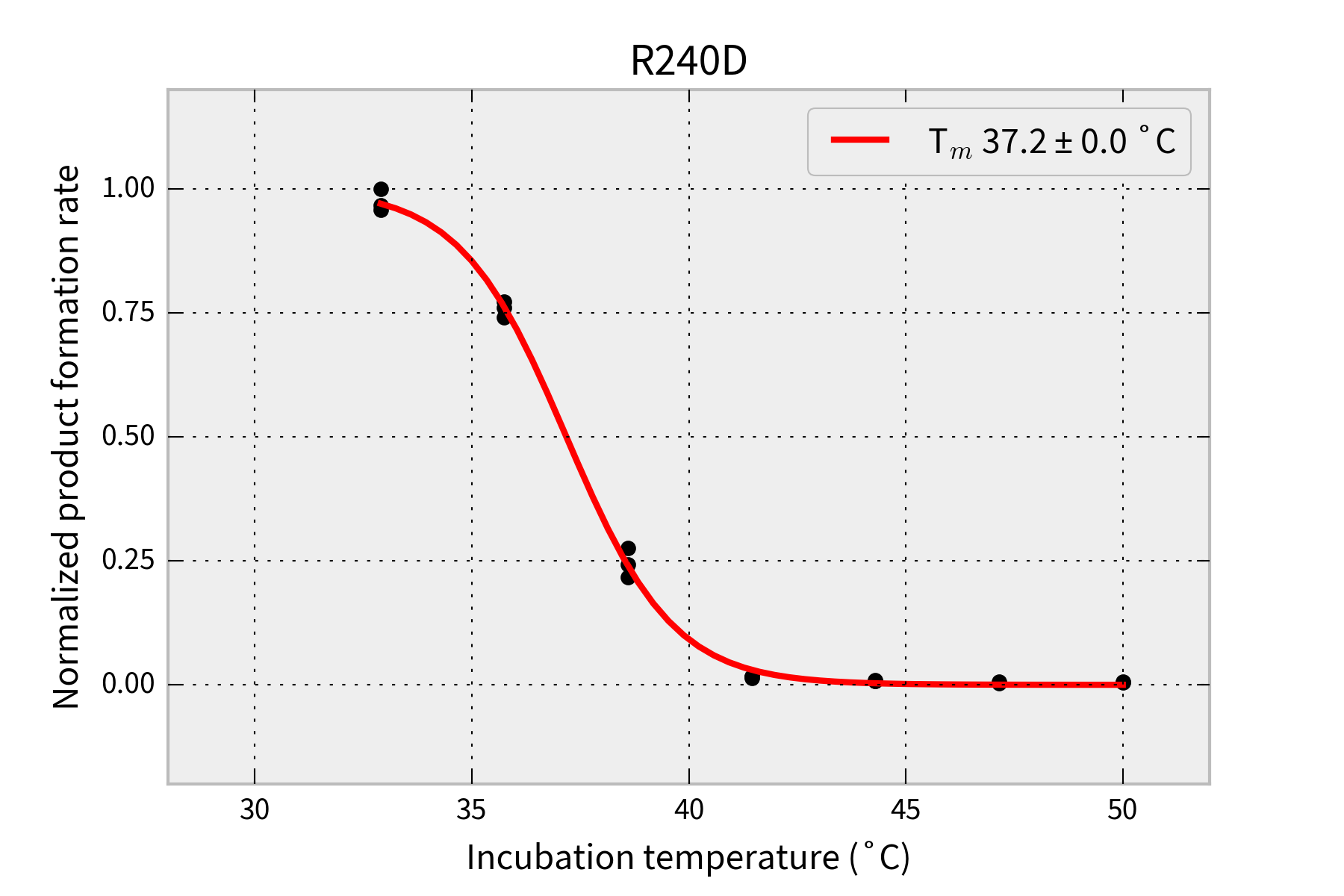

Supplement: S3 Figs — (ZIP) [file pone.0176255.s006.zip › S3 Figures/R240D.png]

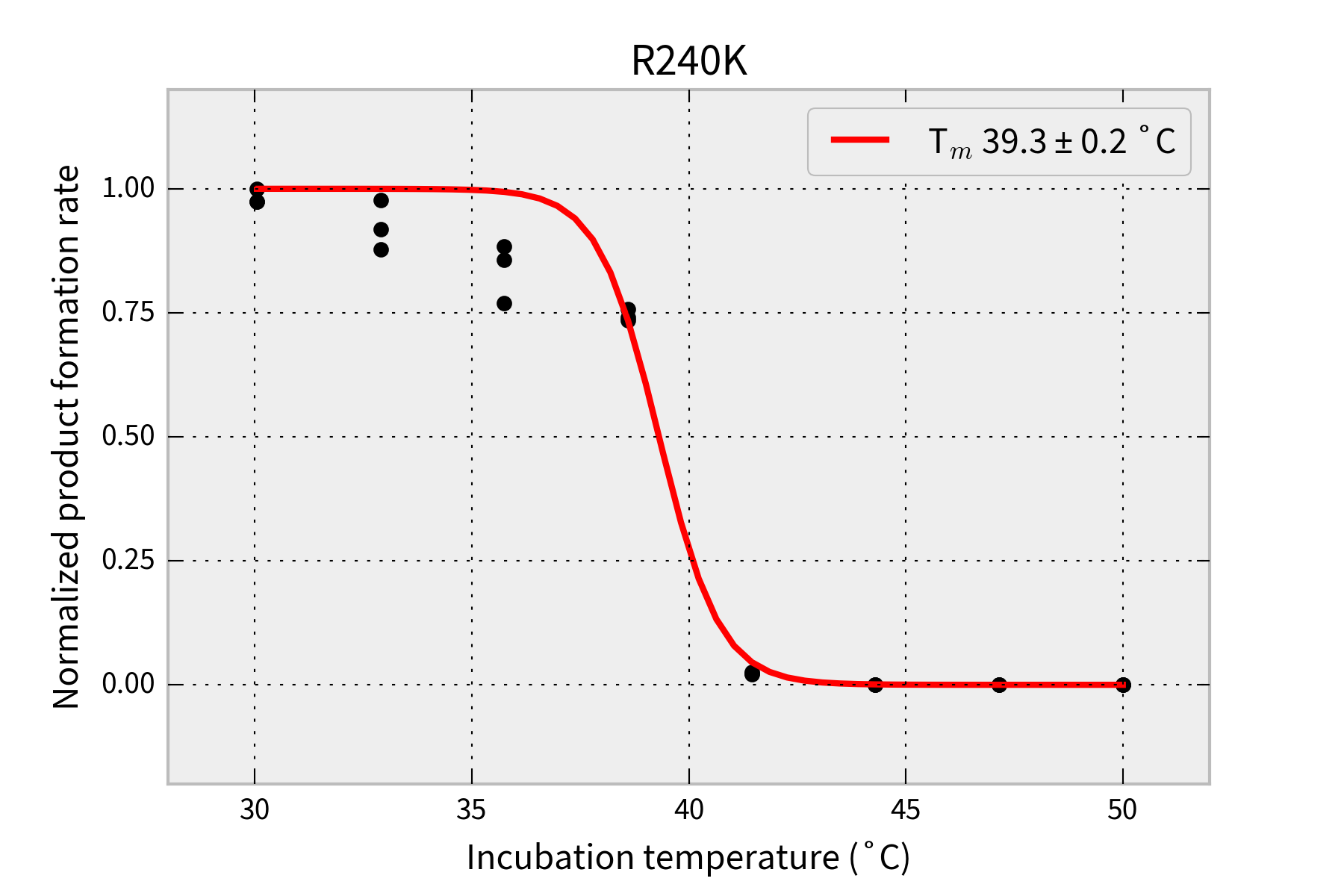

Supplement: S3 Figs — (ZIP) [file pone.0176255.s006.zip › S3 Figures/R240K.png]

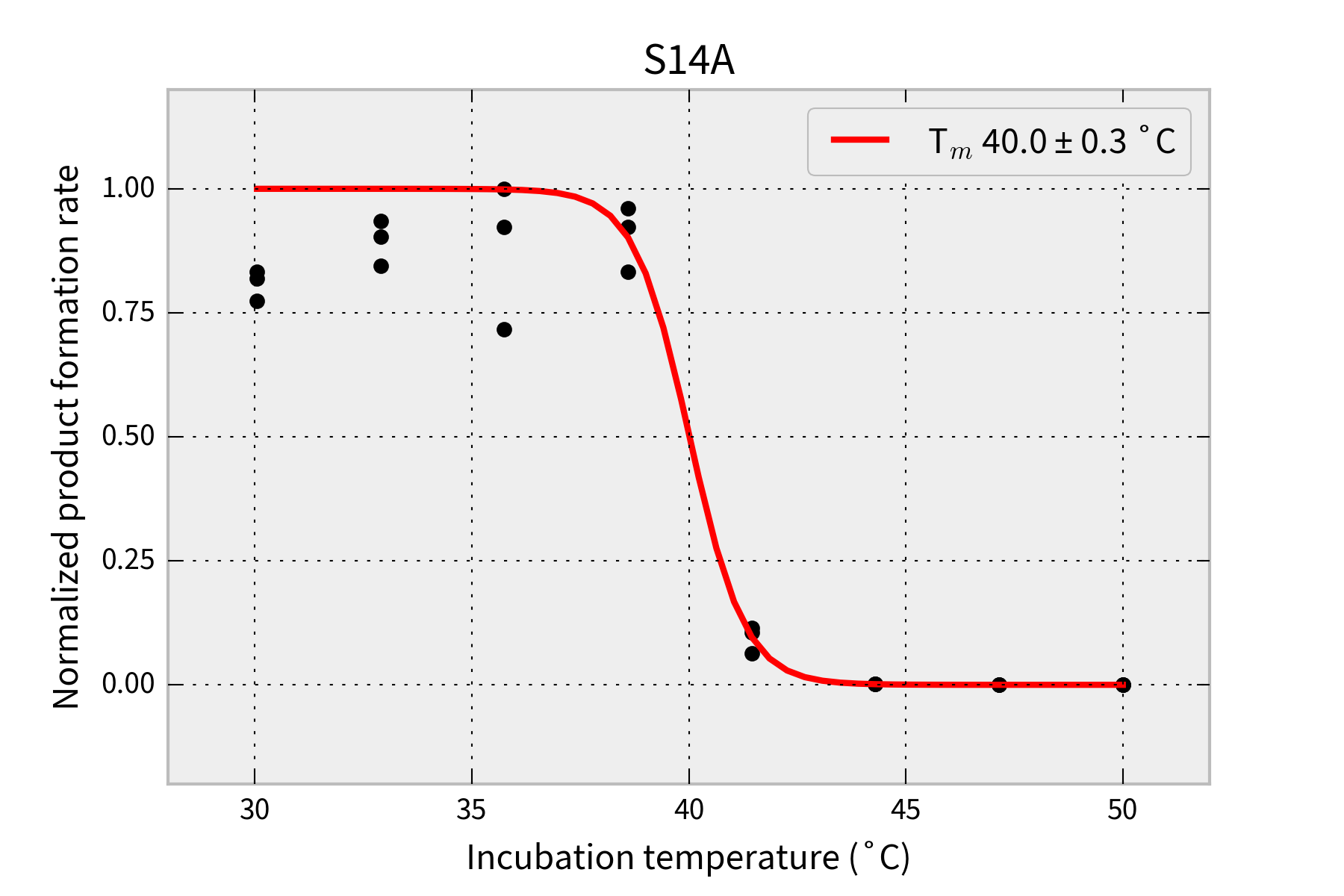

Supplement: S3 Figs — (ZIP) [file pone.0176255.s006.zip › S3 Figures/S14A.png]

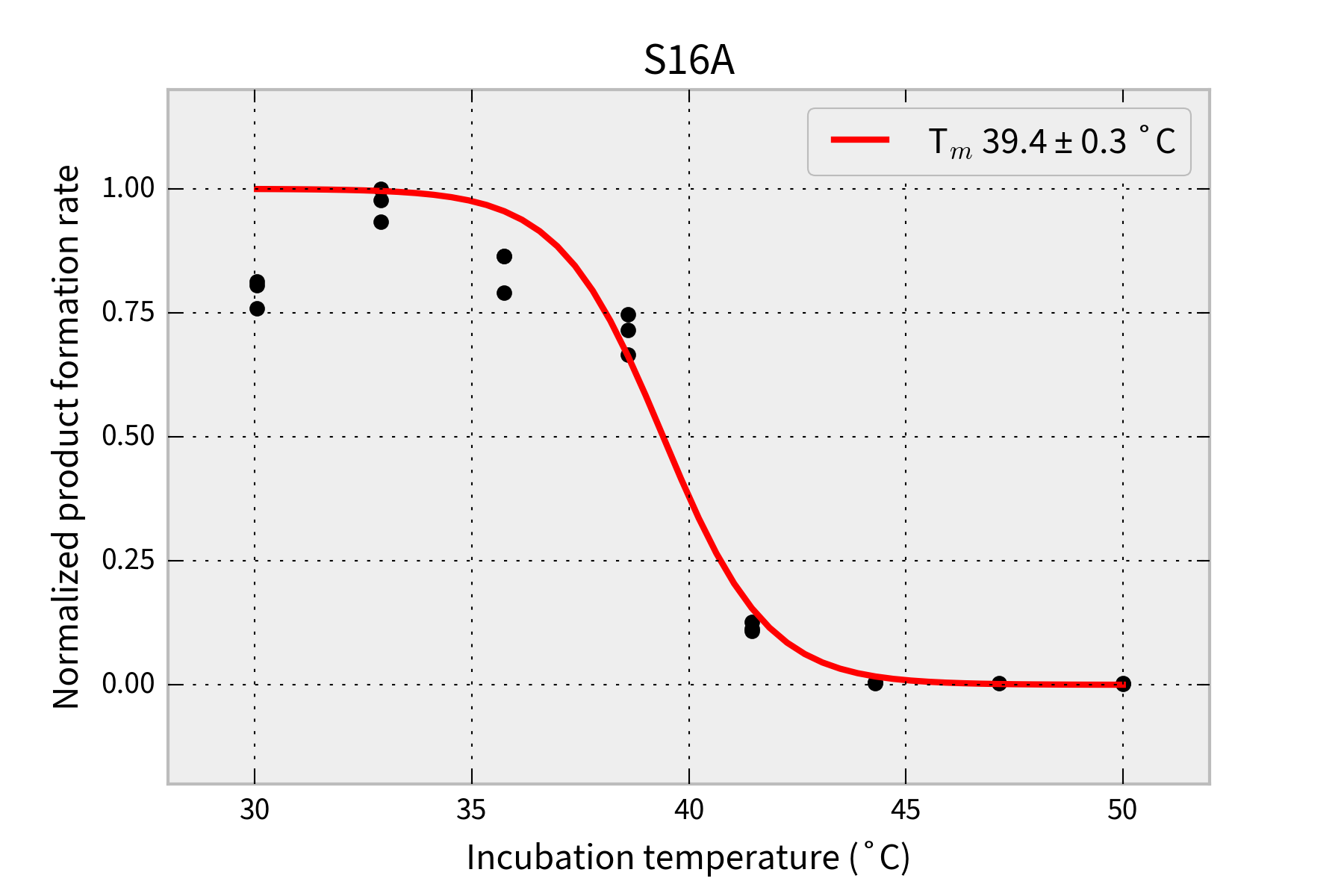

Supplement: S3 Figs — (ZIP) [file pone.0176255.s006.zip › S3 Figures/S16A.png]

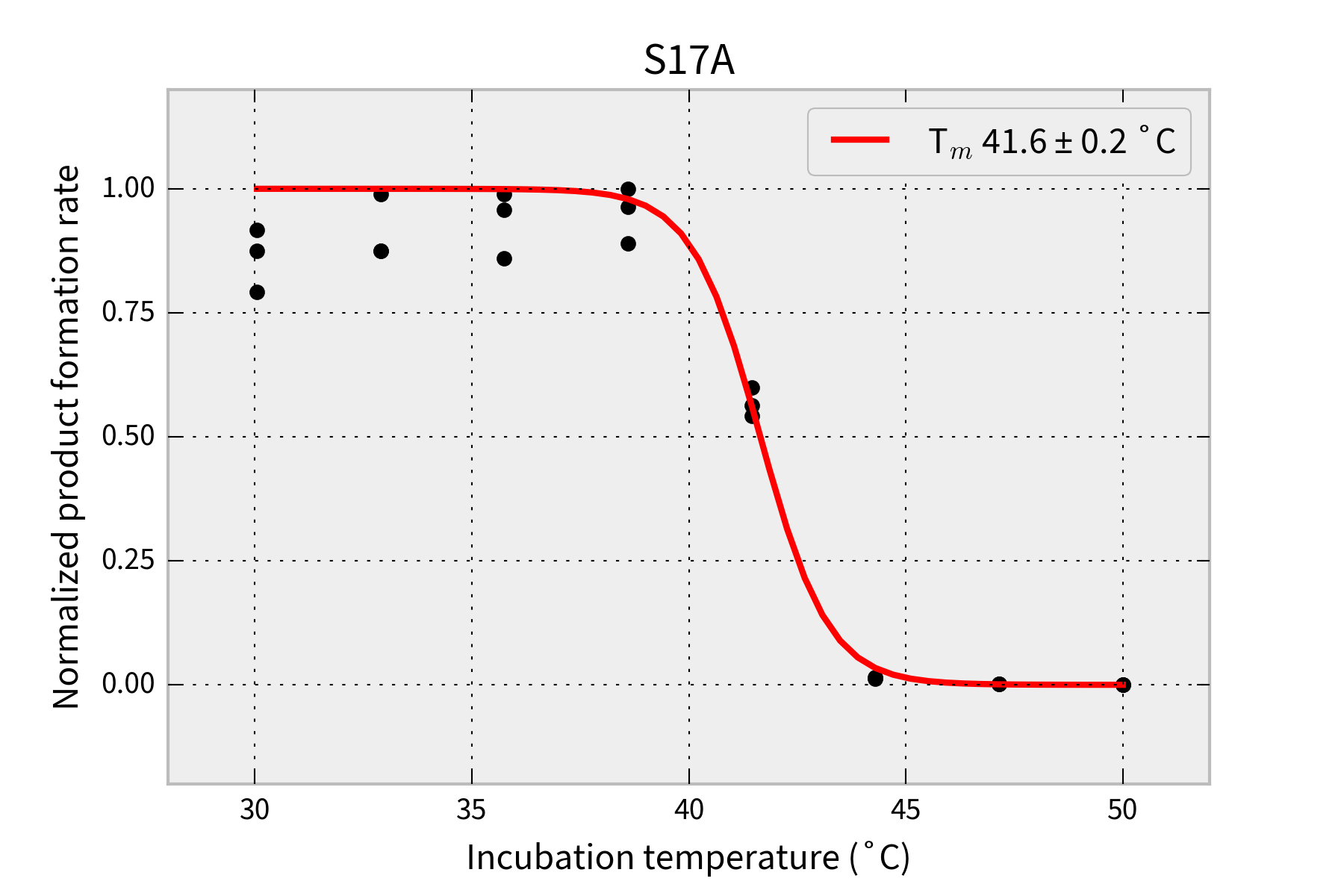

Supplement: S3 Figs — (ZIP) [file pone.0176255.s006.zip › S3 Figures/S17A.png]

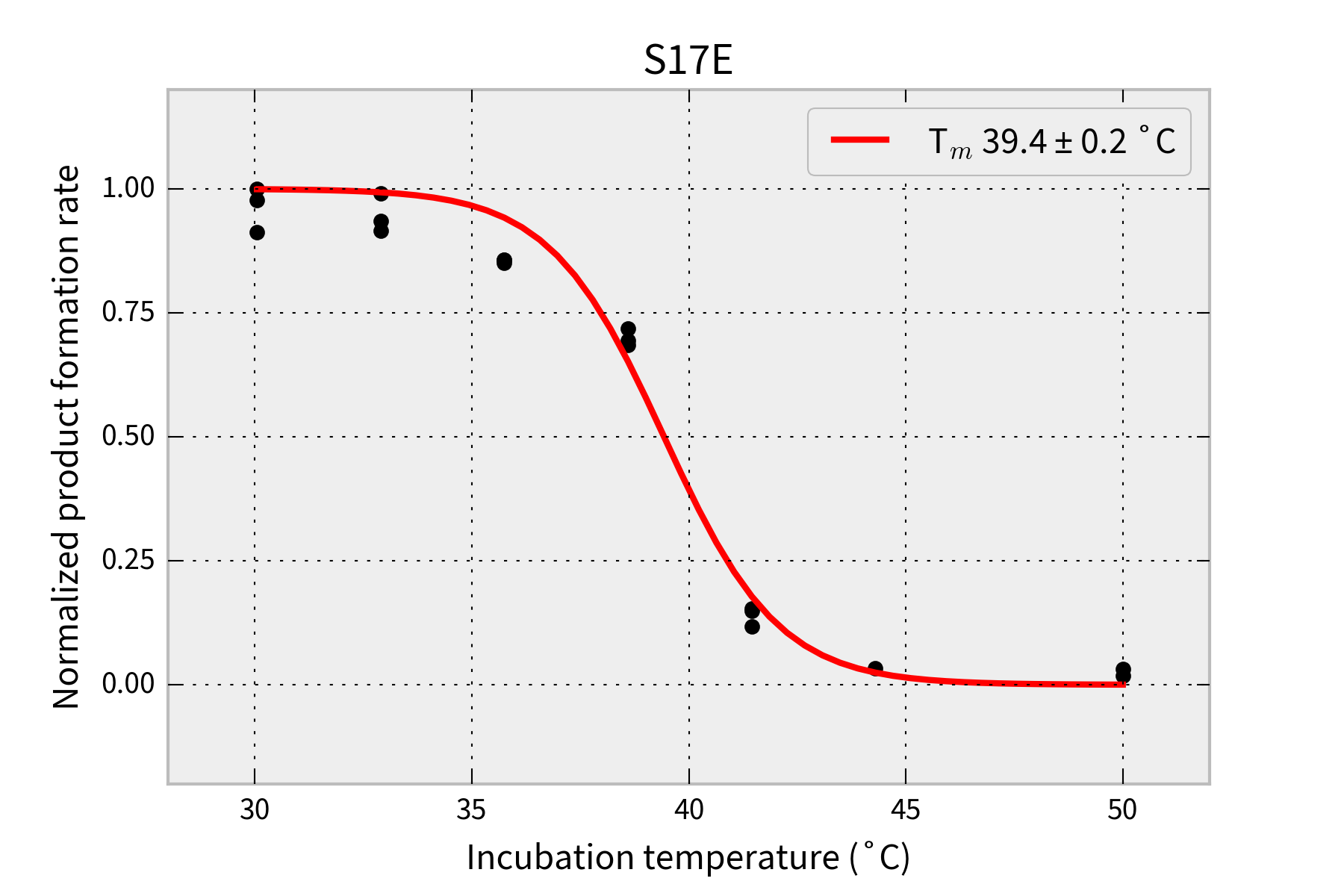

Supplement: S3 Figs — (ZIP) [file pone.0176255.s006.zip › S3 Figures/S17E.png]

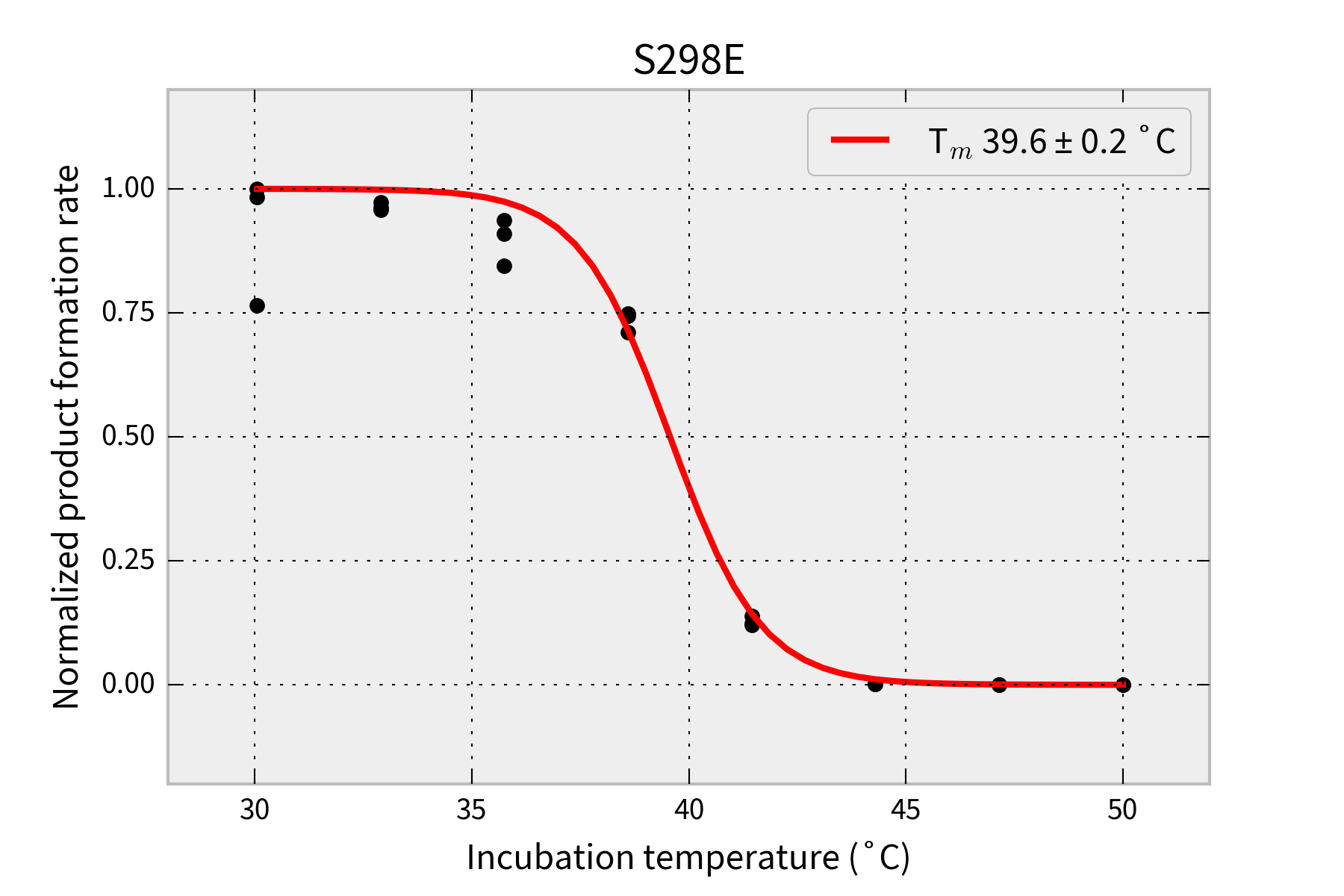

Supplement: S3 Figs — (ZIP) [file pone.0176255.s006.zip › S3 Figures/S298E.png]

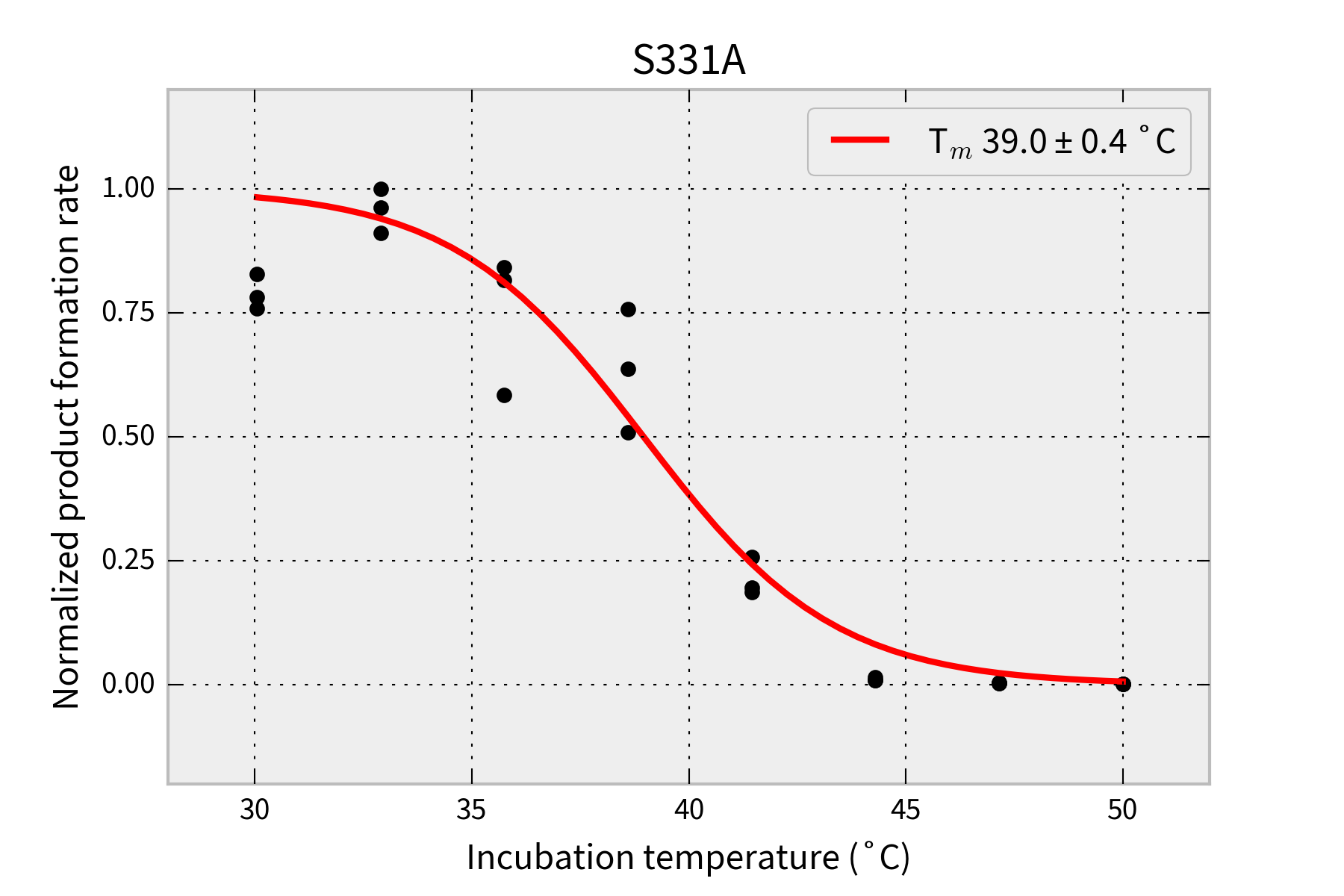

Supplement: S3 Figs — (ZIP) [file pone.0176255.s006.zip › S3 Figures/S331A.png]

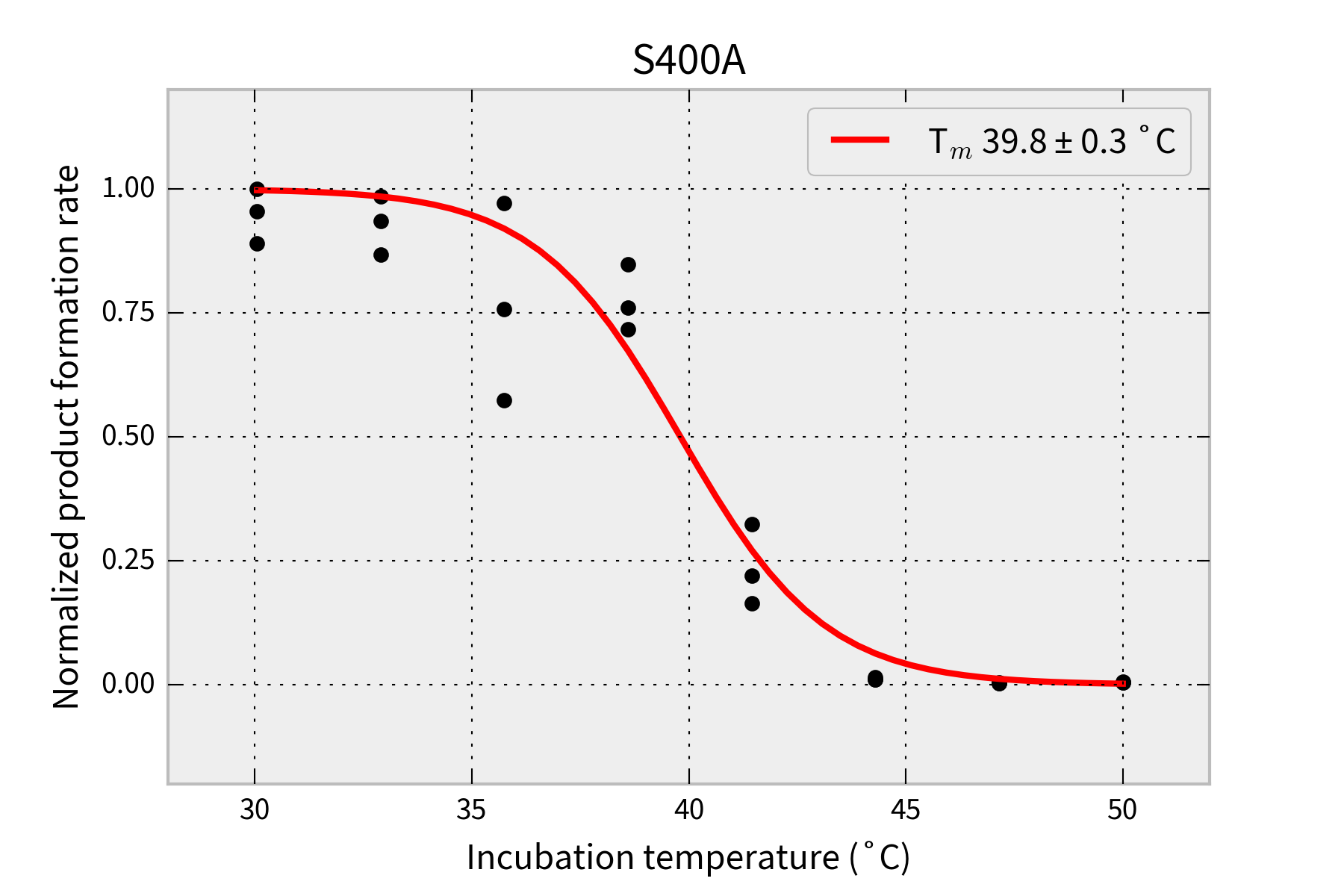

Supplement: S3 Figs — (ZIP) [file pone.0176255.s006.zip › S3 Figures/S400A.png]

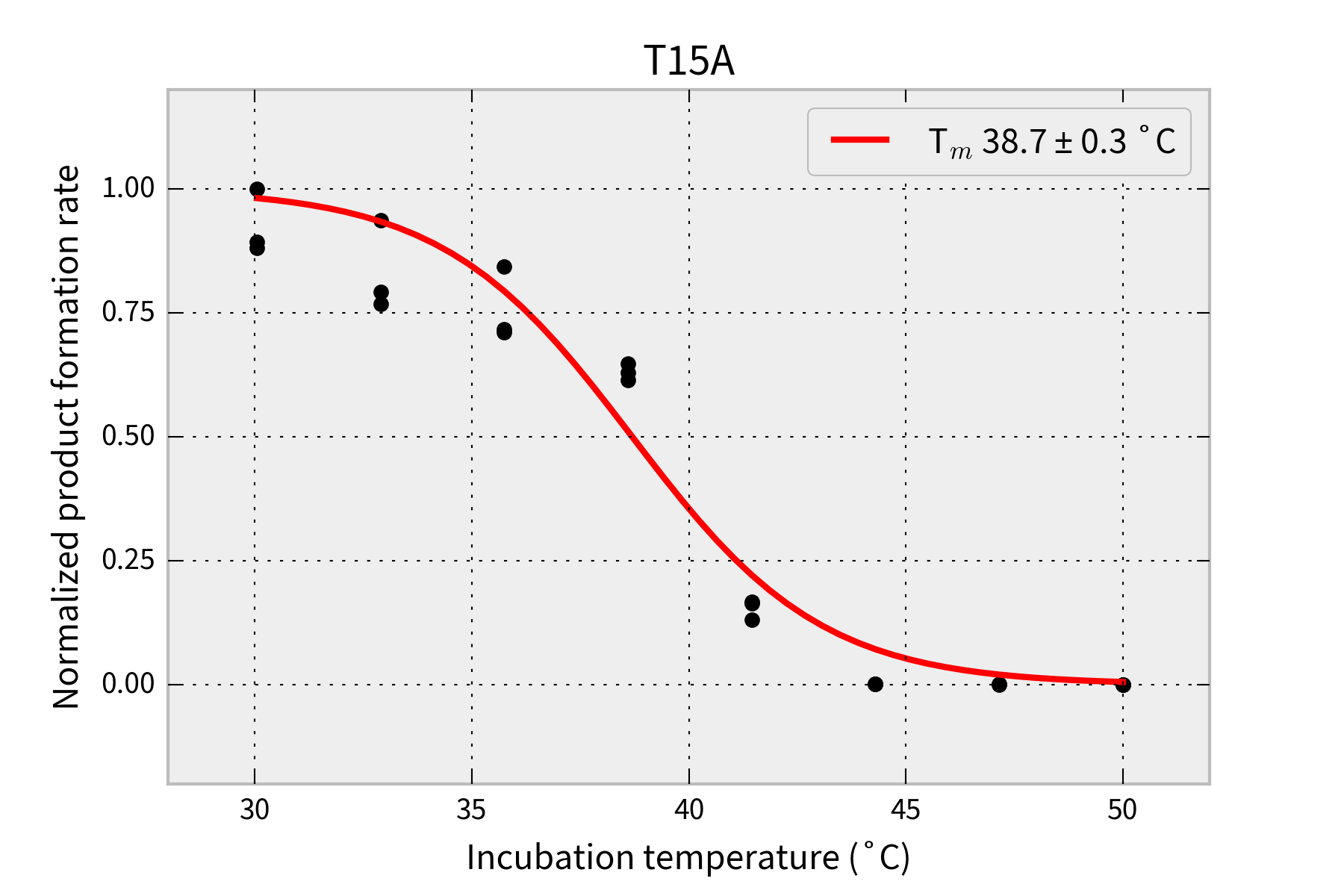

Supplement: S3 Figs — (ZIP) [file pone.0176255.s006.zip › S3 Figures/T15A.png]

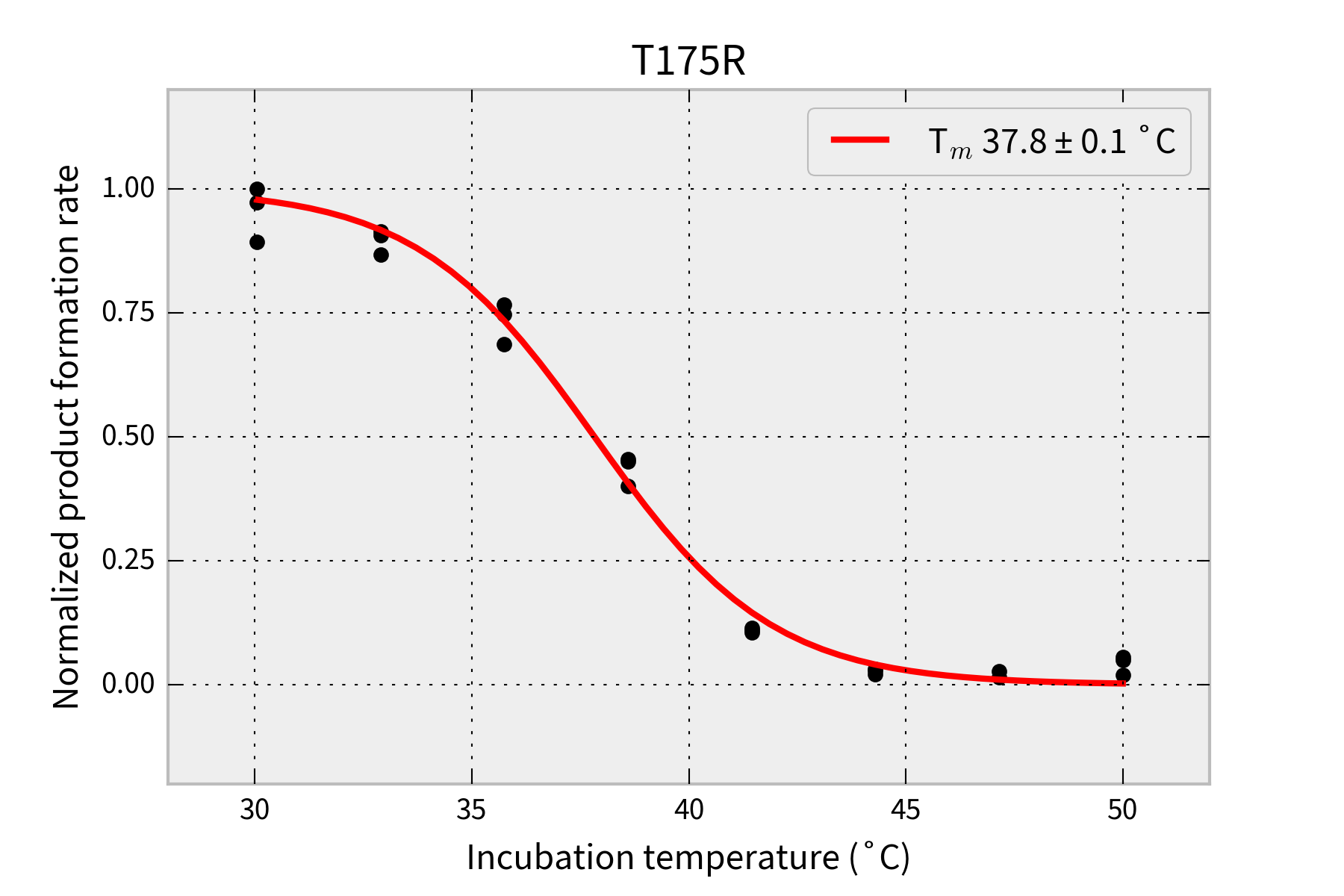

Supplement: S3 Figs — (ZIP) [file pone.0176255.s006.zip › S3 Figures/T175R.png]

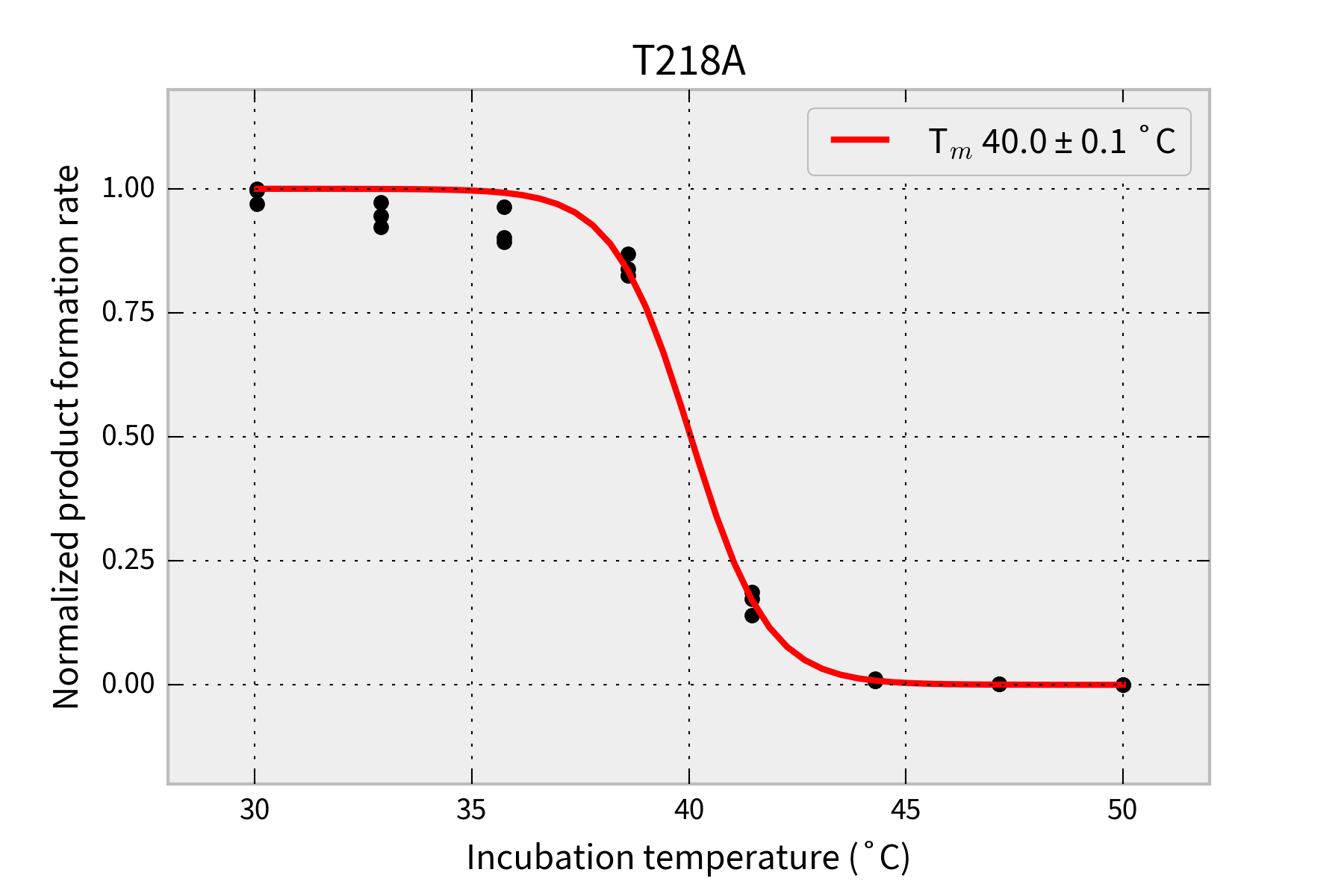

Supplement: S3 Figs — (ZIP) [file pone.0176255.s006.zip › S3 Figures/T218A.png]

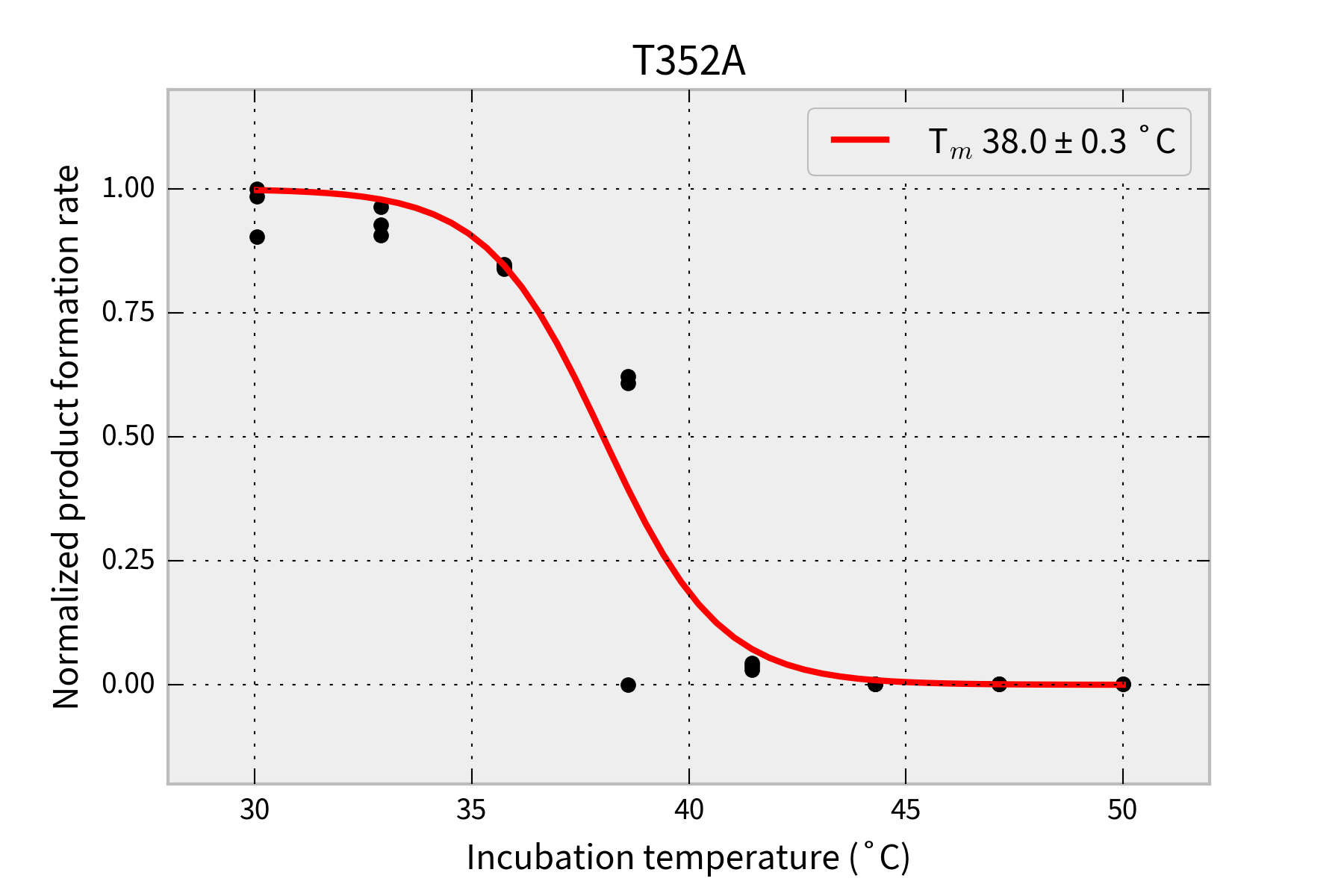

Supplement: S3 Figs — (ZIP) [file pone.0176255.s006.zip › S3 Figures/T352A.png]

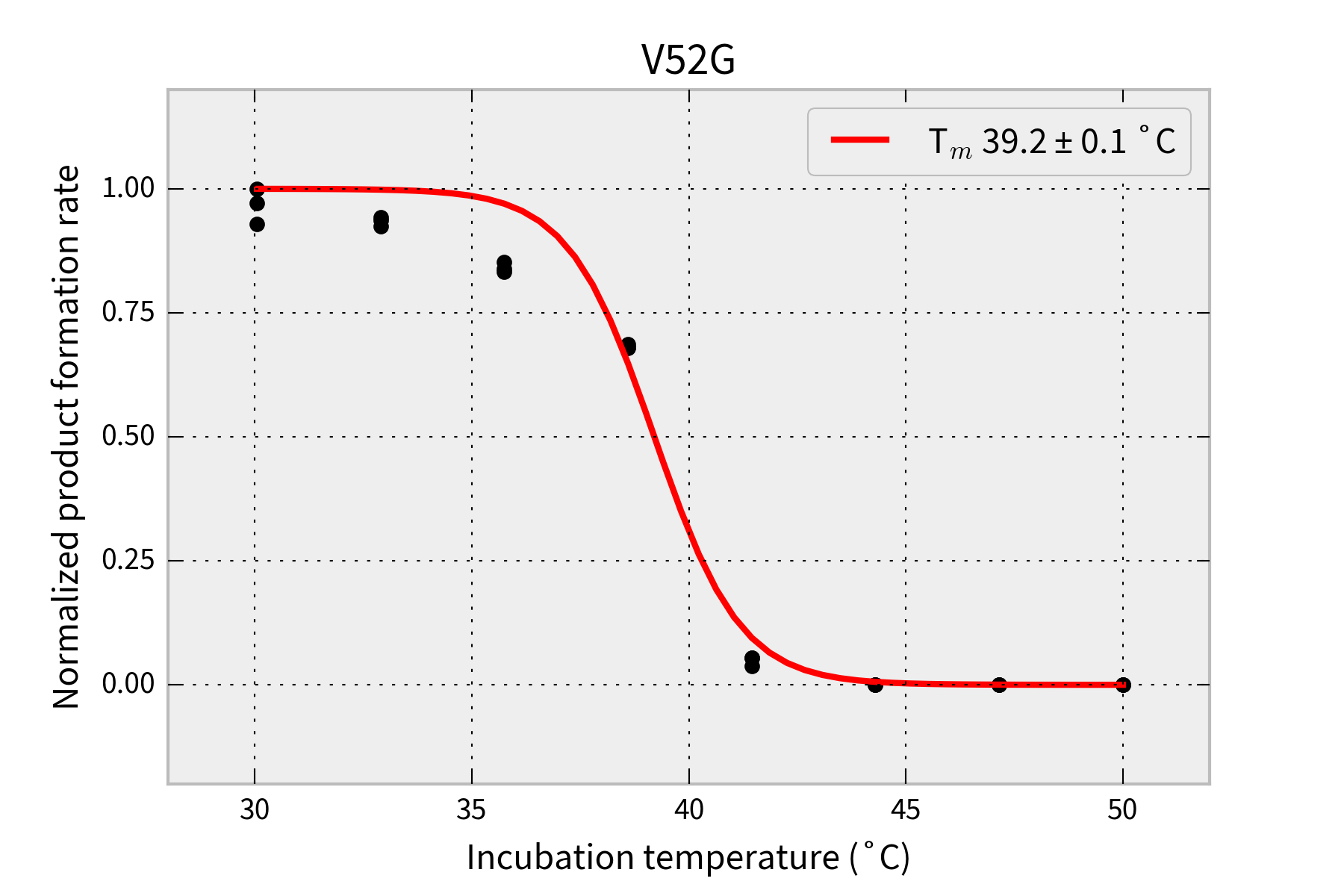

Supplement: S3 Figs — (ZIP) [file pone.0176255.s006.zip › S3 Figures/V52G.png]

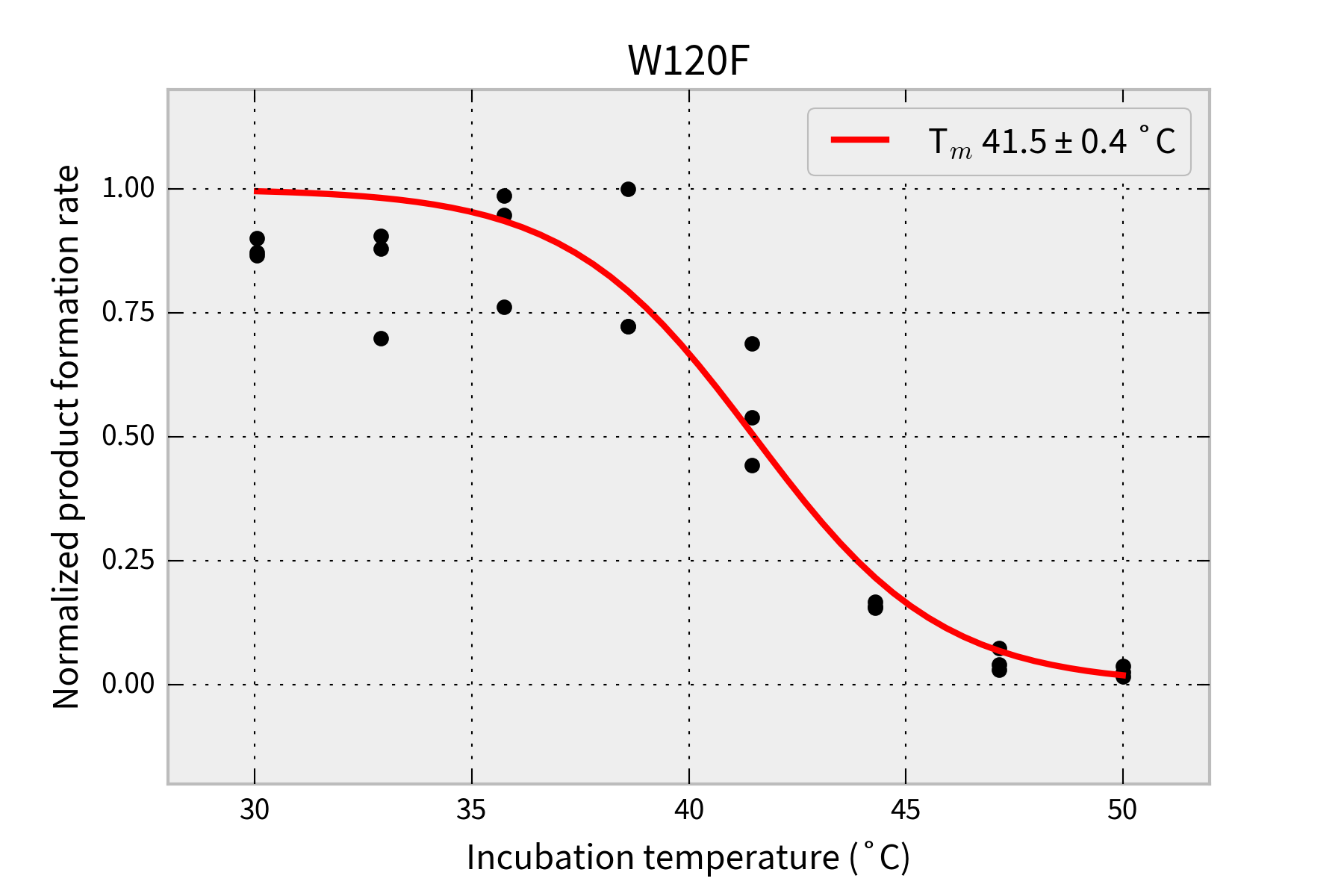

Supplement: S3 Figs — (ZIP) [file pone.0176255.s006.zip › S3 Figures/W120F.png]

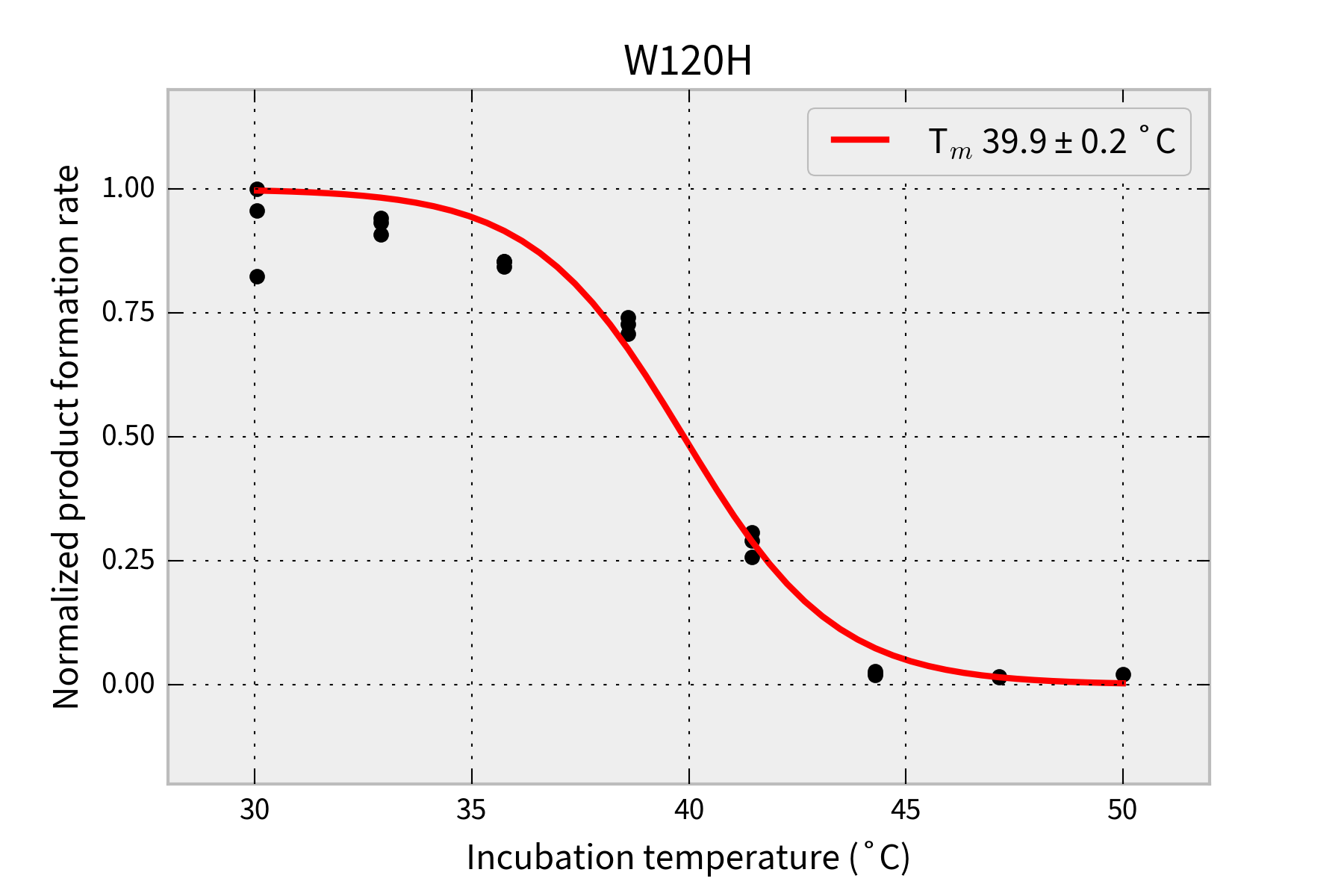

Supplement: S3 Figs — (ZIP) [file pone.0176255.s006.zip › S3 Figures/W120H.png]

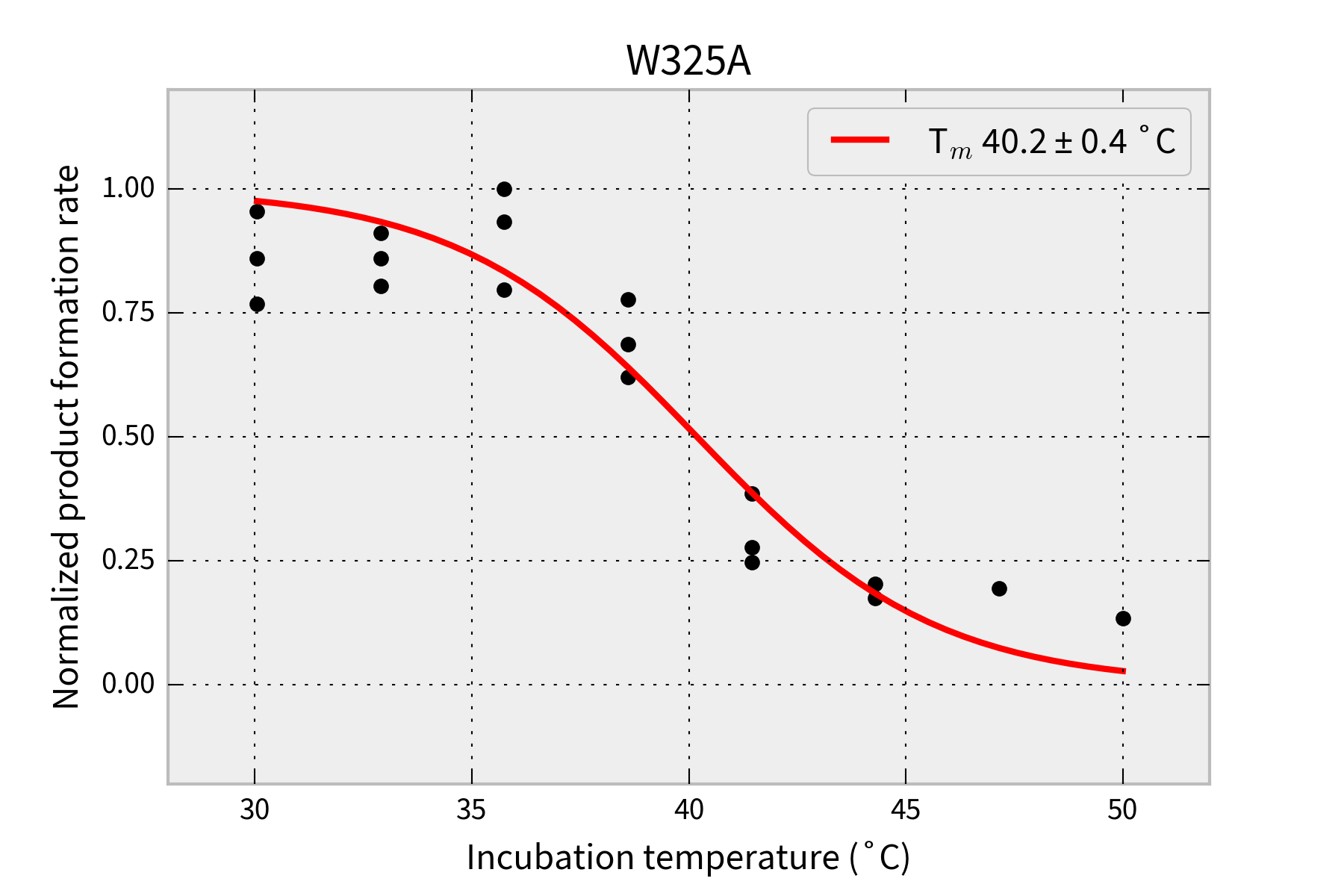

Supplement: S3 Figs — (ZIP) [file pone.0176255.s006.zip › S3 Figures/W325A.png]
