# Supplementary figures and images for: Thermal stability and kinetic constants for 129 variants of a family 1 glycoside hydrolase reveal that enzyme activity and stability can be separately designed (part 2 of 2)
Source: PLoS One. 2017 May 22;12(5):e0176255. doi: 10.1371/journal.pone.0176255 (PMC5439667; doi:10.1371/journal.pone.0176255)

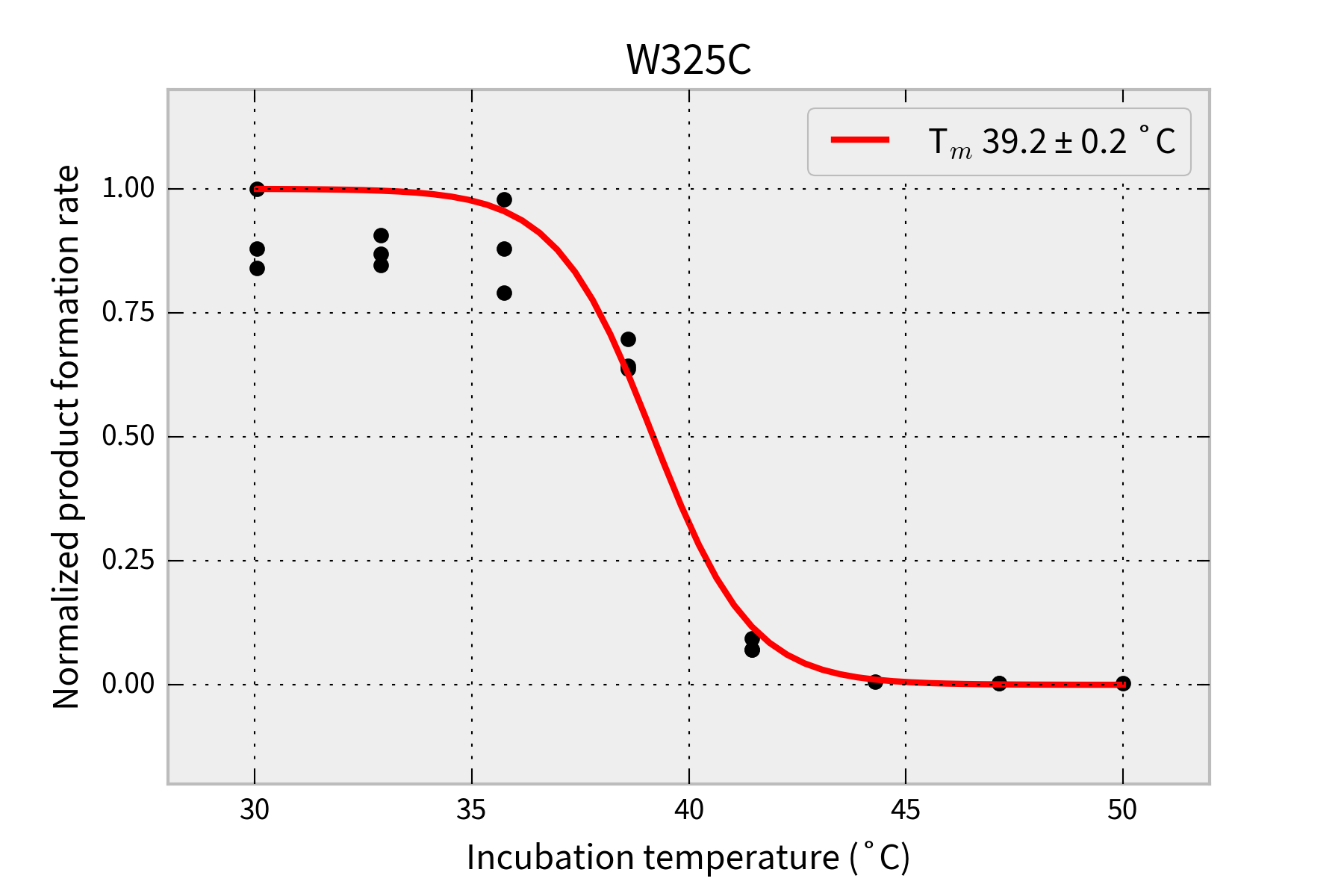

Supplement: S3 Figs — (ZIP) [file pone.0176255.s006.zip › S3 Figures/W325C.png]

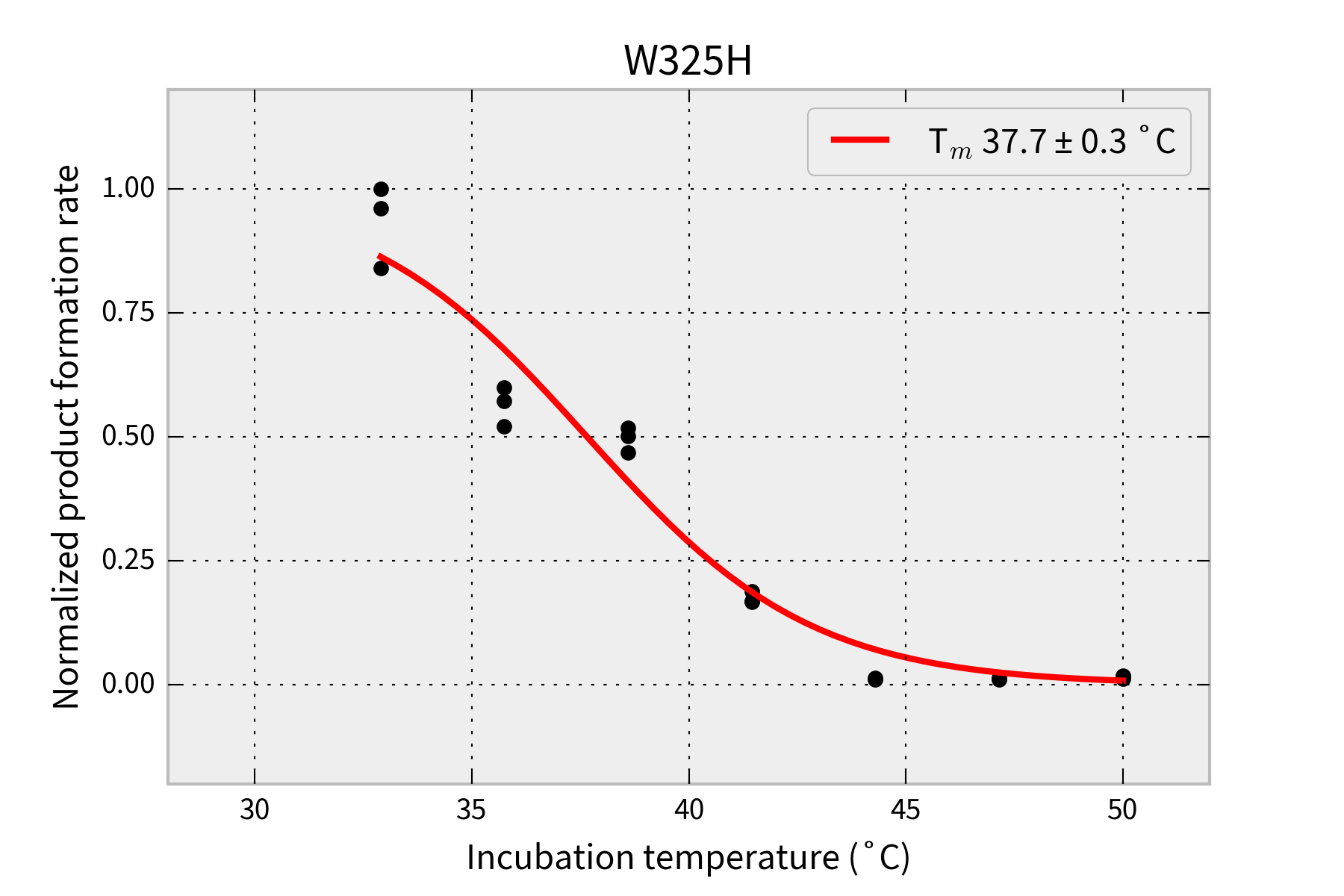

Supplement: S3 Figs — (ZIP) [file pone.0176255.s006.zip › S3 Figures/W325H.png]

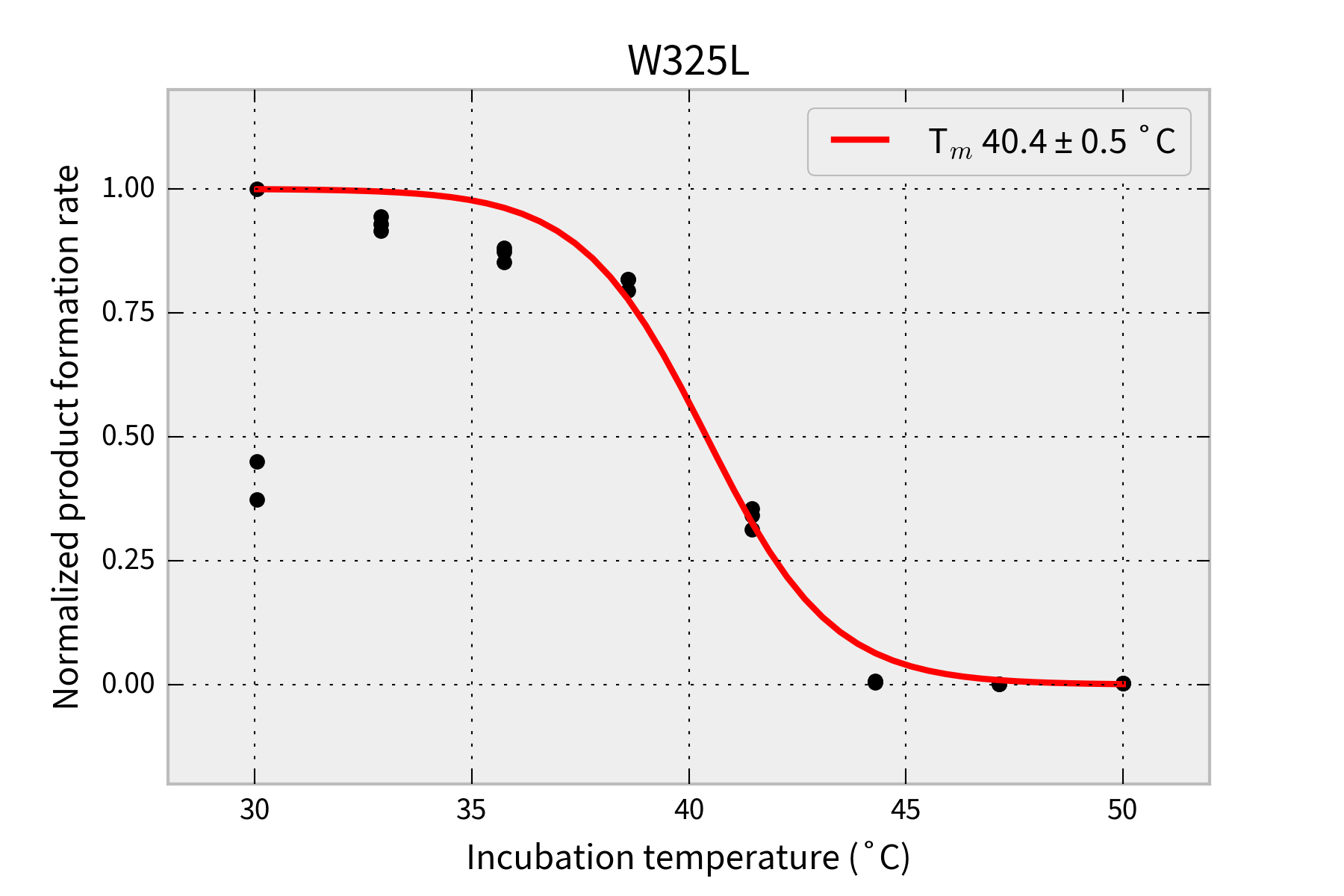

Supplement: S3 Figs — (ZIP) [file pone.0176255.s006.zip › S3 Figures/W325L.png]

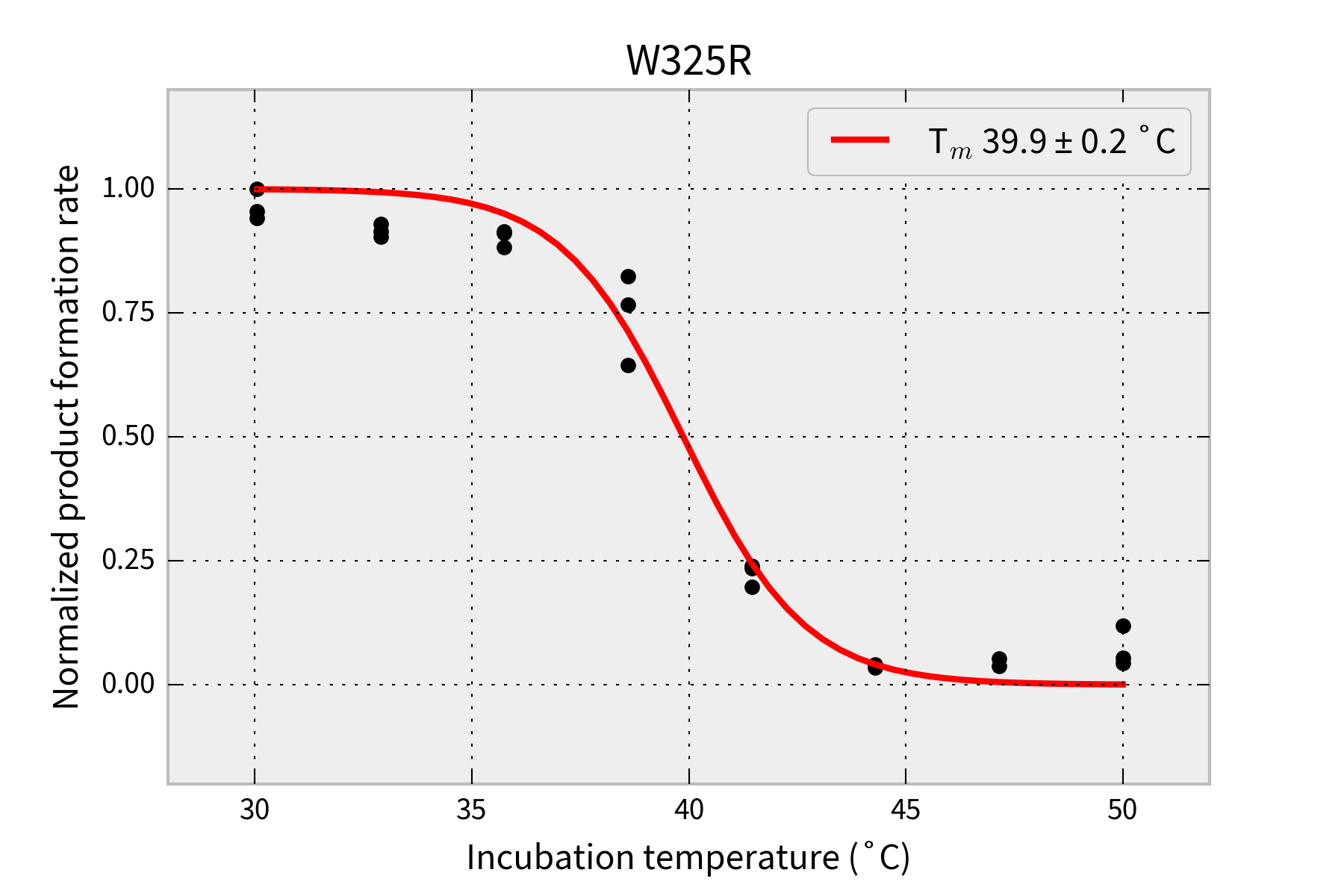

Supplement: S3 Figs — (ZIP) [file pone.0176255.s006.zip › S3 Figures/W325R.png]

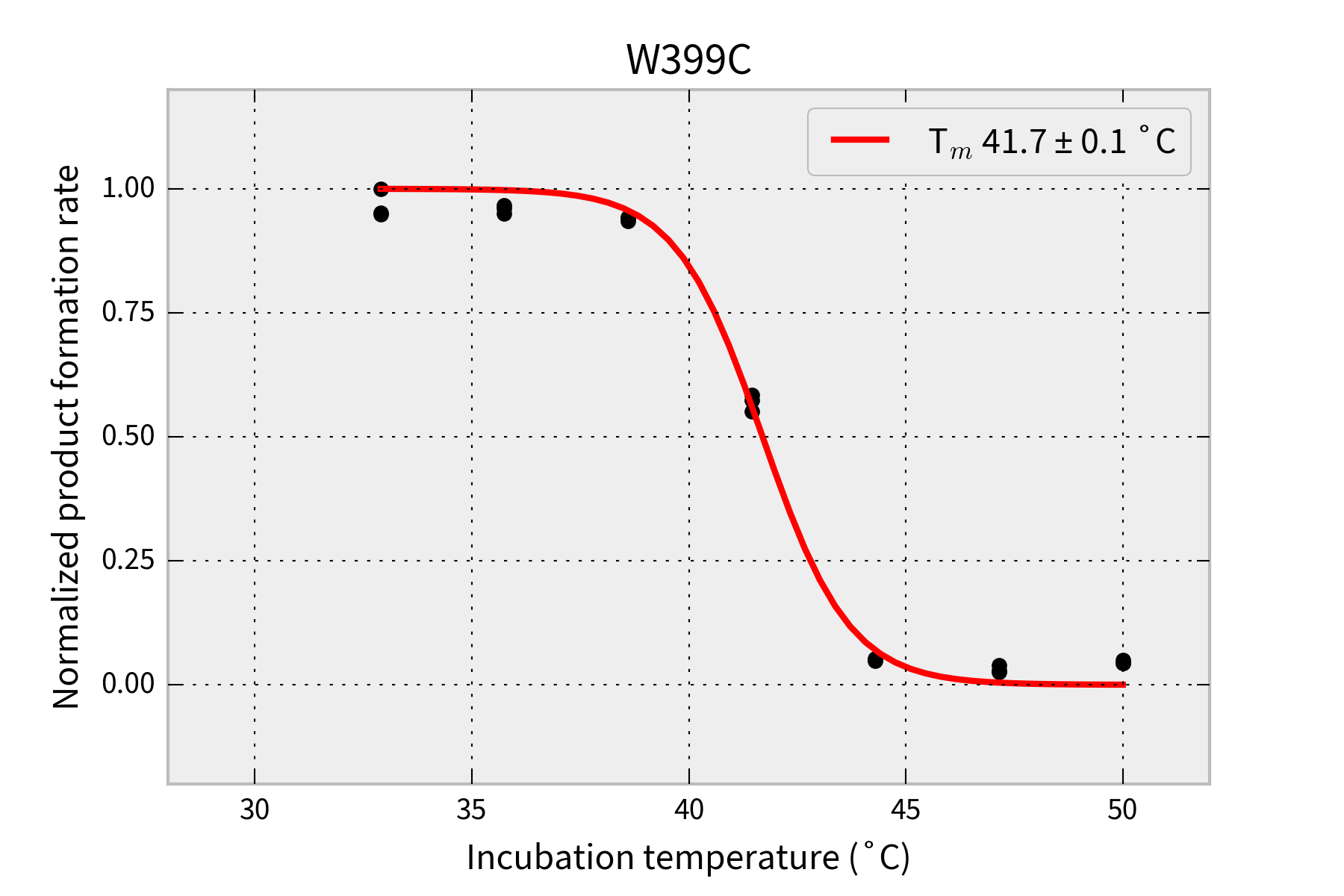

Supplement: S3 Figs — (ZIP) [file pone.0176255.s006.zip › S3 Figures/W399C.png]

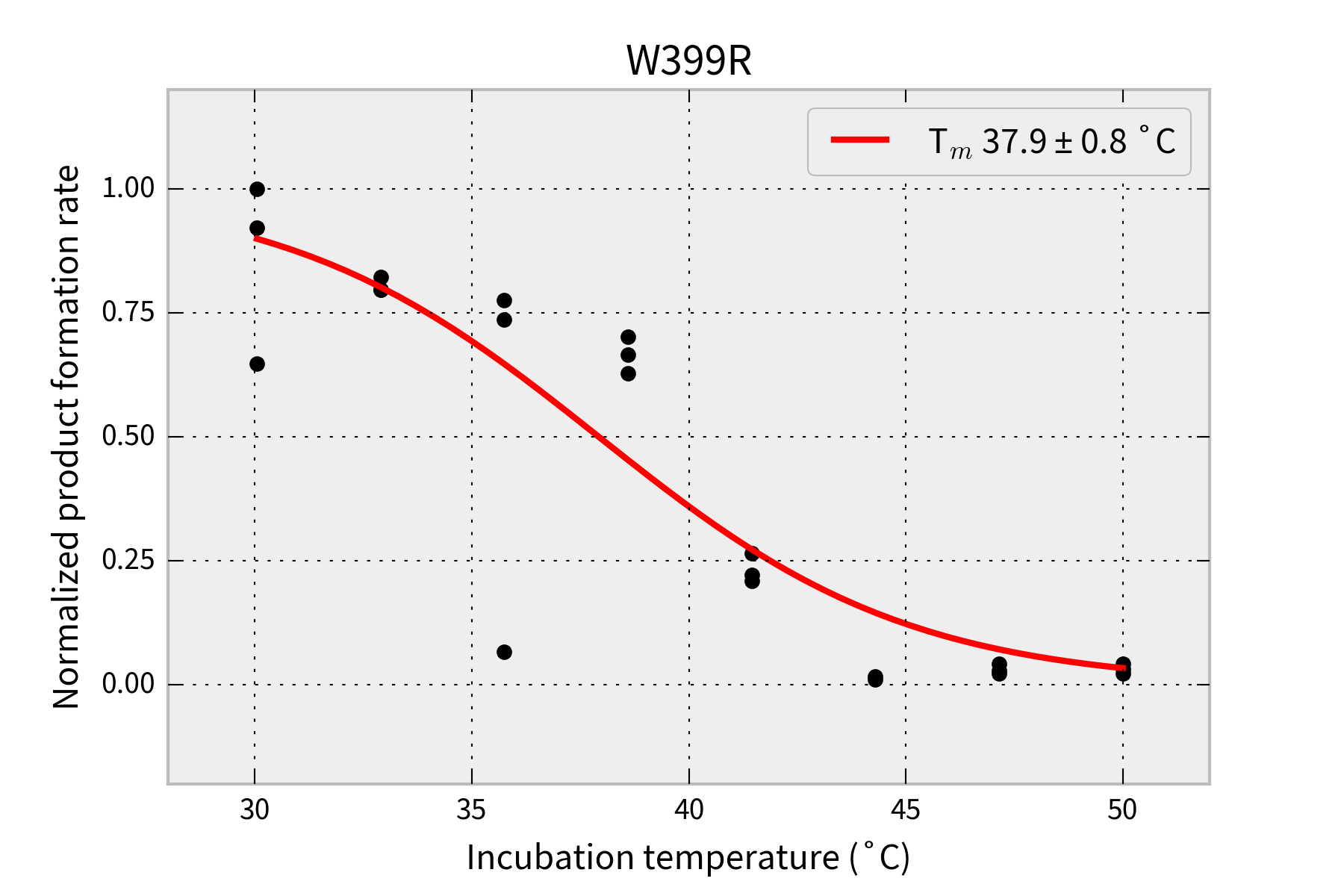

Supplement: S3 Figs — (ZIP) [file pone.0176255.s006.zip › S3 Figures/W399R.png]

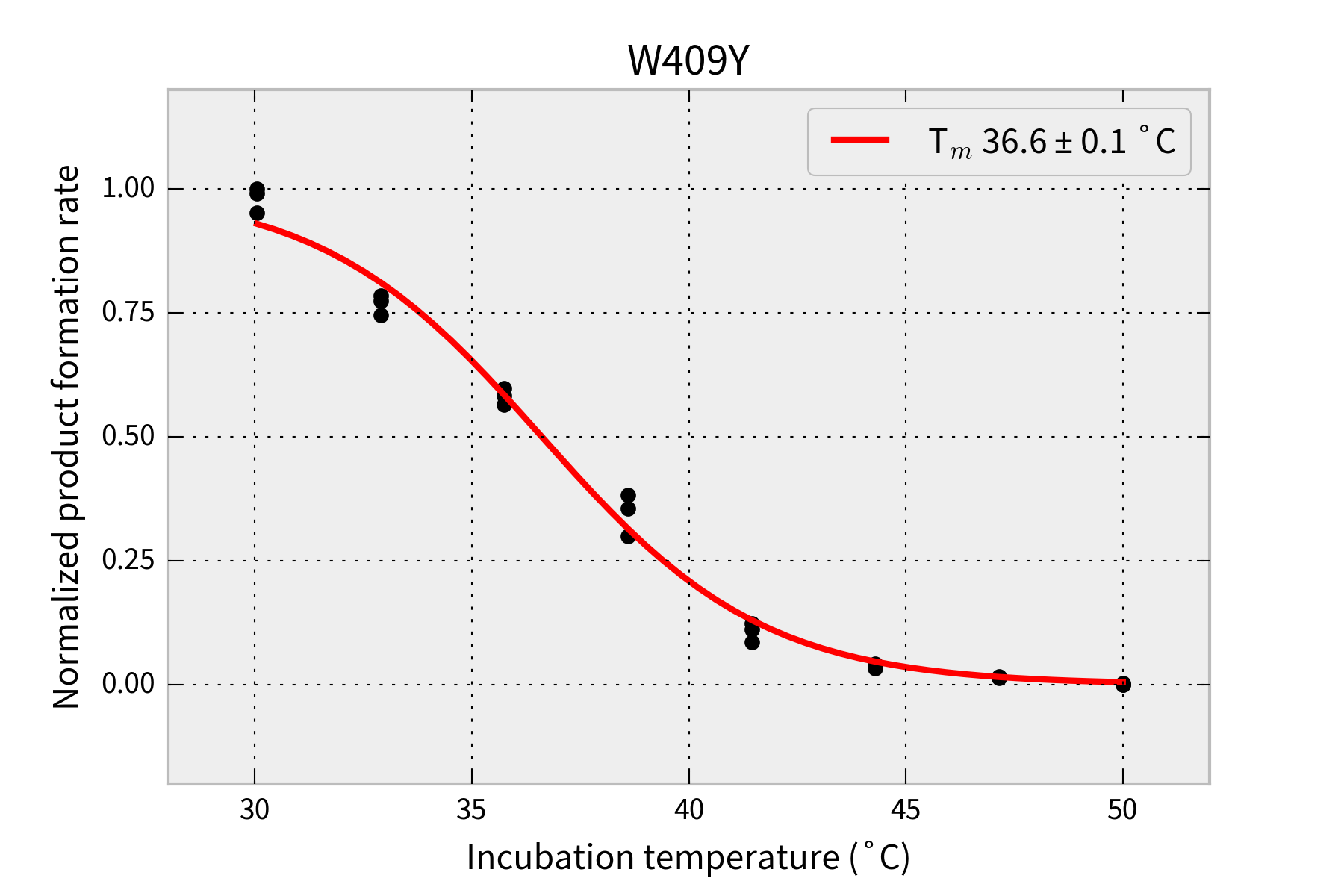

Supplement: S3 Figs — (ZIP) [file pone.0176255.s006.zip › S3 Figures/W409Y.png]

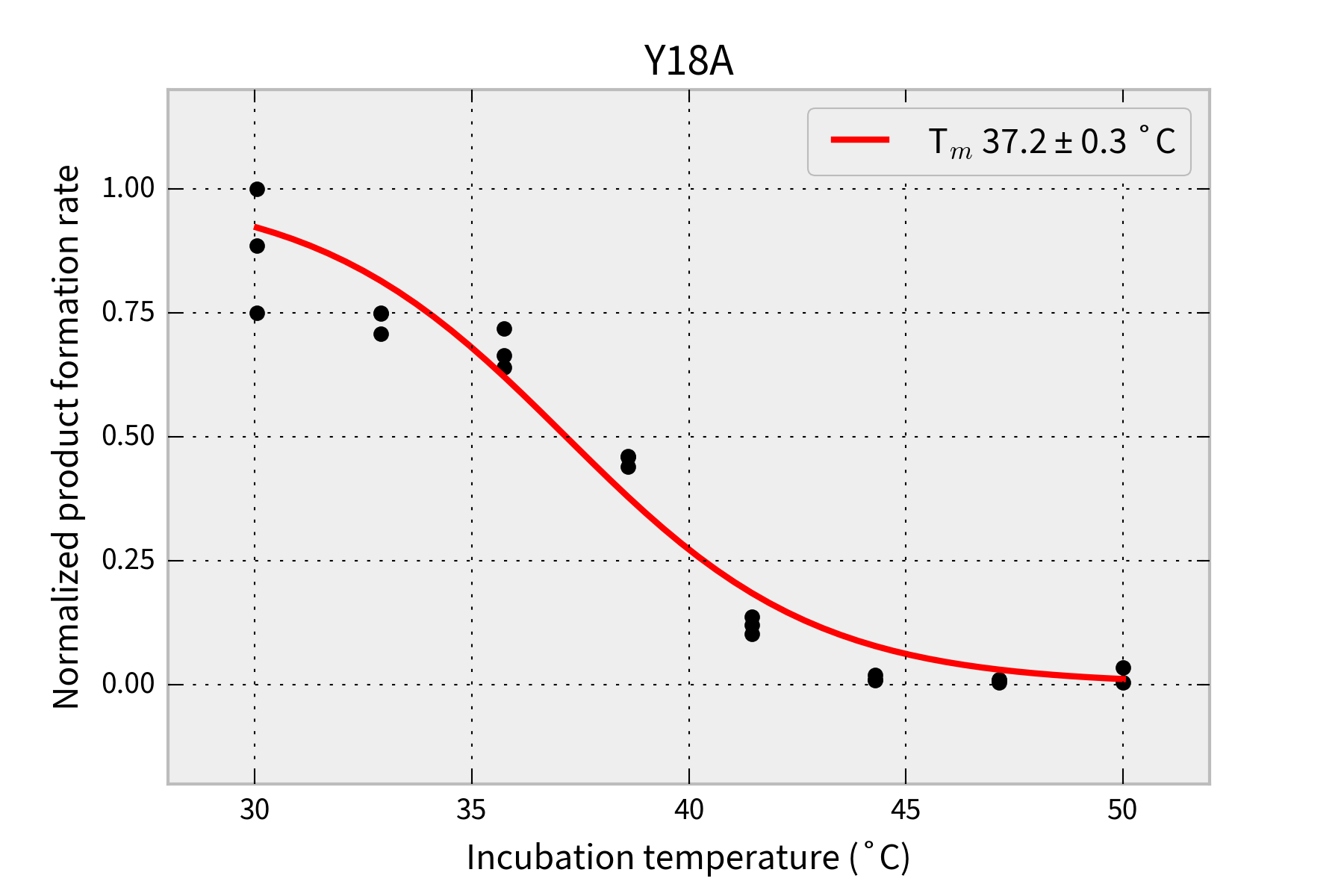

Supplement: S3 Figs — (ZIP) [file pone.0176255.s006.zip › S3 Figures/Y18A.png]

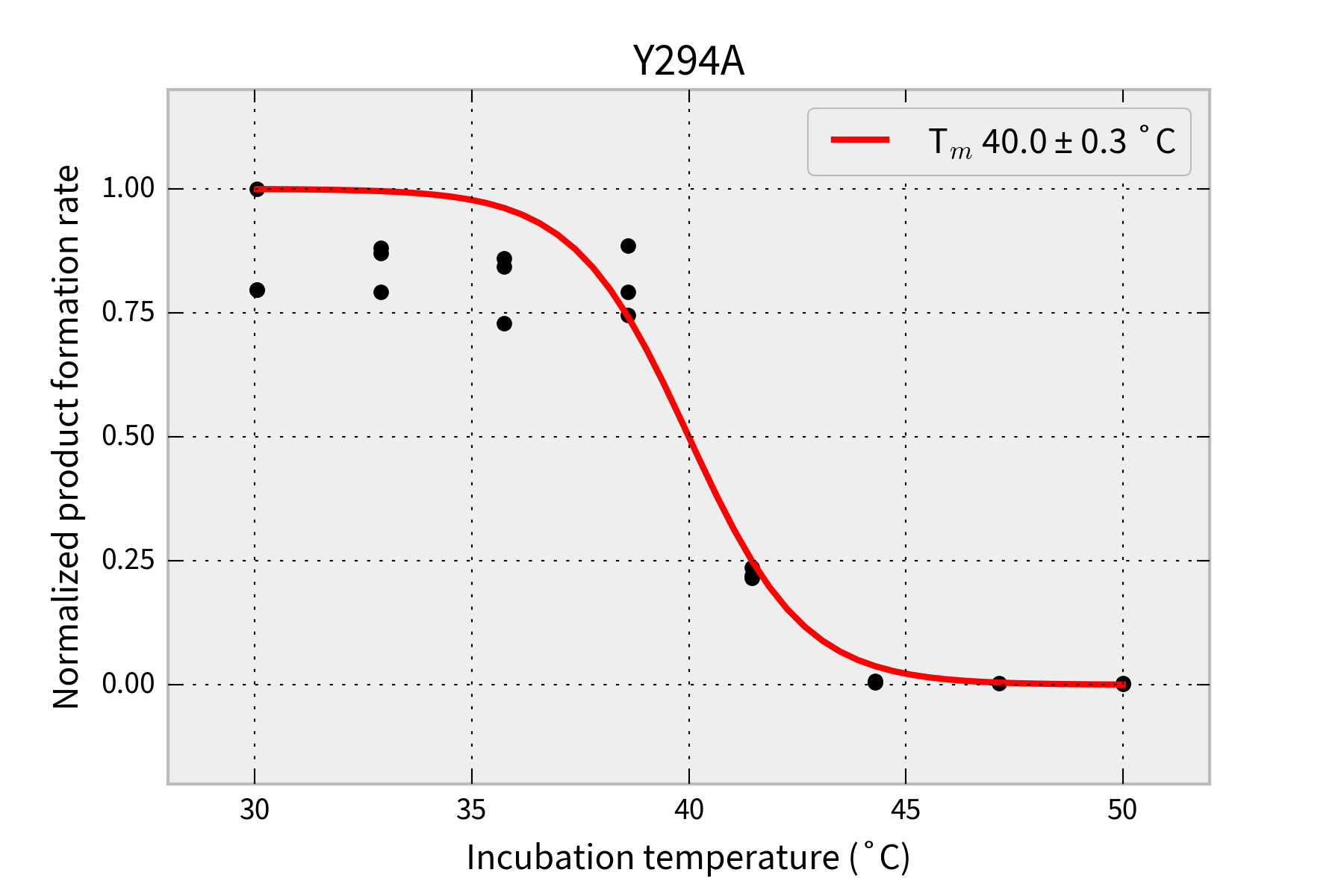

Supplement: S3 Figs — (ZIP) [file pone.0176255.s006.zip › S3 Figures/Y294A.png]

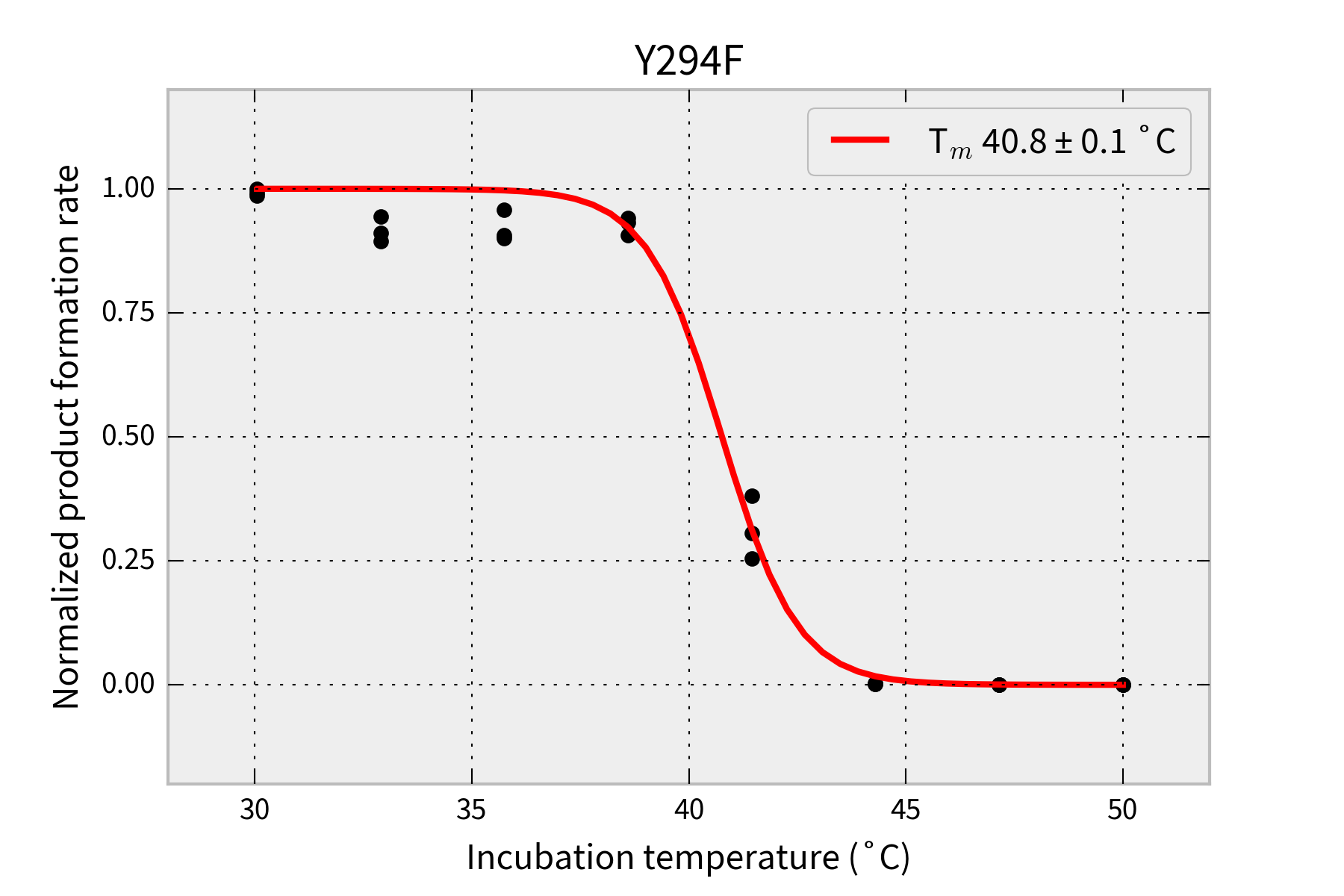

Supplement: S3 Figs — (ZIP) [file pone.0176255.s006.zip › S3 Figures/Y294F.png]
